# Synthesis of complex intermediates for the study of a dehydratase from borrelidin biosynthesis

**DOI:** 10.3762/bjoc.10.55

**Published:** 2014-03-11

**Authors:** Frank Hahn, Nadine Kandziora, Steffen Friedrich, Peter Francis Leadlay

**Affiliations:** 1 Institut für Organische Chemie und Biomolekulares Wirkstoffzentrum, Leibniz Universität Hannover, Schneiderberg 1B, 30167 Hannover, Germany; 2 Department of Biochemistry, University of Cambridge, 80 Tennis Court Road, Cambridge CB2 1GA, United Kingdom

**Keywords:** aldol reaction, coenzyme A, natural products, pig liver esterase, polyketide biosynthesis, protection groups

## Abstract

Herein, we describe the syntheses of a complex biosynthesis-intermediate analogue of the potent antitumor polyketide borrelidin and of reference molecules to determine the stereoselectivity of the dehydratase of borrelidin polyketide synthase module 3. The target molecules were obtained from a common precursor aldehyde in the form of *N*-acetylcysteamine (SNAc) thioesters and methyl esters in 13 to 15 steps. Key steps for the assembly of the polyketide backbone of the dehydratase substrate analogue were a Yamamoto asymmetric carbocyclisation and a Sakurai allylation as well as an *anti*-selective aldol reaction. Reference compounds representing the *E*- and *Z*-configured double bond isomers as potential products of the dehydratase reaction were obtained from a common precursor aldehyde by Wittig olefination and Still–Gennari olefination. The final deprotection of TBS ethers and methyl esters was performed under mildly acidic conditions followed by pig liver esterase-mediated chemoselective hydrolysis. These conditions are compatible with the presence of a coenzyme A or a SNAc thioester, suggesting that they are generally applicable to the synthesis of complex polyketide-derived thioesters suited for biosynthesis studies.

## Introduction

**Figure 1:**
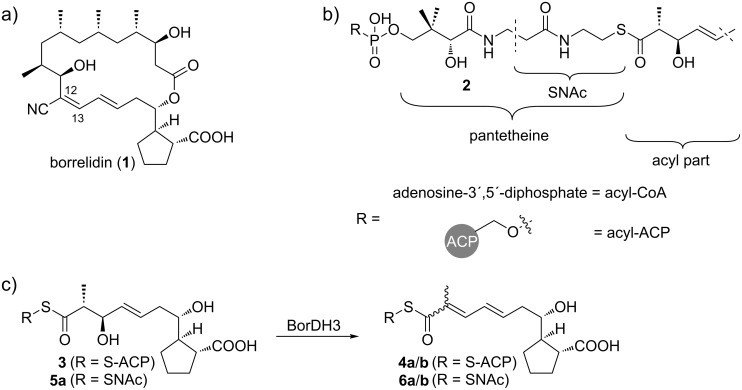
a) Structure of borrelidin (**1**); b) PKS intermediates are attached to an acyl carrier protein domain (ACP) via a 4'-phosphopantetheine linker, giving acyl-ACPs (**2**); c) BorDH3 catalyzes the dehydration of ACP-bound 3-hydroxyacylate **3** to one of the ACP-bound enoates **4a** or **4b**. The configuration of the double bond in the dehydration product is presently unknown. In a previous study, we have shown that BorDH3 only accepts surrogates with the shown 2D,3D-configuration if incubated with simple 3-hydroxy-2-methyl-SNAc pentanoates [7,12].

Borrelidin (**1**) is a macrolactone polyketide natural product with promising antibacterial, antimalarial, anticancer and anti-angiogenesis activities, which are probably caused by the inhibition of threonyl-tRNA synthetase and apoptosis induction by caspase activation [1–4]. It bears several unusual structural elements like a cyclopentane ring and a carbonitrile (Figure 1a), which are built-up by unconventional biosynthesis mechanisms [5–6]. The carbonitrile for example is probably formed by allylic oxidation of the 12-methyl group in 12-desnitrile-12-methylborrelidin to the corresponding aldehyde and transamination to the amine followed by oxidation [6]. In the course of our studies on borrelidin biosynthesis, we became interested in the formation of the (12*Z,*14*E*)-diene [7]. *Z*-configured double bonds are much rarer in polyketides than their *E*-configured counterparts, and their biosynthesis has not been thoroughly investigated yet [7–9].

Gene cluster analysis suggested that the double bond at position 12 in **1** is installed by the dehydratase of polyketide synthase (PKS) module 3 (BorDH3). Characteristic residues in the active site of the preceding ketoreductase point towards a 3D configuration of the BorDH3 precursor **3** [10–11]. Furthermore, we have shown in a previous study that BorDH3 preferentially accepts the 2D,3D-configured precursor, if all four potential stereoisomers of 3-hydroxy-2-methyl-SNAc-pentanoate model substrates are presented [7].

A commonly accepted model suggests that DHs from PKS I systems catalyze the removal of water by *syn*-dehydration and that a 2D,3D-configured precursor should lead to an *E*-configured BorDH3 product [10]. However, in the case of borrelidin this is in contradiction to the structure of the natural product in which a *Z*-configured double bond is present at position 12. In the borrelidin gene cluster, there is no obvious gene coding for an isomerase, which might be able to catalyze the inversion of a double bond configuration. Consequently, the *Z*-configured double bond must be installed either directly by BorDH3 or by *E*/*Z*-isomerisation of an initially formed *E*-configured double bond via a not yet elucidated mechanism in downstream biosynthetic processes.

**Scheme 1:**
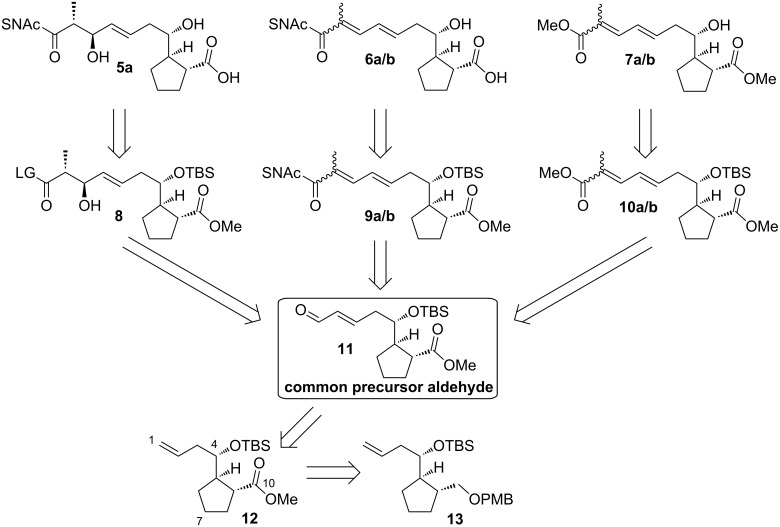
Retrosynthetic analysis of surrogate substrates for BorDH3 and reference molecules for enzyme assays (**5a**, **6a**, **6b**, **7a** and **7b**); TBS = *tert*-butyldimethylsilyl, PMB = *p*-methoxybenzyl, LG = leaving group.

Our aim was to assay the stereochemical course of the dehydratase of polyketide synthase (PKS) module 3 (BorDH3) in vitro. Therefore, the surrogate **5a** for BorDH3 as well as reference molecules such as **6a** and **6b** and the corresponding methyl esters **7a** and **7b**, which resemble the potential assay products or easily accessible derivatives of it, are required (Figure 1c, Scheme 1).

During chain elongation and reductive processing by PKSs, their intermediates are bound to acetyl carrier proteins (ACPs) via a 4'-phosphopantetheine arm (Figure 1b). It has been shown for other PKS domains that the recognition of this prosthetic group is essential for proper substrate orientation in the active site and catalysis with natural stereoselectivity [13]. To mimic the ACP-bound state of PKS intermediates, their analogues, free SNAc thioesters, are used in enzyme assays.

Alternatively, ACP-bound substrates can be conveniently obtained by loading coenzyme A (CoA) thioesters onto active site serine residues of recombinant ACPs by using 4'-phosphopantetheinyl transferases [12,14]. However, coenzyme A thioesters are synthetically hard to access, especially if the substrate structure is complex.

## Results and Discussion

### Retrosynthetic analysis of target molecules

One common feature of activated biosynthesis intermediate analogues such as **5a** is their relative low stability. In the natural context, PKS intermediates are quickly processed by downstream domains or tailoring enzymes. However, if analogues are synthesized chemically and isolated, it has to be taken into account that they often tend to undergo destructive side reactions. Therefore, mild reaction conditions are required, and the synthetic routes to them should preferably be organized in a divergent fashion that allows flexible access to all target compounds from a stable late-stage synthetic intermediate. For the synthesis of the target compounds presented in this study, a strategy via the common precursor aldehyde **11** was envisaged (Scheme 1).

The situation is additionally complicated by the fact that the polyketide part contains a cyclopentyl carboxylate, which necessitates differentiation of the carboxyl groups at the termini to permit regioselective thioester formation. We decided to avoid late redox transformations. Instead, we achieved differentiation by choice of a chemoselective protection group strategy with removal conditions that are compatible for SNAc thioesters (Scheme 1).

We envisaged the usage of TBS ether for the protection of the secondary hydroxy group and to protect the carboxylic acid as its methyl ester in the precursors **8**, **9a**, **9b**, **10a** and **10b**. These groups should be cleavable under mildly acidic or esterase-catalyzed conditions. As the presence of a methyl ester would prevent the selective introduction of one thioester into **5a** by saponification–thioesterification, we planned transesterification from a suitably activated carboxylic acid derivative **8**. Alternatively, direct introduction into **11** with appropriate SNAc thioester building blocks was planned for the synthesis of **6a** and **6b**.

Starting from aldehyde **11**, a common precursor for all molecules required in this study, aldol reaction and following transesterification should lead to thioester **8**. The bismethyl esters **7a** and **7b** as well as the SNAc thioesters **6a** and **6b** should be accessible from aldehyde **11** through a Horner–Wadsworth–Emmons reaction and the Still–Gennari olefination as well as by a Wittig olefination with stabilised phosphoranes, respectively. The aldehyde **11** should be accessible from the known molecule **13** via **12** [15].

### Synthesis of the common precursor aldehyde **11**

**Scheme 2:**
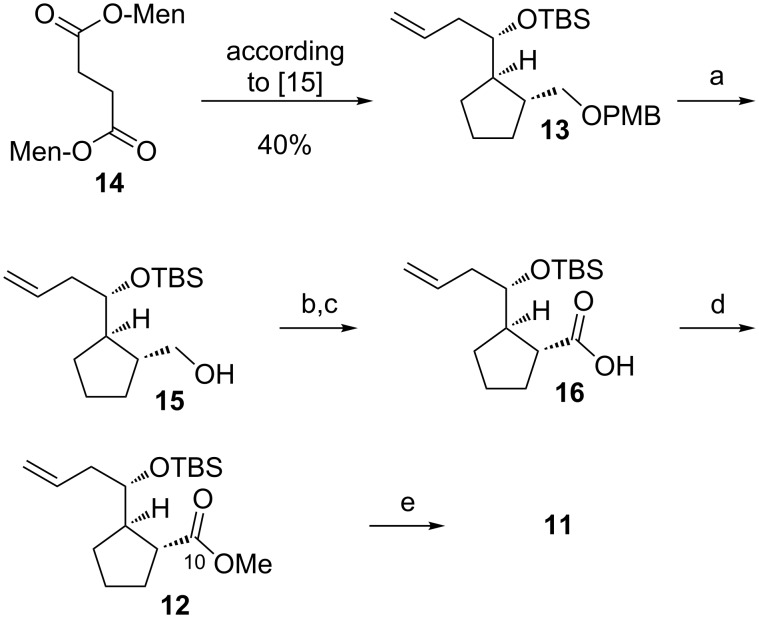
Synthesis of the common precursor aldehyde **11**. Compound **13** was prepared in six steps and with an overall yield of 40% via a known route by Omura et al. [15]. a) DDQ, CH_2_Cl_2_, rt, 30 min (85%); b) DMP, CH_2_Cl_2_, rt, 2 h; c) NaOCl, 2-methyl-2-butene, *t*-BuOH, phosphate buffer, rt, 16 h; d) TMS-CHN_2_, toluene/MeOH 2:3, rt, 40 min (58% over three steps); e) second generation Grubbs catalyst, crotonaldehyde, CH_2_Cl_2_, 40 °C, 120 min (88%); DDQ = 2,3-dichloro-5,6-dicyano-1,4-benzoquinone, Men = (*l*)-menthyl, DMP = Dess–Martin periodinane, TMS = trimethylsilyl.

The synthesis of the common precursor aldehyde **11** was accomplished from di(menth-1-yl)succinate (**14**) through a known route described by Omura et al*.* (Scheme 2) [15–17]. The final oxidation state at C10 in **12** was installed after protective group removal to primary alcohol **15** followed by Dess–Martin oxidation, Pinnick oxidation and methylation. Methyl ester **12** was obtained in 10 steps with a good overall yield of 20%. Olefin cross metathesis of alkene **12** with crotonaldehyde in the presence of a second generation Grubbs catalyst required only a short filter column to isolate α,β-unsaturated aldehyde **11** in a pure form.

### Synthesis of DH substrate surrogate

For the synthesis of the DH substrate surrogate **5a** we aimed at an *anti*-selective aldol reaction, which permits efficient access to the desired 2D,3D-stereoisomer followed by a smooth transformation into the SNAc thioester in the presence of a methyl ester function. Amongst others, we tested Paterson’s lactate-derived benzoyl auxiliary, Evans’ magnesium-catalyzed direct aldol reaction, and an Abiko–Masamune-like aldol reaction by using a thiodesoxy variant of the norephedrine-derived auxiliary on simplified model aldehydes as well as on aldehyde **11** [18–21]. However, in all these cases either the aldol reaction itself proved to be low yielding (<20%) or removal conditions of chiral auxiliaries were destructive to the molecule.

**Scheme 3:**
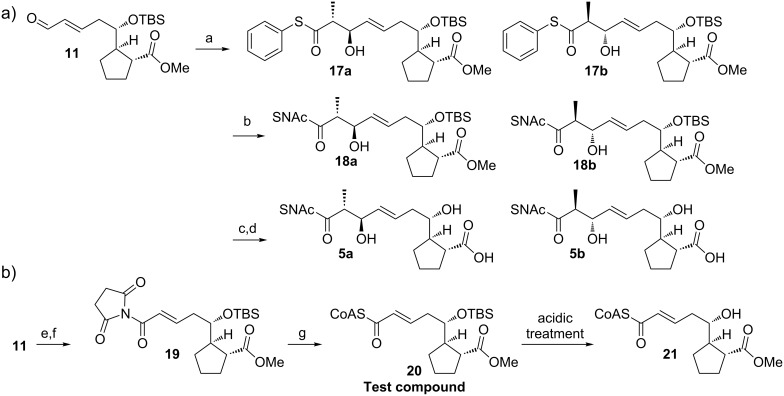
Synthesis of the BorDH3 substrates. a) Thiophenolpropionate, Cy_2_BCl, Me_2_EtN, Et_2_O, −78 °C to −20 °C, 16 h (57% over two steps from **12**); b) HSNAc, Et_3_N, DMF, rt, 16 h (70%); c) THF/HCOOH/H_2_O (6:3:1), rt, 2 d; d) pig liver esterase, phosphate buffer, rt, 5 d (81% over two steps); e) NaOCl, 2-methyl-2-butene, *t*-BuOH, phosphate buffer (pH 7.0), rt, 16 h; f) *N*-hydroxysuccinimide, *N*,*N’*-dicyclohexylcarbodiimide, THF, rt, 16 h (50% over three steps from **12**); g) HSCoA·3Li, THF/H_2_O (2:1) adjusted to pH 8.0 by phosphate buffer, 35 °C, 18 h; DMF = *N,N*-dimethylformamide.

The best results were obtained for an *anti*-selective variant, in which aldehyde **11** was reacted with an (*E*)-boron enolate formed by the reaction of thiophenol propionate with chlorodicyclohexylborane and dimethylethylamine, similar to the conditions described by Paterson et al. for lactate-derived auxiliaries [18]. In this way, the aldol product was conveniently obtained as an inseparable 1:1 mixture of both 2,3-*anti* diastereomers **17a** and **17b** in 57% yield over two steps [22]. Such thiophenol esters readily undergo thiol-exchange reactions and are therefore suitable precursors for SNAc, pantetheine and CoA thioesters [14]. Accordingly, when we treated the mixture of thiophenol esters **17a** and **17b** with HSNAc and triethylamine in DMF, they underwent clean transesterification furnishing SNAc thioesters **18a** and **18b** in 70% combined yield [20] (Scheme 3).

With the mixture of **18a** and **18b** in hand, we turned to the mild removal of the protection groups. For TBS cleavage, we focused on conditions previously described by us for the removal of an acid-sensitive dioxolane protection group from a CoA thioester [23]. We evaluated the exposure of the model CoA ester **20**, synthesized from **11** by Pinnick oxidation and CoA thioesterification via an intermediate activation as *N*-hydroxysuccinimide ester, to several acids in H_2_O/THF mixed solvent systems. In **20** the acyl part is similarly functionalized as in the protected SNAc thioesters **18a** and **18b**. However, the CoA thioester can be regarded as more demanding in terms of sensitivity.

The progress of the reaction was followed by mass spectrometry. While the use of strong acids like *p*-toluenesulfonic acid or hydrochloric acid led to the decomposition of **20**, weaker acids like acetic acid did not lead to any detectable product formation, even after reaction times of up to 20 h. Gratifyingly, treatment with a mixture of THF/HCOOH/H_2_O (6:3:1), gave a slow conversion into the desired product **21** (see Figure S1 and Figure S2 in Supporting Information File 1). Complete deprotection was achieved after 2 days.

The mixture of SNAc esters **18a** and **18b** was exposed to the established acidic deprotection conditions and the progress of the reaction was monitored by mass spectrometry. After two days, complete TBS removal was obtained. The crude product was subjected to selective methyl ester cleavage by using pig liver esterase (PLE) [24]. The target molecule was obtained as a mixture of both *anti*-diastereomers **5a** and **5b** after 15 steps in 7% overall yield.

### Synthesis of reference compounds

**Scheme 4:**
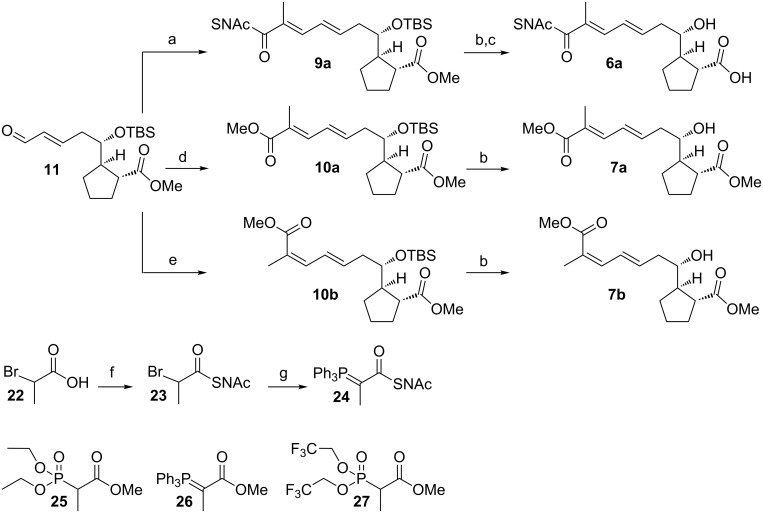
Synthesis of reference compounds for the BorDH3 assay. a) **24**, CH_2_Cl_2_, 50 °C, 3 h (88% over two steps); b) THF/HCOOH/H_2_O (6:3:1), rt, 2 d (62% for **7a**, 57% for **7b**); c) pig liver esterase, phosphate buffer, rt, 3 d (67% over two steps from **9a**); d) **25,** NaH, THF, 0 °C, 3 h (18% over two steps from **12**) or **26**, CH_2_Cl_2_, 50 °C, 21 h (64% over two steps from **12**); e) **27**, 18-crown-6, K_2_CO_3_, THF, −20 °C then 0 °C for 5 h (47% over two steps from **12**); f) HSNAc, EDC, DMAP, CH_2_Cl_2_, rt, 16 h (78%); g) PPh_3_, H_2_O, 70 °C, 11 h, then NaOH, rt (64%).

We exploited a Wittig reaction with a suitable stabilized phosphorane for the synthesis of (*E,E*)-diene **6a** (Scheme 4). The Wittig compound **24** was obtained in two steps from 2-bromopropionic acid following synthetic procedures described for its acetic acid analogue and the respective methyl ester ylide **26** [25–26]. The reaction of phosphorane **24** with **11** under neutral conditions at 50 °C gave the desired diene **9a** in very good 88% yield and in nearly perfect *E*-selectivity. The protection groups were removed from **9a** under the previously established conditions to finally give target SNAc thioester **6a** in a total yield of 12% over 14 steps. Alternative attempts to synthesize **9a** and **9b** by Horner–Wadsworth–Emmons olefination and Still–Gennari olefination with the respective phosphonates gave no reaction product at all (Horner–Wadsworth–Emmons olefination) or only the (*E*,*E)*-diene **10a** instead of the desired *E,Z*-configured product **10b** (Still–Gennari olefination).

In order to obtain alternative reference compounds for both potential products of the BorDH3 enzyme assay, we synthesized double bond isomers **7a** and **7b**. To be able to compare them to the products in the crude BorDH3 assay mixture, the latter will be transformed into their corresponding methyl esters by saponification and following methylation with trimethylsilyldiazomethane.

The fully protected *E*-isomer **10a** was obtained in 18% yield by a Horner–Wadsworth–Emmons reaction with phosphonate **25** or, alternatively, in 64% yield by a Wittig reaction with stabilized phosphorane **26**. The *Z*-isomer **10b** was synthesized by Still–Gennari olefination using phosphonate **27** in 47% yield. The yield for the deprotection step was satisfying (61% for **7a** and 57% for **7b**) in both cases, indicating that both isomers are configurationally stable under these conditions. The overall yield over 13 steps was 8% for **7a** and 5% for **7b**.

(*E,E*)-diene **6a** will find application in the comparative NMR evaluation of a large scale BorDH3 enzyme assay of substrates **5a** and **5b**. Together with the successful synthesis of **5a** and **5b** and the availability of the isomers **7a** and **7b**, this sets the stage to determine the stereoselectivity of BorDH3.

## Conclusion

We reported on the synthesis of a complex substrate and appropriate reference compounds for assaying the activity of the dehydratase in module 3 of the borrelidin polyketide synthase. All the target molecules **5a**, **6a**, **7a** and **7b** were obtained in 13 to 15 step sequences with overall yields of up to 12%. The routes diverged from a common precursor aldehyde **11**, which had been prepared in 18% yield over 11 steps, and had exploited the Yamamoto asymmetric carbocyclization and MgBr_2_-mediated Sakurai reaction to set up the three stereogenic centres.

The construction of the backbone was accomplished by *anti*-selective aldol reaction or olefination by using the respective phosphonates or phosphoranes. A notable achievement was the development of a chemoselective, mild protection strategy, which is based on an acidic treatment to cleave a silyl ether and a highly efficient and less common late stage ester hydrolysis by using pig liver esterase. Preliminary tests suggest the compatibility of these conditions with coenzyme A thioesters, indicating that the chosen strategy has broad potential in the future synthesis of similarly (or even more) complex polyketide-derived coenzyme A thioesters.

Thus, the presented strategy enables for the first time the in vitro assaying of a potential *Z*-selective dehydratase domain from a polyketide synthase with suitable precursor molecules. These experiments are currently ongoing in our lab.

## Supporting Information

The supporting information provides reaction details, analytical data, and copies of ^1^H and ^13^C NMR spectra.

File 1 Procedures.

File 2 NMR spectra.
