# Supplementary material for: Synthesis of complex intermediates for the study of a dehydratase from borrelidin biosynthesis
Source: Beilstein J Org Chem. 2014 Mar 11;10:634–40. doi: 10.3762/bjoc.10.55 (PMC3999764; doi:10.3762/bjoc.10.55)
Supplement: File 1 — Procedures. [file Beilstein_J_Org_Chem-10-634-s001.pdf]

Supporting information

for

Synthesis of complex intermediates for the study of a dehydratase from borrelidin biosynthesis

Frank Hahn*[,§1,2], Nadine Kandziora[§1], Steffen Friedrich[1] and Peter F. Leadlay[2]

Address: [1]Institut für Organische Chemie und Biomolekulares Wirkstoffzentrum, Leibniz Universität Hannover, Schneiderberg 1B, 30167 Hannover, Germany and [2]Department of Biochemistry, University of Cambridge, 80 Tennis Court Road, Cambridge CB2 1GA, United Kingdom

Email: Frank Hahn - frank.hahn@oci.uni-hannover.de

*Corresponding author

[§]These authors contributed equally

Procedures

Contents

General methods S2
Reaction details and analytical data S3
Figures S23
References supporting information S26

General methods

All reactions were performed in oven dried glassware under an atmosphere of Ar gas unless otherwise stated. Dry solvents were purchased from Sigma-Aldrich and Acros or taken out of a solvent system from M. Braun. Dry reagents were ordered from Sigma-Aldrich, Fluka, Arcos, ABCR and Roth. NMR spectra were recorded with Bruker DRX-500, DPX-400 and AVANCE-400 with the residual solvent signal as internal standard [1].The solvents are given with the data. Multiplicities are described by using the following abbreviations: s = singlet, d = doublet, t = triplet, q = quartet, m = multiplet, br = broad. ^{13}C NMR spectra are reported as values in ppm relative to residual solvent signal as internal standards [1]. The multiplicities are elucidated by using the distortionless enhancement by polarisation transfer (DEPT) spectral editing technique, with secondary pulses at 90° and 135°. Multiplicities are reported by using the following abbreviations: q (quarternary carbon), t (tertiary carbon = methine), s (secondary carbon = methylene), p = (primary carbon = methyl). High-resolution-mass spectra are obtained with a Micromass LCT via loop-mode injection from a Waters (Alliance 2695) HPLC system. Alternatively a Micromass Q-TOF in combination with a Waters Aquity Ultraperformance LC system is employed. Ionisation is achieved by ESI or APCI. Modes of ionisation, calculated and found mass are given. Reversed-phase HPLC applications were performed with membrane-filtrated and double distilled water as well as commercial available HPLC-grade solvents (methanol or acetonitrile), which have been degased with ultrasound. Preparative HPLC was operated at a Merck Hitachi LaChrome HPLC (Pump L-7150, Interface D-7000, Diode Array Detector L-7450). The following stationary phases were used: (C18-SP) Trentec Reprosil-Pur 120 C18 AQ 5µm, 250 mm * Ø 8 mm, corresponding precolumn cartridge, 40 mm * Ø 8 mm; (CN-SP) Trentec Reprosil 100 CN 5µm, 250 nm * Ø 8 mm, corresponding precolumn cartridge, 40 mm * Ø 8 mm. Alternatively preparative HPLC was performed with a Varian HPLC (Pumps Prepstar Model 218, Variable wavelength detector Prostar (λ = 248 nm) with parallel mass detection (Micromass Type ZMD ESI-Quad-Spectrometer) under use of a C18-$P_{[B]}$ stationary phase. Solvents, columns, operating procedures and retention times (t_R) are given with the corresponding experimental and analytical data. (Abbreviations: PE = petroleum ether; EtOAc = ethyl acetate). Known compounds are marked with the particular literature reference.

Reaction details and analytical data

((1*R*,2*R*)-Cyclopentane-1,2-diyl)dimethanol [2]

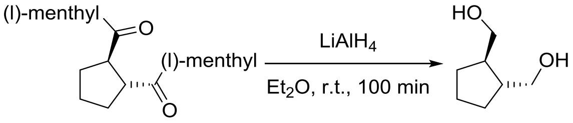

7.42 g (185.5 mmol, 2.8 equiv) $LiAlH_4$ were placed in a flask and 375 mL Et_2O were added. 28.80 g (66.3 mmol, 1.0 equiv) bis-(*l*)-menthylester in 125 mL Et_2O were added portionwise under vigorous stirring. The suspension was stirred at room temperature for 100 min. The reaction was cooled to 0 °C, quenched with 1 M HCl and saturated $NaHCO_3$ until the pH was 7. The aqueous layer was five times extracted with EtOAc. The combined organic extracts were dried over $MgSO_4$. The product was purified by flash chromatography on silica gel (Et_2O/hexane 1:1 then EtOAc). After drying in vacuo, 8.60 g (66.1 mmol) of a colorless oil were obtained (99% yield). About 17 g of menthol could be recovered from early fractions of the flash chromatography.

^{1}H NMR (400 MHz, $CDCl_3$) δ 4.59 (bs, 2 H, O*H*), 3.65 (dd, 2 H, J_1 = 2.7 Hz, J_2 = 10.4 Hz, CH_2OH(a)), 3.27 (dd, 2 H, J_1 = 8.8 Hz, J_2 = 9.0 Hz, CH_2OH(b)), 1.68-1.82 (m, 4 H, C*H*, CH_2CH(a)), 1.47-1.57 (m, 2 H, CH_2C$H_2$$CH_2$), 1.14-1.27 (m, 2 H, H-2, CH_2CH(b)); **^{13}C NMR** (100 MHz, $CDCl_3$) δ 66.3 (s, 2 C, *C*H_2OH), 47.9 (t, 2 C, *C*H), 29.7 (s, 2 C, *C*H_2CH), 23.9 (s, CH_2*C*$H_2$$CH_2$).

((1*R*,2*R*)-2-(((4-Methoxybenzyl)oxy)methyl)cyclopentyl)methanol [2]

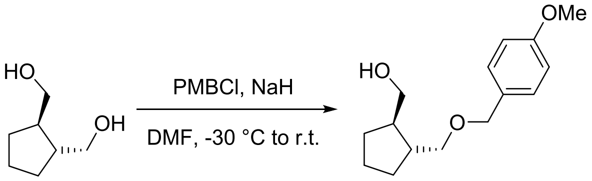

860 mg (6.60 mmol, 1 equiv) of ((1*R*,2*R*)-cyclopentane-1,2-diyl)dimethanol were solved in 36 mL DMF and cooled to −30 °C. 291 mg (7.27 mmol, 1.1 equiv) NaH in 30 mL DMF were added and the suspension was stirred for 40 min at −30 °C. 1.08 mL (7.93 mmol, 1.2 equiv) PMBCl were added and stirring was continued while the solution was allowed to warm to room temperature. Water was then added and the aqueous layer was three times extracted with Et_2O. The combined organic layers were three times washed with a 10% volume of water. The organic layer was dried over Na_2SO_4. The product was purified by flash chromatography on silica gel (hexane/EtOAc 20:1 then hexane/EtOAc 8:1). After drying in vacuo, 1.45 g (5.79 mmol) of the desired alcohol was obtained in form of a slightly yellow oil (88% yield).

^{1}H NMR (400 MHz, $CDCl_3$) δ 7.27 (d, 2 H, *J* = 8.7 Hz, C*H*(ar)), 6.90 (d, 2 H, *J* = 8.6 Hz, C*H*(ar)), 4.50 (s, 2H, CH_2(OPMB)), 3.81 (s, 3 H, OCH_3), 3.63 (dd, 1 H, J_1 = 4.0 Hz, J_2 = 10.5 Hz, 1x CH_2OH), 3.57 (dd, 1 H, J_1 = 4.3 Hz, J_2 = 8.7 Hz, 1x CH_2OH), 3.37 (dd, 1 H, J_1 = 8.8 Hz, J_2 = 10.7 Hz, 1x CH_2OPMB), 3.25 (dd, 1 H, J_1 = 9.1 Hz, J_2 = 9.2 Hz, 1x CH_2OPMB), 1.74-1.97 (m, 3 H, C*H*CH_2OH, C*H*CH_2OPMB, 1x $CH_2CH_2CHCH_2$OH), 1.53-1.62 (m, 2 H, $CH_2CH_2CHCH_2$OH), 1.21-1.33 (m, 3 H, 1x CH_2CH_2CHOH, CH_2CHCH_2OPMB); **^{13}C NMR** (100 MHz, $CDCl_3$) δ 159.3 (q, 1 C, $COCH_3$), 129.7 (q, 1 C, CCH_2O), 129.5 (t, 2 C, *C*H(ar)), 113.9 (t, 2 C, *C*H(ar)), 74.3 (s, 1 C, CH_2OPMB), 73.0 (s, 1 C, CH_2(PMB)), 67.0 (s, 1 C, CH_2OH), 55.2 (p, 1 C, OCH_3), 48.3 (t, 1 C, $CHCH_2$OH), 45.3 (t, 1 C, $CHCH_2$OPMB), 30.0 (s, 2 C, $CH_2CH_2CH_2$, $CH_2CH_2CH_2$), 24.0 (s, 1 C, $CH_2CH_2CH_2$).

(1*R*,2*R*)-2-(((4-Methoxybenzyl)oxy)methyl)cyclopentane-1-carbaldehyde [2]

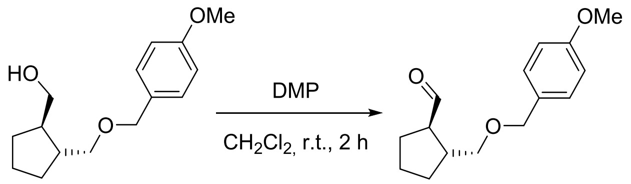

4.80 g (19.18 mmol, 1.0 equiv) of the precursor alcohol in 150 mL CH_2Cl_2 were added to 16.95 g (39.95 mmol, 2.0 equiv) Dess–Martin periodinane. After stirring for 2 h at room temperature, the reaction was quenched with 200 mL Et_2O, 100 mL $NaHCO_3$ and 10 g $Na_2S_2O_3$ and stirred until the organic layer became clear. The organic layer was three times extracted with Et_2O. The combined organic layers were washed with 200 mL saturated $NaHCO_3$ and 200 mL brine. The product was purified by flash chromatography on silica gel (hexane/EtOAc 40:1 then hexane/EtOAc 10:1 then EtOAc). After drying in vacuo, 4.43 g (17.84 mmol) of the PMB-protected aldehyde were obtained in form of a yellow oil (93% yield).

^{1}H NMR (200 MHz, $CDCl_3$) δ 9.65 (d, 1 H, *J* = 2.4 Hz, C*H*O), 7.24 (d, 2 H, *J* = 8.6 Hz, C*H*(ar)), 6.87 (d, 2 H, *J* = 8.6 Hz, C*H*(ar)), 4.44 (s, 2 H, CH_2-PMB), 3.81 (s, 3 H, OMe), 3.47 (dd, 1 H, J_1 = 5.8 Hz, J_2 = 9.1 Hz, 1x $CHCH_2$O), 3.33 (dd, 1 H, J_1 = 7.2 Hz, J_2 = 9.1 Hz, 1x $CHCH_2$O), 2.39-2.61 (m, 1 H, CH_2CHCH_2O), 1.76-1.94 (m, 3 H, C*H*CHO,C*H*CH_2O, 1x CH_2CHCHO), 1.57-1.76 (m, 2 H, CH_2CH_2CHCHO), 1.33-1.47 (m, 1 H, 1x CH_2CHCHO); **^{13}C NMR** (100 MHz, $CDCl_3$) δ 203.6 (q, 1 C, *C*HO), 159.1 (q, 1 C, *C*(ar)), 130.4 (q, 1 C, *C*(ar)), 129.3 (t, 2 C, *C*(ar)), 113.7 (t, 2 C, *C*(ar)), 72.9 (s, 1 C, CH_2O), 72.6 (s, 1 C, CH_2-OPMB), 55.7 (t, 1 C, *C*HCHO), 55.2 (p, 1 C, OMe), 41.2 (t, 1 C, $CHCH_2$O), 29.3 (s, 1 C, $CH_2CHCHCH_2$O), 26.5 (s, 1 C, CH_2CHCHO), 24.9 (s, 1 C, $CH_2CH_2CH_2$).

(*S*)-1-((1*R*,2*R*)-2-(((4-Methoxybenzyl)oxy)methyl)cyclopentyl)but-3-en-1-ol [2]

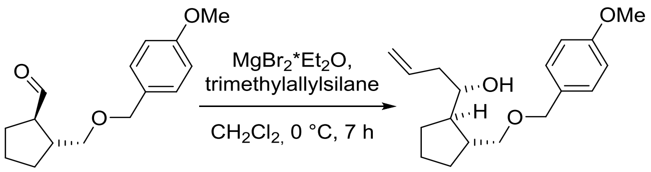

6.15 g (24.8 mmol, 1.0 equiv) of the precursor aldehyde were solved in 250 mL CH_2Cl_2 and cooled to 0 °C. 6.40 g (24.8 mmol, 1.0 equiv) $MgBr_2 \cdot Et_2O$ followed by 5.93 mL (37.2 mmol, 1.5 equiv) trimethylallylsilane were added. After stirring at 0 °C for 7 h, the reaction was quenched with a 1:1 mixture of methanol and 1 M NaOH and stirred for 30 min. The organic layer was two times extracted with CH_2Cl_2 and two times with Et_2O. The combined organic layers were dried over $MgSO_4$. The product was purified by flash chromatography on silica gel (hexane then hexane/EtOAc 15:1 then hexane/EtOAc 10:1). After drying in vacuo, 4.74 g (16.3 mmol) of the secondary alcohol in form of a yellow oil were obtained (66% yield).

$\mathbf{R_f}$ = 0.1 (PE/EtOAc 10:1); $[\alpha]_D^{21}$ = -17.7 (*c* = 0.5, CH_2Cl_2); **^{1}H NMR** (200 MHz, $CDCl_3$) δ 7.22-7.28 (m, 2 H, C*H*(ar)), 6.86-6.90 (m, 2 H, C*H*(ar)), 5.87-6.07 (m, 1 H, CH_2=C*H*), 5.08-5.15 (m, 1 H, CH_2=CH), 5.04-5.06 (m, 1 H, CH_2=CH), 4.49-5.06 (m, 2 H, CH_2(OPMB)), 3.81 (s, 3 H, OCH_3), 3.32-3.46 (m, 2 H, CH_2OPMB), 3.19 (dd, J_1 = 8.8 Hz, J_2 = 9.99 Hz, 1 H, C*H*OH), 2.03-2.18 (m, 2 H, CH_2), 1.19-1.85 (m, 8 H, 3x CH_2(cyclopentane), 2x C*H*).

***tert*-Butyl(((*S*)-1-((1*R*,2*R*)-2-(((4-methoxybenzyl)oxy)methyl)cyclopentyl)but-3-en-1-yl)oxy)dimethylsilane (13)[2]**

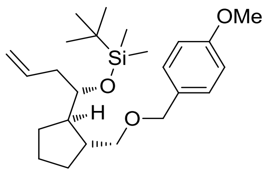

4.74 g (16.3 mmol, 1.0 equiv) of the product from the Sakurai reaction were solved in 164 mL CH_2Cl_2 and cooled to 0 °C. 2.86 mL (24.5 mmol, 1.5 equiv) of 2,6-lutidine and 4.88 mL (21.3 mmol, 1.3 equiv) of TBSOTf were added successively. The solution was stirred for 1 h, and an equal volume of water was added. The aqueous layer was three times extracted with CH_2Cl_2, and the combined organic layers were dried over anhydrous Na_2SO_4. The crude product was purified by flash chromatography on silica gel (hexane/EtOAc 50:1). After drying in vacuo, 6.25 g (15.9 mmol) of the yellow oil **13** were obtained (98% yield).

$\mathbf{R_f}$ = 0.3 (PE/EtOAc 50:1); **^{1}H NMR** (400 MHz, $CDCl_3$) δ 7.27 (d, 2 H, *J* = 8.8 Hz, C*H*(ar)), 6.88 (d, 2 H, *J* = 8.8 Hz, C*H*(ar)), 5.85 (dddd, 1 H, J_1 = 16.0 Hz, J_2 = 11.9 Hz, J_3 = 6.9 Hz, J_4 = 6.0 Hz, CH_2=C*H*), 5.04 (t, 1 H, *J* = 1.6 Hz, 1x CH_2=CH), 4.98-5.02 (m, 1 H, 1x CH_2=CH), 4.46 (d, 1 H, *J* = 11.6 Hz, 1x CH_2-PMB), 4.41 (d, 1 H, J_1 =

11.6 Hz, 1x C*H*$_2$-PMB), 3.81 (s, 3 H, OMe), 3.53 (dd, 1 H, J_1 = 5.4 Hz, J_2 = 10.7 Hz, C*H*OTBS), 3.46 (dd, 1 H, J_1 = 5.0 Hz, J_2 = 8.8 Hz, 1x CHC*H*$_2$O), 3.18 (dd, 1 H, J_1 = 8.9 Hz, J_2 = 8.9 Hz, 1x CHC*H*$_2$O), 2.10-2.33 (m, 3 H, CH$_2$=CHC*H*$_2$, C*H*CH$_2$O), 1.63-1.76 (m, 3 H, 1x C*H*$_2$CH$_2$CH$_2$, 1x CH$_2$C*H*$_2$CH$_2$, 1x CH$_2$CH$_2$C*H*$_2$), 1.41-1.63 (m, 3 H, C*H*CHOTBS, 1x C*H*$_2$CH$_2$CH$_2$, 1x CH$_2$C*H*$_2$CH$_2$), 1.24-1.35 (m, 1 H, 1x CH$_2$CH$_2$C*H*$_2$), 0.90 (m, 9 H, OSi(CH$_3$)$_2$C(C*H*$_3$)$_3$), 0.07 (m, 3 H, 1x OSi(C*H*$_3$)$_2$C(CH$_3$)$_3$), 0.06 (m, 3 H, 1x OSi(C*H*$_3$)$_2$C(CH$_3$)$_3$); **^{13}C NMR** (100 MHz, CDCl$_3$) δ 159.0 (q, 1 C, *C*(ar)), 135.4 (t, 1 C, CH$_2$=*C*H), 131.0 (q, 1 C, *C*(ar)), 129.1 (t, 2 C, *C*(ar)), 116.5 (s, 1 C, *C*H$_2$=CH), 113.7 (t, 2 C, *C*(ar)), 75.5 (t, 1 C, *C*HOTBS), 74.7 (s, 1 C, CH*C*H$_2$O), 72.6 (s, 1 C, *C*H$_2$-PMB), 55.2 (p, 1 C, OMe), 46.8 (t, 1 C, *C*HCHOTBS), 41.0 (s, 1 C, CH$_2$=CH*C*H$_2$), 39.8 (t, 1 C, *C*HCH$_2$O), 30.6 (s, 1 C, CH$_2$CH$_2$*C*H$_2$), 29.5 (s, 1 C, *C*H$_2$CH$_2$CH$_2$), 25.3-25.9 (s, p, 4 C, CH$_2$*C*H$_2$CH$_2$, OSi(CH$_3$)$_2$C(*C*H$_3$)$_3$), 18.1 (s, 1 C, OSi(CH$_3$)$_2$*C*(CH$_3$)$_3$), -4.2 (p, 1 C, OSi(*C*H$_3$)$_2$C(CH$_3$)$_3$) , -4.6 (p, 1 C, OSi(*C*H$_3$)$_2$C(CH$_3$)$_3$).

((1*R*,2*R*)-2-((*S*)-1-((*tert*-Butyldimethylsilyl)oxy)but-3-en-1-yl)cyclopentyl)methanol (15)

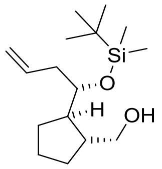

6.60 g (16.8 mmol, 1.0 equiv) of TBS-protected compound **13** were solved in 300 mL CH_2Cl_2 and 60 mL water and cooled to 0 °C. 4.68 g (20.2 mmol, 1.2 equiv) of DDQ were added and stirred for 30 min at 0 °C. The orange solution was poured into 250 mL water, and the aqueous layer was twice extracted with EtOAc. The combined organic layers were dried over Na_2SO_4, and the crude product was purified by flash chromatography on silica gel (hexane then hexane/EtOAc 20:1). After drying in vacuo, 5.27 g (14.2 µmol) of the yellow oil **15** were obtained (85% yield).

R_f = 0.2 (PE/EtOAc 50:1); **^{1}H NMR** (200 MHz, CDCl$_3$) δ 5.83-5.94 (m, 1 H, CH$_2$=C*H*), 5.07 (bs, 1 H, C*H*$_2$=CH), 5.03 (d, *J* = 8.2 Hz, 1 H, C*H*$_2$=CH), 3.48-3.58 (m, 2 H, C*H*$_2$OH), 3.33-3.38 (m, 1 H, C*H*OTBS), 2.61 (bs, 1 H, O*H*), 2.34-2.41 (m, 1 H, C*H*$_2$CHOTBS), 2.19-2.27 (m, 1 H, C*H*$_2$CHOTBS), 2.00-2.08 (m, 1 H, C*H*CH$_2$OH), 1.80-1.88 (m, 1 H, CH$_2$CH$_2$C*H*), 1.65-1.76 (m, 2 H, C*H*$_2$CHCH$_2$OH), 1.53-1.61 (m, 1 H, CH$_2$C*H*$_2$CH), 1.41-1.50 (m, 1 H, C*H*$_2$CH$_2$CH), 1.16-1.37 (m, 3 H, CH$_2$C*H*$_2$CH, C*H*$_2$CH$_2$CH), 0.90 (s, 9 H, OSi(CH$_3$)$_2$C(C*H*$_3$)$_3$), 0.09 (s, 6 H, OSi(C*H*$_3$)$_2$C(CH$_3$)$_3$); **^{13}C NMR** (100 MHz, CDCl$_3$) δ 134.0 (t, 1 C, CH$_2$=*C*H), 117.2 (s, 1 C, *C*H$_2$=CH), 76.2 (t, 1 C, *C*HOTBS), 67.2 (s, 1 C, *C*H$_2$OH), 47.7 (t, 1 C, CH$_2$CH$_2$*C*H), 43.8 (t, 1 C, *C*HCH$_2$OH), 40.0 (s, 1 C, *C*H$_2$CHOTBS), 31.6 (s, 1 C, CH$_2$CH$_2$CH$_2$CH), 30.8 (s, 1 C, CH$_2$CH$_2$CH$_2$CH), 30.0 (s, 1 C, CH$_2$CH$_2$CH$_2$CH), 26.0 (p, 3 C, OSi(CH$_3$)$_2$C(*C*H$_3$)$_3$), 18.1 (q, 1 C, OSi(CH$_3$)$_2$*C*(CH$_3$)$_3$), -4.3 (s, 2 C, OSi(*C*H$_3$)$_2$C(CH$_3$)$_3$); **HRMS (ESI)** *m/z* calculated for $C_{16}H_{32}NO_2SiNa$ $[M+H]^+$: 307.2069, found: 307.2080.

(1*R*,2*R*)-2-((*S*)-1-((*tert*-Butyldimethylsilyl)oxy)but-3-en-1-yl)cyclopentane-1-carbaldehyde

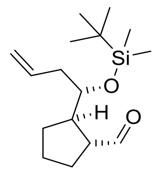

5.27 g (14.2 mmol, 1 equiv) of primary alcohol **15** were solved in 130 mL CH_2Cl_2 and 17.2 g (28.4 mmol, 2.0 equiv) DMP were added. After stirring for 1 h at room temperature, 130 mL Et_2O as well as a 1:1 mixture of a saturated $NaHCO_3$ and a 1.5 M $Na_2S_2O_3$ solution was added. The solution was stirred until both layers became clear. After the separation of the layers, the aqueous layer was extracted with Et_2O. The combined organic layers were washed with water and a saturated NaCl solution. After drying over $MgSO_4$, the crude product was purified by flash chromatography on silica gel (hexane then hexane/EtOAc 30:1 then hexane/EtOAc 20:1). Drying in vacuo gave 2.81 g (9.95 mmol) of an yellow oil (70% yield).

$\mathbf{R_f}$ = 0.3 (PE/EtOAc 50:1); **^{1}H NMR** (200 MHz, $CDCl_3$) δ 9.61 (d, *J* = 2.6 Hz, 1 H, C*H*O), 5.78-5.89 (m, 1 H, CH_2=C*H*), 5.02-5.08 (m, 2 H, CH_2=CH), 3.65-3.69 (m, 1 H, C*H*OTBS), 2.70-2.77 (m, 1 H, C*H*CHO), 2.39 (qt, J = 7.5 Hz, 1H, C*H*), 2.23-2.29 (m, 2 H, CH_2), 1.19-1.91 (m, 8 H, 3x CH_2 (cyclopentane), C*H*), 0.88 (s, 9 H, $OSi(CH_3)_2C(CH_3)_3$), 0.07 (d, *J* = 3.2 Hz, 6 H, $OSi(CH_3)_2C(CH_3)_3$); **^{13}C NMR** (100 MHz, $CDCl_3$) δ 202.3 (t, 1 C, *C*HO), 132.2 (t, 1 C, CH_2=*C*H), 115.5 (s, 1 C, *C*H_2=CH), 72.9 (t, 1 C, *C*HOTBS), 51.6 (t, 1 C, CH_2CH_2*C*H), 43.3 (t, 1 C, *C*HCHO), 38.7 (s, 1 C, *C*H_2CHOTBS), 28.0 (s, 1 C, *C*$H_2CH_2CH_2$CH), 25.6 (s, 1 C, CH_2CH_2*C*H_2CH), 24.0 (s, 1 C, CH_2*C*H_2CH_2CH), 23.9 (p, 3 C, $OSi(CH_3)_2C(CH_3)_3$), 16.1 (q, 1 C, $OSi(CH_3)_2C(CH_3)_3$), -5.9 (s, 1 C, $OSi(CH_3)_2C(CH_3)_3$), -6.5 (s, 1 C, $OSi(CH_3)_2C(CH_3)_3$).

(1*R*,2*R*)-2-((*S*)-1-((*tert*-Butyldimethylsilyl)oxy)but-3-en-1-yl)cyclopentane-1-carboxylic acid (16)

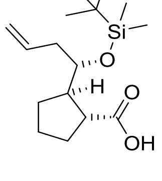

1.00 g (3.70 mmol, 1.0 equiv) of the precursor aldehyde was solved in a mixture of 80 mL *t*-BuOH and 20 mL 2-methyl-2-butene. After the addition of 2.68 g (29.6 mmol, 8.0 equiv) $NaClO_2$ in 15 mL phosphate buffer (pH 7.0), the mixture was stirred for 16 h at room temperature. *tert*-Butanol was removed under reduced pressure, and 160 mL water were added. This aqueous solution was three times extracted with EtOAc, the combined organic layers were dried over $MgSO_4$, and the solvent was removed

under reduced pressure. Drying in vacuo gave 1.08 g (3.7 mmol) of the yellow oil **16**, which was subjected to the next step without further purification.

R*f* = 0.5 (PE/EtOAc 9:1); **[α]$_D$^{22}**= -5.8 (*c* = 1.2, CH_2Cl_2); **^{1}H NMR** (400 MHz, MeOD) δ 5.79-5.90 (m, 1 H, CH_2=C*H*), 5.04-5.07 (m, J_2 = 10.6 Hz, 1 H, C*H$_2$*CH), 5.02 (d, *J* = 1.4 Hz, 1 H, C*H$_2$*CH), 3.70-3.74 (m, 1 H, C*H*OTBS), 2.68-2.74 (m, 1 H, C*H*COOH), 2.46-2.53 (m, 1 H, CH_2C*H*COOH), 2.19-2.34 (m, 2 H, C*H$_2$*CHOTBS), 1.87-1.96 (m, 1 H, C*H$_2$*CHCOOH), 1.74-1.83 (m, 2 H, C*H$_2$*CHCOOH, CH_2C*H$_2$*CH), 1.63-1.69 (m, 2 H, C*H$_2$*CH_2CH), 1.42-1.47 (m, 1 H, CH_2C*H$_2$*CH), 0.91 (s, 9 H, OSi(CH_3)$_2$C(C*H$_3$*)$_3$), 0.09 (s, 6 H, OSi(C*H$_3$*)$_2$C(CH_3)$_3$); **^{13}C NMR** (100 MHz, MeOD) δ 181.0 (q, 1 C, *C*OOH), 136.0 (t, 1 C, CH_2=*C*H), 117.6 (s, 1 C, *C*H$_2$=CH), 76.1 (t, 1 C, *C*HOTBS), 49.0 (t, 1 C, CH_2CH_2*C*H), 46.2 (t, 1 C, *C*HCOOH), 41.4 (s, 1 C, *C*H$_2$CHOTBS), 33.1 (s, 1 C, *C*H$_2$CHCOOH), 30.9 (s, 1 C, *C*H$_2$$CH_2$CH), 27.0 (s, 1 C, CH_2*C*H$_2$CH), 26.5 (p, 3 C, OSi(CH_3)$_2$C(*C*H$_3$)$_3$), 19.0 (q, 1 C, OSi(CH_3)$_2$*C*(CH_3)$_3$), -3.8 (p, 1 C, OSi(*C*H$_3$)$_2$C(CH_3)$_3$), -4.5 (p, 1 C, OSi(*C*H$_3$)$_2$C(CH_3)$_3$); **HRMS (ESI)** *m/z* calculated for $C_{16}H_{30}O_3SiNa$ [M+Na]$^+$: 321.1862, found: 321.1862.

Methyl (1*R*,2*R*)-2-((*S*)-1-((*tert*-butyldimethylsilyl)oxy)but-3-en-1-yl)cyclopentane-1-carboxylate (12)

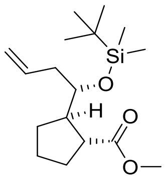

1.08 g (3.7 mmol, 1.0 equiv) of the precursor acid **16** were solved in 3.8 mL of a mixture of toluene/methanol 3:2 and 2.70 mL (5.4 mmol, 1.4 equiv, 2 M in Et_2O) $TMSCHN_2$ were added dropwise under continuous stirring until the color of the solution remained yellow. After stirring for 40 min, all volatiles were removed under reduced pressure. The crude product was purified by flash chromatography on silica gel (cyclohexane/EtOAc 30:1 then cyclohexane/EtOAc 20:1 then cyclohexane/EtOAc 5:1). After drying in vacuo, 925 mg (3.1 mmol) of the colorless oil **12** were obtained (83% yield over two steps).

R*f* = 0.54 (PE/EtOAc 9:1); **[α]$_D$^{21}** = +0.8 (*c* = 0.6, CH_2Cl_2); **^{1}H NMR** (400 MHz, $CDCl_3$) δ 5.74-5.85 (m, 1 H, CH_2=C*H*), 5.03 (d, *J* = 1.0 Hz, 1 H, C*H$_2$*=CH), 4.98-5.02 (m, 1 H, C*H$_2$*=CH), 3.64-3.66 (m, 1 H, C*H*OTBS), 3.65 (s, 3 H, COOC*H$_3$*), 2.73-2.79 (m, 1 H, C*H*COOCH$_3$), 2.45-2.52 (m, 1 H, CH_2CH_2C*H*), 2.20-2.24 (m, 2 H, C*H$_2$*CHOTBS), 1.82-1.91 (m, 1 H, C*H$_2$*CHCOOCH$_3$), 1.72-1.80 (m, 2 H, C*H$_2$*CHCOOCH$_3$, CH_2C*H$_2$*CH), 1.59-1.66 (m, 2 H, C*H$_2$*CH_2CH), 1.35-1.44 (m, 1 H, CH_2C*H$_2$*CH), 0.87 (s, 9 H, OSi(CH_3)$_2$C(C*H$_3$*)$_3$), 0.06 (s, 3 H, OSi(C*H$_3$*)$_2$C(CH_3)$_3$), 0.05 (s, 3 H, OSi(C*H$_3$*)$_2$C(CH_3)$_3$); **^{13}C NMR** (100 MHz, $CDCl_3$) δ 177.7 (q, 1 C, *C*OOCH$_3$), 134.9 (t, 1 C, CH_2=*C*H), 117.0 (t, 1 C, *C*H$_2$=CH), 74.5 (t, 1 C, *C*HOTBS), 51.5 (p, 1 C, COO*C*H$_3$), 47.4 (t, 1 C, CH_2CH_2*C*H), 44.4 (t, 1 C, *C*HCOOCH$_3$), 40.4 (s, 1 C,

CH$_2$CHOTBS), 32.1 (s, 1 C, $CH_2CHCOOCH_3$), 30.0 (s, 1 C, CH_2CH_2CH), 26.2 (s, 1 C, CH_2CH_2CH), 25.9 (p, 3 C, $OSi(CH_3)_2C(CH_3)_3$), 18.0 (q, 1 C, $OSi(CH_3)_2C(CH_3)_3$), -4.1 (p, 1 C, $OSi(CH_3)_2C(CH_3)_3$), -4.7 (p, 1 C, $OSi(CH_3)_2C(CH_3)_3$); **HRMS (ESI)** *m/z* calculated for $C_{17}H_{32}O_3NaSi$ $[M+Na]^+$: 335.2018, found: 335.2020.

Methyl (1*R*,2*R*)-2-((*S*,*E*)-1-((*tert*-butyldimethylsilyl)oxy)-5-oxopent-3-en-1-yl)cyclopentane-1-carboxylate (11)

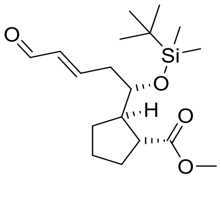

200 mg (640 µmol, 1.0 equiv) **12** and 1.30 mL (15.8 mmol, 25 equiv) crotonaldehyde were solved in 40 mL CH_2Cl_2. 27 mg (32 µmol, 5 mol %) Grubbs II catalyst were added, and the mixture was heated to 40 °C under reflux for 2 h. All volatiles were removed under reduced pressure, and the crude product was purified by flash chromatography on silica gel (petroleum ether/EtOAc 9:1). After drying in vacuo, 192 mg (0.57 mmol) of a brown oil **11** were obtained (88% crude yield). Due to the oxidation sensitivity of unsaturated aldehyde **11**, the compound was only analyzed by ^{1}H NMR spectroscopy and routinely subjected into the following reactions only after TLC analysis and passing the crude product through a short filter column of silica gel. The amount that was inserted into the following reactions and overall yield calculations based on the assumption of quantitative conversion in this step.

$\mathbf{R}_f$ = 0.33 (PE/EtOAc 9:1); **^{1}H NMR** (200 MHz, $CDCl_3$) δ 9.51 (d, *J* = 7.9 Hz, 1 H, *H*CO), 6.89 (dt, J_1 = 7.3 Hz, J_2 = 15.6 Hz, 1 H, HC(O)C*H*), 6.05-6.18 (m, 1 H, HC(O)CHC*H*), 3.76-3.86 (m, 1 H, C*H*OTBS), 3.65 (s, 3 H, COOC*H$_3$*), 2.67-2.78 (m, 1 H, C*H*COOCH$_3$), 2.39-2.55 (m, 3 H, CH$_2$CH$_2$C*H*, C*H$_2$*CHOTBS), 1.57-1.97 (m, 6 H, C*H$_2$*C*H$_2$*CH, C*H$_2$*CHCOOCH$_3$), 0.88 (s, 9 H, OSi(CH$_3$)$_2$C(C*H$_3$*)$_3$), 0.07 (s, 3 H, OSi(C*H$_3$*)$_2$C(CH$_3$)$_3$), 0.06 (s, 3 H, OSi(C*H$_3$*)$_2$C(CH$_3$)$_3$).

(*S,E*)-5-((*tert*-Butyldimethylsilyl)oxy)-5-((*1R,2R*)-2-(methoxycarbonyl)cyclopentyl)pent-2-enoic acid

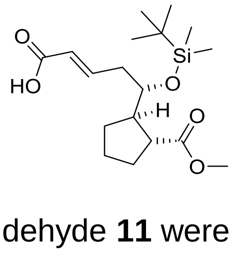

200 mg (590 µmol, 1.0 equiv) of aldehyde **11** were solved in 16 mL *t*-BuOH and 4 mL 2-methyl-2-butene. After the addition of 425 mg (4.69 mmol, 8.0 equiv) $NaClO_2$ and 3 mL phosphate buffer (pH = 7), the clear solution was stirred for 16 h. *tert*-Butanol and

2-methyl-2-butene were removed under reduced pressure, and 30 mL water were added. The aqueous layer was three times extracted with EtOAc. The combined organic layers were dried over $MgSO_4$, and the solvent was removed under reduced pressure. After drying in vacuo, 192 mg (540 µmol) of a yellow oil were obtained (92% yield).

R_f = 0.10 (PE/EtOAc 9:1); **^{1}H NMR** (400 MHz, d_6-benzene) δ 7.12-7.19 (m, 1 H, CH=C*H*CH_2), 5.86-5.96 (m, 1 H, COC*H*=CH), 3.50-3.54 (m, 1 H, CH_2C*H*OTBS), 3.40 (s, 3 H, COOC*H₃*), 2.71-2.77 (m, 1 H, C*H*$COOCH_3$), 2.46-2.54 (m, 1 H, CH_2C*H*COO), 2.08-2.23 (m, 2 H, CH=C*H₂*CHOTBS), 1.71-1.88 (m, 3 H, C*H₂*$CHCOOCH_3$, CH_2C*H₂*CH), 1.52-1.58 (m, 2 H, C*H₂*CH_2CH), 1.18-1.25 (m, 1 H, CH_2C*H₂*CH), 0.93 (s, 9 H, $OSi(CH_3)_2C(\mathit{CH_3})_3$), 0.00 (s, 3 H, $OSi(\mathit{CH_3})_2C(CH_3)_3$), -0.01 (s, 3 H, $OSi(\mathit{CH_3})_2C(CH_3)_3$); **^{13}C NMR** (100 MHz, d_6-benzene) δ 176.3 (q, 1 C, *C*OOH), 170.1 (q, 1 C, *C*$OOCH_3$), 148.0 (t, 1 C, CH=*C*HCH_2), 123.3 (t, 1 C, CO*C*H=CH), 73.6 (t, 1 C, *C*HOTBS), 51.0 (p, 1 C, COO*C*H_3), 48.3 (t, 1 C, CH_2CH_2*C*H), 44.5 (t, 1 C, *C*$HCOOCH_3$), 38.7 (s, 1 C, CH=CH*C*H_2), 31.8 (s, 1 C, *C*$H_2CHCOOCH_3$), 29.7 (s, 1 C, CH_2*C*H_2CH), 26.1 (s, 1 C, *C*H_2CH_2CH), 25.8 (p, 3 C, $OSi(CH_3)_2C(\mathit{C}H_3)_3$), 18.0 (q, 1 C, $OSi(CH_3)_2\mathit{C}(CH_3)_3$), -4.4 (s, 1 C, $OSi(\mathit{C}H_3)_2C(CH_3)_3$), -4.7 (s, 1 C, $OSi(\mathit{C}H_3)_2C(CH_3)_3$); **HRMS (ESI)** *m/z* calculated for $C_{18}H_{31}O_5Si$ $[M-H]^-$: 355.1941, found: 355.1967.

Methyl (1*R*,2*R*)-2-((*S*,*E*)-1-((*tert*-butyldimethylsilyl)oxy)-5-((2,5-dioxopyrrolidin-1-yl)oxy)-5-oxopent-3-en-1-yl)cyclopentane-1-carboxylate (19)

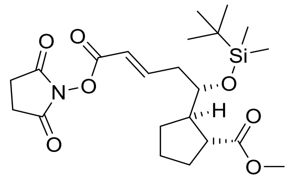

To a stirred solution of 155 mg (440 µmol, 1.0 equiv) of the precursor acid in 12 mL THF were added 50 mg (440 µmol, 1 equiv) *N*-hydroxysuccinimide and 89 mg (440 µmol, 1.0 equiv) *N,N'*-dicyclohexylcarbodiimide, and the mixture was stirred for 16 h at ambient temperature. Et_2O was added under vigorous stirring, and after filtration the resulting solid was three times washed with Et_2O. The solvent was removed under reduced pressure, and the crude product was purified by preparative HPLC (C18-$P_{[B]}$) (H_2O/MeCN 90:10 {5 min}, gradient H_2O/MeCN 90:10 → 0:100 {45 min}, H_2O/MeCN 0:100 {10 min}, 4 mL/min → 5 mL/min). 108 mg (240 µmol, t_R = 50.3 min) of the colorless oil **19** were obtained (54% yield).

^{1}H NMR (400 MHz, $CDCl_3$) δ 7.23-7.31 (m, 2 H, CH=C*H*CH_2), 6.04 (d, *J* = 16.0 Hz, 1 H, COC*H*=CH), 3.78-3.83 (m, 1 H, C*H*OTBS), 3.67 (s, 3 H, OC*H₃*), 2.85 (bs, 4 H, C*H₂*CON), 2.70-2.76 (m, 1 H, C*H*$COOCH_3$), 2.40-2.53 (m, 3 H, CH=CHC*H₂*, C*H*COOCH), 1.76-1.91 (m, 3 H, C*H₂*$CHCOOCH_3$, CH_2C*H₂*CH), 1.61-1.68 (m, 2 H, C*H₂*CH_2CH), 1.37-1.46 (m, 1 H, CH_2C*H₂*CH), 0.88 (s, 9 H, $OSi(CH_3)_2C(\mathit{CH_3})_3$), 0.06

(s, 6H, OSi(C*H*$_3$)$_2$C(CH$_3$)$_3$); ^{13}**C NMR** (400 MHz, CDCl$_3$) δ 177.0 (q, 2 C, CH$_2$*C*ON), 169.2 (q, 1 C, *C*OOCH$_3$), 161.1 (q, 1 C, CH$_2$*C*OON), 152.3 (t, 1 C, CH=*C*HCH$_2$), 117.5 (t, 1 C, CO*C*H=CH), 73.2(t, 1 C, *C*HOTBS), 51.7 (t, 1 C, CH$_2$CH$_2$*C*H), 48.2 (p, 1 C, O*C*H$_3$), 44.5 (t, 1 C, *C*HCOOCH$_3$), 39.3 (s, 1 C, CH=CH*C*H$_2$), 31.6 (s, 1 C, *C*H$_2$CHCOOCH$_3$), 29.7 (s, 1 C, CH$_2$*C*H$_2$CH), 26.0 (s, 1 C, *C*H$_2$CH$_2$CH), 25.8 (s, 2 C, 2 C, *C*H$_2$CON), 25.6 (p, 3 C, OSi(CH$_3$)$_2$C(*C*H$_3$)$_3$), 18.0 (q, 1 C, OSi(CH$_3$)$_2$*C*(CH$_3$)$_3$), -4.2 (p, 1 C, OSi(*C*H$_3$)$_2$C(CH$_3$)$_3$), -4.6 (p, 1 C, OSi(*C*H$_3$)$_2$C(CH$_3$)$_3$); **HRMS (ESI)** *m/z* calculated for $C_{22}H_{35}NO_7NaSi$ $[M+Na]^+$: 476.2081, found: 476.2085.

(*S,E*)-5-((*tert*-Butyldimethylsilyl)oxy)-5-((1*R*,2*R*)-2-(methoxycarbonyl)cyclopentyl)pent-2-enoyl-CoA thioester (20)

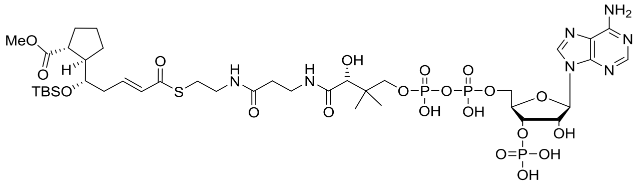

11 mg (15 µmol, 1 equiv) of coenzyme A trilithium salt were solved in 1.1 mL water. 10 mg (22 µmol, 1.5 equiv) of activated ester **19** in 2.2 mL THF were added. The pH was adjusted to 8.0 by the addition of 1 M $NaHPO_4$, and the mixture was stirred at 35 °C for 18 h. The reaction mixture was three times washed with 5 mL Et_2O. After the removal of water under reduced pressure, **20** was obtained as a colorless solid.

^{1}**H NMR** (400 MHz, D$_2$O) δ 8.41-8.52 (m, 1 H, NC*H*N), 8.08-8.22 (m, 1 H, NC*H*N), 6.77-6.85 (m, 1 H, CH=C*H*CH$_2$), 6.08-6.14 (m, 2 H, *H*COCH, COC*H*=CH), 4.52-4.56 (m, 1 H, HCOC*H*), 4.20 (bs, 2 H, C*H$_2$*OP), 3.95-3.99 (m, 1 H, POC*H$_2$*C(CH$_3$)$_2$), 3.75-3.84 (m, 1 H; POC*H$_2$*C(CH$_3$)$_2$), 3.60-3.66 (m, 3 H, OC*H$_3$*), 3.29-3.52 (m, 6 H, 2x NHC*H$_2$*, C*H$_2$*CHOTBS), 2.67-2.74 (m, 2 H, C*H$_2$*S), 2.34-2.46 (m, 4 H, C*H$_2$*CONH, C*H*OTBS, C*H*COOCH$_3$), 1.19-1.88 (m, 6 H, cyclopentane), 0.82 (2x s, 12 H, C*H$_3$*, OSi(CH$_3$)$_2$C(C*H$_3$*)$_3$), 0.68 (s, 3 H, C*H$_3$*), 0.01-0.07 (m, 6 H, OSi(C*H$_3$*)$_2$C(CH$_3$)$_3$); **MS (ESI)** *m/z* calculated for $C_{39}H_{67}N_7O_{20}P_3SSi$ $[M+H]^+$: 1106.3, found: 1106.3.

(*S*,*E*)-5-(Hydroxy)-5-((1*R*,2*R*)-2-(methoxycarbonyl)cyclopentyl)pent-2-enoyl-CoA thioester (21)

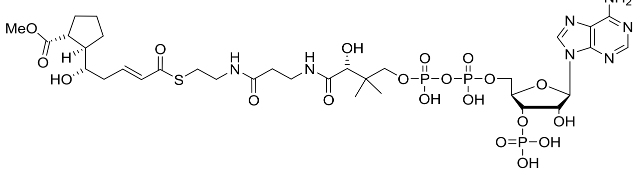

0.5 mg (0.45 µmol, 1.0 equiv) of CoA-ester **20** were dissolved in a mixture of THF/HCOOH/H_2O (6:3:1, 300 µL), and the resulting solution was stirred at ambient

temperature. The reaction was monitored by LC–MS. The results of the LC–MS (ESI) analysis after 48 h are shown in Figures S1–S4.

MS (ESI) *m/z* calculated for $C_{33}H_{53}N_7O_{20}P_3S$ $[M+H]^+$: 992.2, found: 992.2.

Methyl (1*R*,2*R*)-2-((1*S*,*E*)-1-((*tert*-butyldimethylsilyl)oxy)-5-hydroxy-6-methyl-7-oxo-7-(phenylthio)hept-3-en-1-yl)cyclopentane-1-carboxylate (17a and 17b)

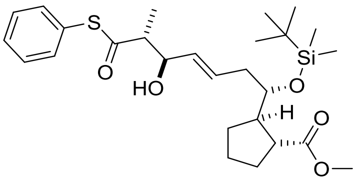

612.5 µL (0.61 mmol, 3.6 equiv, 1 M in hexane) chlorodicyclohexylborane were dissolved in 7 mL Et_2O and cooled to −78 °C. 80.5 µL (0.75 mmol, 4.4 equiv) dimethylethylamine and 70 mg (0.42 mmol, 2.5 equiv) thiophenolpropionate were added dropwise, and the reaction mixture was stirred for 2 h at 0 °C. The mixture was cooled to −78 °C, and 60 mg (0.17 mmol, 1 equiv) of unsaturated aldehyde **11** were added. The reaction was stirred for 1 h at −78 °C and then stored in a freezer at −20 °C for 18 h. 2 mL Methanol, 2 mL phosphate buffer (pH 7) and 2 mL H_2O_2 (35%) were added to the solution, and the mixture was stirred for 1 h at ambient temperature. The aqueous layer was three times extracted with CH_2Cl_2, and the combined organic layers were dried over $MgSO_4$. The solvent was removed in vacuo, and the crude product was purified by flash chromatography on silica gel (petroleum ether/EtOAc 100:1 then petroleum ether/EtOAc 50:1 and petroleum ether/EtOAc 20:1). After drying in vacuo, 50 mg (98.8 µmol) of a colorless oil, which consisted of a mixture of **17a** and **17b,** was obtained (57% yield).

$\mathbf{R_f}$ = 0.17 (PE/EtOAc 9:1); **^{1}H NMR** (400 MHz, $CDCl_3$) δ 7.42 (s, 5 H, C*H*(ar)), 5.68-5.77 (m, 1 H, CH_3C*H*=CH), 5.42-5.42 (m, 1 H, CH_3CH=C*H*), 4.26 (dd, J_1 = 7.5 Hz, J_2 = 7.5 Hz, 1 H, C*H*OH), 3.64-3.69 (m, 1 H, C*H*OTBS), 3.66 (s, 3 H, OCH_3), 2.87 (dq, J_1 = 7.5 Hz, J_2 = 7.0 Hz, 1 H, CH_3C*H*), 2.73-2.79 (m, 1 H, CH_2CH_2C*H*), 2.44-2.51 (m, 1 H, C*H*$COOCH_3$), 2.22-2.26 (m, 1 H, CH=CHCH_2), 1.83-1.92 (m, 1 H, CH_2CH$COOCH_3$), 1.71-1.82 (m, 1 H, CH_2CH$COOCH_3$, CH_2CH_2CH), 1.59-1.66 (m, 2 H, CH_2CH_2CH), 1.35-1.44 (m, 1 H, CH_2CH_2CH), 1.24 (d, *J* = 7.0 Hz, 3 H, CH_3CH), 0.88 (s, 9 H, $OSi(CH_3)_2C(CH_3)_3$), 0.06 (s, 3 H, $OSi(CH_3)_2C(CH_3)_3$), 0.05 (s, 3 H, $OSi(CH_3)_2C(CH_3)_3$); **^{13}C NMR** (100 MHz, $CDCl_3$) δ 201.3 (q, 1 C, S*C*O), 177.7 (q, 1 C, *C*$OOCH_3$), 134.4 (t, 2 C, Ar*C*), 132.3 (t, 1 C, *C*H=CHCH_2), 130.2 (t, 1 C, CH=*C*HCH_2), 129.4 (t, 2 C, Ar*C*), 129.2 (t, 1 C, Ar*C*), 127.5 (q, 1 C, Ar*C*), 75.1 (t, 1 C, *C*HOH), 74.4 (t, 1 C, *C*HOTBS), 53.7 (t, 1 C, CH_3*C*H), 51.7 (p, 1 C, COO*C*H_3), 47.2 (t, 1 C, *C*H$COOCH_3$), 44.4 (t, 1 C, CH_2CH_2*C*H), 38.9 (s, 1 C, CH=CH*C*H_2), 32.1 (s, 1 C, *C*H_2CH$COOCH_3$), 30.1 (s, 1 C, CH_2*C*H_2CH), 26.1 (s, 1 C, *C*H_2CH_2CH), 25.9 (p, 3 C, $OSi(CH_3)_2C(CH_3)_3$), 18.0 (q, 1 C, $OSi(CH_3)_2C(CH_3)_3$), 15.0 (p, 1 C, *C*H_3CH), -4.1

(p, 1 C, OSi(*C*H_3)$_2$C(CH_3)$_3$), -4.7 (p, 1 C, OSi(CH_3)$_2$*C*(CH_3)$_3$); **HRMS (ESI)** *m/z* calculated for $C_{27}H_{42}O_5SSiNa$ $[M+Na]^+$: 529.2420, found: 529.2422.

Methyl (1*R*,2*R*)-2-((5*S*,*E*)-9-hydroxy-2,2,3,3,10-pentamethyl-11,16-dioxo-4-oxa-12-thia-15-aza-3-silaheptadec-7-en-5-yl)cyclopentane-1-carboxylate (18a and 18b)

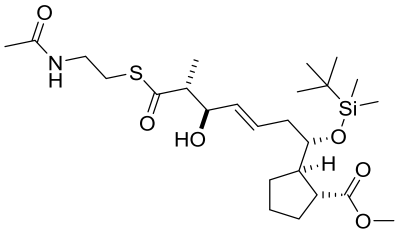

10 mg (20 µmol, 1.0 equiv) of the mixture of aldol products **17a** and **17b**, 16 µL (91 µmol, 4.6 equiv) DIPEA and 20 µL (188 µmol, 9.4 equiv) HSNAc were dissolved in 1 mL DMF and stirred for 18 h at room temperature. 5 mL Brine were added, and the mixture was stirred for a further 5 min. The mixture was three times extracted with Et_2O, the organic layers were washed with brine and dried over $MgSO_4$. The solvent was removed in vacuo, and the crude product was purified by HPLC (HPLC (C18-ISIS) (H_2O/MeOH 80:20 {10 min}, gradient H_2O/MeOH 80:20 → 45:55 {30 min}, gradient H_2O/MeOH 45:55 → 20:80 {25 min}, gradient H_2O/MeOH 20:80 → 10:90 {30 min}, gradient H_2O/MeOH 10:90 → 0:100 {5 min}, 2.5 mL/min → 3.5 mL/min). After drying in vacuo, 7.2 mg (14 µmol, t_R = 72.5 min) of an colorless oil, which consisted of a mixture of **18a** and **18b**, were obtained (70% yield).

^{1}H NMR (500 MHz, $CDCl_3$) δ 5.93-6.01 (m, 1 H, N*H*), 5.69-5.79 (m, 1 H, CH=C*H*CH_2), 5.37-5.47 (m, 1 H, C*H*=CHCH_2), 4.22-4.26 (m, 1 H, C*H*OH), 3.67-3.71 (m, 1 H, C*H*OTBS), 3.68 (d, *J* = 5.6 Hz, 1 H, OMe), 3.42-3.56 (m, 2 H, NHC*H*$_2$), 3.04-3.14 (m, 2 H, C*H*$_2$S), 2.75-2.81 (m, 2 H, CHCH_3, C*H*COOH), 2.45-2.51 (m, 1 H, C*H*), 2.20-2.30 (m, 1 H, OH), 1.98 (s, 3 H, CHC*H*$_3$), 1.88-1.95 (m, 1 H, 1x C*H*$_2$CHCOOH), 1.73-1.82 (m, 2 H, 1x C*H*$_2$CHCOOH, 1x C*H*$_2$CH), 1.63-1.68 (m, 2 H, C*H*$_2$$CH_2$CH), 1.37-1.45 (m, 1 H, 1x C*H*$_2$CH), 1.16 (d, *J* = 7.0 Hz, 3 H, C*H*$_3$CO), 0.90 (s, 9 H, OSi(CH_3)$_2$C(C*H*$_3$)$_3$), 0.08 (dd, J_1 = 2.8 Hz, J_2 = 1.7 Hz, 6 H, OSi(C*H*$_3$)$_2$C(CH_3)$_3$); **^{13}C NMR** (125 MHz, $CDCl_3$) δ 203.4 (q, 1 C, S*C*O), 177.8 (q, 1 C, *C*OOCH_3), 170.4 (q, 1 C, CH_3*C*ONH), 132.4 (t, 1 C, *C*H=CHCH_2), 130.5 (t, 1 C, CH=*C*HCH_2), 75.4 (t, 1 C, *C*HOTBS), 74.2 (t, 1 C, *C*HOH), 54.1 (t, 1 C, *C*HCH_3), 51.6 (p, 1 C, OMe), 47.2 (t, 1 C, *C*HCOOH), 44.5 (t, 1 C, *C*H), 39.4 (s, 1 C, NH*C*H_2), 38.8 (s, 1 C, CH=CH*C*H_2), 32.1 (s, 1 C, *C*H_2CHCOOH), 30.2 (s, 1 C, CH_2*C*H_2CH), 26.1 (s, 1 C, *C*H_2S), 25.8 (p, 3 C, OSi(CH_3)$_2$C(*C*H_3)$_3$), 23.3 (s, 1 C, *C*$H_2$$CH_2$CH), 18.0 (q, 1 C, OSi($CH_3$)$_2$*C*($CH_3$)$_3$), 15.0 (p, 1 C, CH*C*$H_3$), -4.1 (p, 2 C, OSi(*C*$H_3$)$_2$C($CH_3$)$_3$); **HRMS (ESI)** *m/z* calculated for $C_{25}H_{46}NO_6SSi$ $[M+H]^+$: 516.2815, found: 516.2820.

(1*R*,2*R*)-2-((1*S*,*E*)-7-((2-Acetamidoethyl)thio)-1,5-dihydroxy-6-methyl-7-oxohept-3-en-1-yl)cyclopentane-1-carboxylic acid (5a and 5b)

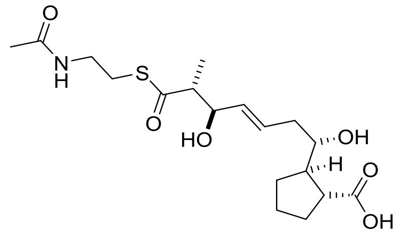

30 mg (58 µmol, 1.0 equiv) of the *N*-acetylcysteamine thioesters **18a** and **18b** were dissolved in a mixture of THF/HCOOH/H_2O (6:3:1, 2 mL) and stirred for 48 h at ambient temperature. 5 mL saturated $NaHCO_3$ solution were added, and the aqueous layer was three times extracted with EtOAc. The organic layers were dried over $MgSO_4$, and the solvent was removed under reduced pressure. Free TBS side products were removed by azeotropic distillation with toluene, and the crude product was used in the next step without further purification.

The crude material was dissolved in 2 mL phosphate buffer (pH 8), and 100 units of porcine liver esterase were added. It was stirred for 5 d at room temperature during which the course of the reaction was monitored by LC–MS. The reaction mixture was three times extracted with EtOAc as well as EtOAc/iPrOH (3:1, 1.5 mL), and the solvent was removed in vacuo. The crude product was purified by preparative HPLC (C18-ISIS) (H_2O/MeCN 80:20 {10 min}, gradient H_2O/MeCN 80:20 → 0:100 {80 min}, H_2O/MeCN = 0:100 {10 min}, 15 mL/min). After drying in vacuo, 18 mg (46.5 µmol, t_R = 40.0 min) of an colorless oil, which consisted of a mixture of **5a** and **5b**, were obtained with full conversion and an overall yield of 81%.

^{1}H NMR (400 MHz, CD_2Cl_2) δ 6.07 (bs, 1 H, NH), 5.72-5.81 (m, 1 H, CH=C*H*CH_2), 5.65 (dd, J_1 = 15.4 Hz, J_2 = 6.5 Hz, 1 H, C*H*=CHCH_2), 4.24-4.31 (m, 1 H, C*H*OH), 3.53-3.60 (m, 1 H, CH_2C*H*OH), 3.38-3.48 (m, 2 H, NHC*H*$_2$), 3.04-3.11 (m, 1 H, 1x C*H*$_2$S), 2.93-3.01 (m, 1 H, 1x C*H*$_2$S), 2.84-2.90 (m, 1 H, C*H*CH_3), 2.75-2.81 (m, 1 H, C*H*COOH), 2.44-2.51 (m, 1 H, 1x CH=CHC*H*$_2$), 2.05-2.24 (m, 3 H, 1x CH=CHC*H*$_2$, C*H*, 1x C*H*$_2$CH), 1.97 (s, 3 H, C*H*$_3$CO), 1.80-1.92 (m, 2 H, C*H*$_2$CHCOOH), 1.58-1.74 (m, 2 H, C*H*$_2$$CH_2$CH), 1.26-1.34 (m, 1 H, CH_2C*H*$_2$CH), 1.22 (d, *J* = 7.2 Hz, 3 H, CHC*H*$_3$); **^{13}C NMR** (100 MHz, CD_2Cl_2) δ 203.0 (q, 1 C, S*C*O), 176.6 (q, 1 C, *C*OOH), 171.2 (q, 1 C, CH_3*C*ONH) , 134.2 (t, 1 C, *C*H=CHCH_2), 128.4 (t, 1 C, CH=*C*HCH_2), 75.9 (t, 1 C, CH_2*C*HOH), 74.8 (t, 1 C, *C*HOH), 54.2 (t, 1 C, *C*HCH_3), 48.9 (t, 1 C, *C*H), 48.2 (t, 1 C, *C*HCOOH), 39.1 (s, 1 C, NH*C*H_2), 38.9 (s, 1 C, CH=CH*C*H_2), 30.6 (s, 1 C, *C*H_2CHCOOH), 29.3 (s, 1 C, CH_2*C*H_2CH), 28.8 (s, 1 C, CH_2*C*H_2S), 25.7 (s, 1 C, *C*$H_2$$CH_2$CH), 22.9 (p, 1 C, *C*$H_3$CO), 14.0 (p, 1 C, CH*C*$H_3$); **HRMS (ESI)** *m/z* calculated for $C_{18}H_{29}NO_6SNa$ $[M+Na]^+$: 410.1613, found: 410.1653.

Methyl (1*R*,2*R*)-2-((*S*,3*E*,5*E*)-1-((*tert*-butyldimethylsilyl)oxy)-7-methoxy-6-methyl-7-oxohepta-3,5-dien-1-yl)cyclopentane-1-carboxylate (10a)

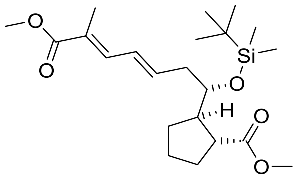

15 mg (67 µmol, 1.1 equiv) methyl 2-(diethoxyphosphoryl)propanoate were dissolved in 0.5 mL THF and cooled to 0 °C. 3 mg (73 µmol, 1.2 equiv, 60% in mineral oil) NaH was added, and the reaction mixture was stirred for 1 h at 0 °C. 25 mg (61 µmol, 1 equiv) of unsaturated aldehyde **11** were added and the reaction was stirred for a further 3 h at 0 °C. The reaction was quenched with 3 mL water and 10 mL saturated $NaHCO_3$ solution, and the solvent was reduced in vacuo. The aqueous layer was three times extracted with Et_2O, and the organic layers were dried over $MgSO_4$. The solvent was removed under reduced pressure, and the crude product was purified by flash chromatography on silica gel (petroleum ether/EtOAc 50:1 then petroleum ether/EtOAc 20:1 and petroleum ether/EtOAc 10:1). After drying in vacuo, 4.8 mg (12 µmoL) of the colorless oil **10a** were obtained (18% yield).

A higher yielding alternative was the Wittig olefination with phosphorane **28**:

8.8 mg (28 µmol, 1.0 equiv) of olefin **12** were subjected to olefin cross metathesis with crotonaldehyde and second generation Grubbs catalyst and purified as described previously. The resulting unsaturated aldehyde **11** was solved in 2 mL CH_2Cl_2 and 11 mg (31 µmol, 1.1 equiv) of **26** were added. The resulting solution was stirred at 50 °C for 21 h. The solvent was removed under reduced pressure, and the crude product was purified by flash chromatography on C18-reversed phase silica gel ($MeCN/H_2O$ 2:3). After drying in vacuo, 7.4 mg (18 µmol) of the colorless oil **10a** were obtained (64% yield).

R_f = 0.4 (PE/EtOAc 9:1); **$[\alpha]_D^{23}$** = +5.8 (*c* = 0.7, CH_2Cl_2); **^{1}H NMR** (500 MHz, benzene-d_6) δ 7.46 (d, *J* = 11.4 Hz, 1 H, C=C*H*CH), 6.37 (dd, J_1 = 12.5 Hz, J_2 = 13.9 Hz, 1 H, C*H*=CHCH$_2$), 5.90-5.96 (m, 1 H, CH=C*H*CH$_2$), 3.53-3.57 (m, 1 H, C*H*OTBS), 3.44 (s, 3 H, OC*H$_3$*), 3.37 (s, 3 H, OC*H$_3$*), 2.81-2.87 (m, 1 H, C*H*COOCH$_3$), 2.58-2.64 (m, 1 H, CH$_2$CH$_2$C*H*), 2.29-2.38 (m, 1 H, C*H$_2$*CHOTBS), 2.21-2.26 (m, 1 H, C*H$_2$*CHOTBS), 1.99 (s, 3 H, C*H$_3$*CCH), 1.78-1.88 (m, 2 H, C*H$_2$*CHCOOCH$_3$), 1.62-1.70 (m, 1 H, 1x CH$_2$C*H$_2$*CH), 1.55-1.62 (m, 1 H, 1x C*H$_2$*CH$_2$CH), 1.47-1.54 (m, 1 H, C*H$_2$*CH$_2$CH), 1.27-1.32 (m, 1 H, CH$_2$C*H$_2$*CH), 0.94 (s, 9 H, OSi(CH$_3$)$_2$C(C*H$_3$*)$_3$), 0.04 (s, 3 H, OSi(C*H$_3$*)$_2$C(CH$_3$)$_3$), 0.01 (s, 3 H, OSi(C*H$_3$*)$_2$C(CH$_3$)$_3$); **^{13}C NMR** (125 MHz, benzene-d_6) δ 176.8 (q, 1 C, CH*C*OOCH$_3$), 168.5 (q, 1 C, C*C*OOCH$_3$), 138.6 (t, 1 C, CH=*C*HCH$_2$), 138.5 (t, 1 C, C*C*H=CH), 128.9 (t, 1 C, *C*H=CHCH$_2$), 126.2 (q, 1 C, CH$_3$*C*CH), 74.7 (t, 1 C, *C*HOTBS), 51.4 (p, 1 C, O*C*H$_3$), 51.3 (p, 1 C, O*C*H$_3$), 48.2 (t, 1 C, *C*HCOOCH$_3$), 44.8 (t, 1 C, CH$_2$CH$_2$*C*H), 40.3 (s, 1 C, *C*H$_2$CHOTBS), 32.3 (s, 1 C, *C*H$_2$CHCOOCH$_3$), 30.3 (s, 1

C, CH_2CH_2CH), 26.5 (s, 1 C, CH_2CH_2CH), 26.1 (p, 3 C, $OSi(CH_3)_2C(CH_3)_3$), 18.3 (q, 1 C, $OSi(CH_3)_2C(CH_3)_3$), 12.9 (p, 1 C, CH_3CCH), -3.9 (s, 1 C, $OSi(CH_3)_2C(CH_3)_3$), -4.4 (s, 1 C, $OSi(CH_3)_2C(CH_3)_3$); **HRMS (ESI)** *m/z* calculated for $C_{22}H_{39}O_5Si$ $[M+H]^+$: 411.2567, found: 411.2563.

Methyl (1*R*,2*R*)-2-((*S*,3*E*,5*E*)-1-hydroxy-7-methoxy-6-methyl-7-oxohepta-3,5-dien-1-yl)cyclopentane-1-carboxylate (7a)

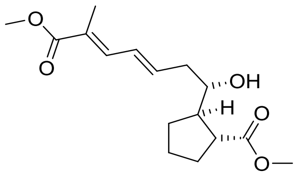

7.4 mg (18 µmol, 1 equiv) of the TBS-protected precursor **10a** were dissolved in 2 mL THF/HCOOH/H_2O 6:3:1 and stirred at room temperature. Monitoring of the reaction by LC–MS showed full conversion after two days, and the reaction was quenched with a saturated $NaHCO_3$ solution. After extraction with EtOAc, the combined organic layers were dried over $MgSO_4$, and the solvent removed under reduced pressure. The crude material was purified by flash chromatography on C_{18}-reversed phase silica gel (H_2O then MeCN/H_2O 1:4). After drying in vacuo, 3.3 mg (11.1 µmol) of the colorless oil **7a** were obtained (62% yield).

R_f = 0.21 (PE/EtOAc 9:1); **$[\alpha]_D^{23}$** = -1.0 (*c* = 0.1, CH_2Cl_2); **^{1}H NMR** (400 MHz, $CDCl_3$) δ 7.17 (d, *J* = 11.4 Hz, 1 H, C=C*H*=CH), 6.43 (dt, J_1 = 14.9 Hz, J_2 = 11.3 Hz, 1 H, C*H*=$CHCH_2$), 6.13 (dt, J_1 = 14.9 Hz, J_2 = 7.5 Hz, 1 H, CH=C*H*CH_2), 3.75 (s, 3 H, OCH_3), 3.70 (s, 3 H, OCH_3), 3.13-3.17 (dt, J_1 = 3.1 Hz, J_2 = 8.1 Hz, 1 H, C*H*OH), 2.71 (dt, J_1 = 8.2 Hz, J_2 = 8.2 Hz, 1 H, C*H*$COOCH_3$), 2.44-2.54 (m, 1 H, $CHCH_2$CHOTBS), 2.22-2.34 (m, 2 H, 1x $CHCH_2$CHOTBS, 1x CHC*H*CHOH), 1.93 (s, 3 H, CH_3CCH), 1.91-2.00 (m, 1 H, 1x CH_2CHCOOH), 1.81-1.91 (m, 2 H, 1x CH_2CHCOOH, 1x CH_2CH), 1.64-1.74 (m, 2 H, CH_2CH_2CH), 1.22-1.38 (m, 1 H, 1x CH_2CH); **^{13}C NMR** (100 MHz, $CDCl_3$) δ 177.7 (q, 1 C, CH*C*$OOCH_3$), 169.2 (q, 1 C, C*C*$OOCH_3$), 138.4 (t, 2 C, CH=*C*HCH_2, C*C*H=CH), 128.9 (t, 1 C, *C*H=$CHCH_2$), 126.0 (q, 1 C, CH_3*C*CH), 75.3 (t, 1 C, *C*HOH), 52.1 (p, 1 C, OCH_3), 51.9 (p, 1 C, OCH_3), 49.7 (t, 1 C, *C*$HCOOCH_3$), 47.8 (t, 1 C, CH_2CH_2*C*H), 40.4 (s, 1 C, CH*C*H_2CHOTBS), 31.1 (s, 1 C, *C*$H_2CHCOOCH_3$), 30.0 (s, 1 C, CH_2*C*H_2CH), 25.5 (s, 1 C, CH_2*C*H_2CH_2), 12.8 (p, 1 C, *C*H_3CCH); **HRMS (ESI)** *m/z* calculated for $C_{16}H_{24}O_5Na$ $[M+Na]^+$: 319.1521, found: 319.1514.

Methyl (1*R*,2*R*)-2-((*S*,3*E*,5*Z*)-1-((*tert*-butyldimethylsilyl)oxy)-7-methoxy-6-methyl-7-oxohepta-3,5-dien-1-yl)cyclopentane-1-carboxylate (10b)

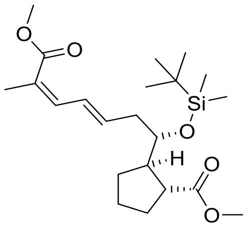

190 mg (720 µmol, 12 equiv) 18-crown-6 and 50 mg (360 µmol, 6 equiv) K_2CO_3 were suspended in 2 mL toluene and stirred for 1 h at room temperature. The suspension was cooled to −20 °C and 20 mg (6 µmol, 1 equiv) **27** were added. 25 mg (60 µmol, 1.0 equiv) of unsaturated aldehyde **11** were added and the reaction was stirred for a further 5 h at 0 °C. After the addition of 10 mL brine, the aqueous layer was three times extracted with Et_2O, and the organic layers were dried over $MgSO_4$. The solvent was removed under reduced pressure, and the crude product was purified by flash chromatography on silica gel (petroleum ether/EtOAc 50:1 then petroleum ether/EtOAc 20:1 and petroleum ether/EtOAc 10:1). After drying in vacuo, 11 mg (28 µL) of the colorless oil **10b** were obtained (47% yield).

R_f = 0.6 (PE/EtOAc 9:1); **$[\alpha]_D^{23}$** = +14.4 (*c* = 0.8, CH_2Cl_2); **^{1}H NMR** (500 MHz, benzene-d_6) δ 7.64 (dd, J_1 = 12.5 Hz, J_2 = 13.8 Hz, 1 H, C*H*=CHCH$_2$), 6.27 (d, *J* = 10.9 Hz, 1 H, C=C*H*=CH), 5.93-6.01 (m, 1 H, CH=C*H*CH$_2$), 3.57-3.61 (m, 1 H, C*H*OTBS), 3.44 (s, 3 H, OC*H$_3$*), 3.40 (s, 3 H, OC*H$_3$*), 2.80-2.86 (m, 1 H, C*H*COOCH$_3$), 2.66-2.73 (m, 1 H, CH$_2$CH$_2$C*H*), 2.28-2.43 (m, 2 H, C*H$_2$*CHOTBS), 1.87 (s, 3 H, C*H$_3$*CCH), 1.77-1.86 (m, 2 H, C*H$_2$*CHCOOCH$_3$), 1.64-1.73 (m, 1 H, CH$_2$C*H$_2$*CH), 1.54-1.62 (m, 1 H, C*H$_2$*CH$_2$CH), 1.45-1.53 (m, 1 H, C*H$_2$*CH$_2$CH), 1.20-1.29 (m, 1 H, CH$_2$C*H$_2$*CH), 0.98 (s, 9 H, OSi(CH$_3$)$_2$C(C*H$_3$*)$_3$), 0.06 (s, 3 H, OSi(C*H$_3$*)$_2$C(CH$_3$)$_3$), 0.05 (s, 3 H, OSi(C*H$_3$*)$_2$C(CH$_3$)$_3$); **^{13}C NMR** (125 MHz, benzene-d_6) δ 176.9 (q, 1 C, CH*C*OOCH$_3$), 167.5 (q, 1 C, C*C*OOCH$_3$), 141.3 (t, 1 C, CH=*C*HCH$_2$), 137.5 (t, 1 C, C*C*H=CH), 130.9 (t, 1 C, *C*H=CHCH$_2$), 124.7 (q, 1 C, CH$_3$*C*CH), 75.2 (t, 1 C, *C*HOTBS), 51.3 (p, 1 C, O*C*H$_3$), 50.9 (p, 1 C, O*C*H$_3$), 48.2 (t, 1 C, *C*HCOOCH$_3$), 45.3 (t, 1 C, CH$_2$CH$_2$*C*H), 39.7 (s, 1 C, *C*H$_2$CHOTBS), 32.3 (s, 1 C, *C*H$_2$CHCOOCH$_3$), 30.2 (s, 1 C, CH$_2$*C*H$_2$CH), 26.4 (s, 1 C, *C*H$_2$CH$_2$CH), 26.2 (p, 3 C, OSi(CH$_3$)$_2$C(*C*H$_3$)$_3$), 20.9 (q, 1 C, OSi(CH$_3$)$_2$*C*(CH$_3$)$_3$), 18.3 (p, 1 C, *C*H$_3$CCH), -3.9 (s, 1 C, OSi(*C*H$_3$)$_2$C(CH$_3$)$_3$), -4.5 (s, 1 C, OSi(*C*H$_3$)$_2$C(CH$_3$)$_3$); **HRMS (ESI)** *m/z* calculated for $C_{22}H_{38}O_5NaSi$ $[M+Na]^+$: 433.2386, found: 433.2387.

(Methyl (1*R*,2*R*)-2-((*S*,3*E*,5*Z*)-1-hydroxy-7-methoxy-6-methyl-7-oxohepta-3,5-dien-1-yl)cyclopentane-1-carboxylate (7b)

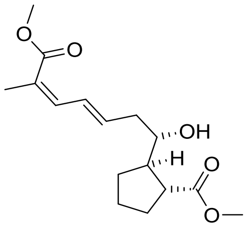

2 mg (7 µmol, 1 equiv) of the TBS-protected precursor **10b** were dissolved in 1 mL of THF/HCOOH/H_2O 6:3:1 and stirred at room temperature. Monitoring of the reaction by LC–MS showed full conversion after two days, and the reaction was quenched by the addition of a saturated $NaHCO_3$ solution. After extraction with EtOAc, the combined organic layers were dried over $MgSO_4$, and the solvent removed under reduced pressure. The crude material was purified by HPLC (C18-$P_{[B]}$) (H_2O/MeOH 80:20 {5 min}, gradient H_2O/MeOH 80:20 → 0:100 {95 min}, 4 mL/min → 5 mL/min). After drying in vacuo, 1 mg (4 µmol, t_R = 72.3 min) of the colorless oil **7b** was obtained (57% yield).

$[\alpha]_D^{23}$ = +1.0 (*c* = 0.1, CH_2Cl_2); **^{1}H NMR** (500 MHz, benzene-d_6) δ 7.14-7.20 (m, 1 H, C*H*=CHCH$_2$), 6.42 (d, *J* = 11.2 Hz, 1 H, C=C*H*=CH), 5.94-6.01 (m, 1 H, CH=C*H*CH$_2$), 3.76 (s, 3 H, OC*H$_3$*), 3.69 (s, 3 H, OC*H$_3$*), 3.64-3.67 (m, 1 H, C*H*OH), 3.48-3.54 (m, 2 H, C*H$_2$*CHOTBS), 2.66-2.72 (m, 1 H, C*H*COOCH$_3$), 1.95 (s, 3 H, C*H$_3$*CCH), 1.17-1.89 (m, 6 H, 3x C*H$_2$*(cyclopentane)); **HRMS (ESI)** *m/z* calculated for $C_{16}H_{24}O_5Na$ $[M+Na]^+$: 319.1521, found: 319.1516.

***S*-(2-Acetamidoethyl) 2-bromopropanethioate (23)**

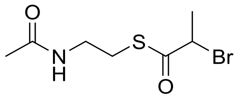

1.27 g (8.30 mmol, 1.1 equiv) of acid **22**, 810 µL (7.60 mmol, 1.0 equiv) of HSNAc and 92 mg (800 µmol, 1.0 equiv) of DMAP were solved in 20 mL CH_2Cl_2 and cooled to 0 °C. 1.70 g (8.30 mmol, 1.1 equiv) EDC·HCl were added in several portions. Stirring at 0 °C was continued for 1 h and for 16 h at room temperature. The organic layer was washed with saturated $NaHCO_3$, water and NaCl, and dried over $MgSO_4$. The solvent was removed under reduced pressure. 1.64 g (6.45 mmol) of the pure pale-yellow oil **23** were obtained and directly subjected to the next step without further purification (78% yield).

R_f = 0.3 (PE/EtOAc 1:2 + 10% MeOH); **^{1}H NMR** (400 MHz, $CDCl_3$) δ 6.07 (bs, 1 H, N*H*), 4.50 and 4.47 (q, *J* = 6.9 Hz, 2 H, C*H*Br), 3.37-3.50 (m, 2 H, C*H$_2$*NH), 3.01-3.12 (m, 2 H, C*H$_2$*S), 1.96 (bs, 3 H, C*H$_3$*CO), 1.82 and 1.68 (d, *J* = 6.9 Hz, 1 H, C*H$_3$*CHBr); **^{13}C NMR** (100 MHz, $CDCl_3$) δ 196.6 (q, 1 C, *C*(=O)S), 170.7 (q, 1 C, *C*(=O)N), 47.8 (t,

1 C, *C*HBr), 39.0 (s, 1 C, *C*H_2NH), 29.3 (s, 1 C, *C*H_2S), 23.1 (p, 1 C, *C*H_3CO), 21.9 (p, 1 C, *C*H_3CHBr); **HRMS (ESI)** *m/z* calculated for $C_7H_{12}BrNOS$ $[M+H]^+$: 253.9850, found: 253.9852.

S-(2-Acetamidoethyl) 2-(triphenyl-λ^5-phosphanylidene)propanethioate (24)

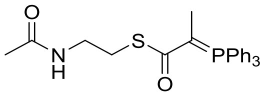

200 mg (791 µmol, 1.1 equiv) of **23** were suspended in 1 mL water and 188 mg (719 µmol, 1.0 equiv) triphenylphosphine were added. The solution was stirred at 70 °C for 11 h. The suspension was cooled to room temperature. 64 mg (1.60 mmol, 2.0 equiv) NaOH in 2 mL water were added and it was stirred at room temperature for 5 min. The aqueous layer was three times extracted with CH_2Cl_2, and the combined organic layers were dried over $MgSO_4$. After removal of the solvent, recrystallization from toluene, and drying in vacuo, 201 mg (462 µmol) of a crystal white solid **24** (64% yield) were obtained.

^{1}H NMR (400 MHz, $CDCl_3$) δ 7.44-7.62 (m, 15 H, H(ar)), 3.28-3.37 (m, 2 H, C*H_2*NH), 2.97-3.05 (m, 2 H, C*H_2*S), 1.68 (d, *J* = 6.9 Hz, 3 H, C*H_3*CP), 1.52 (s, 3 H, C*H_3*CO); **^{13}C NMR** (100 MHz, $CDCl_3$) δ 180.5, 180.4 (q, 1 C, *C*(=O)S), 170.6 (q, 1 C, *C*(=O)N), 133.7, 133.6 (t, 6 C, *C*H(ar)), 132.3 (t, 3 C, *C*H(ar)), 129.1, 129.0 (t, 6 C, *C*H(ar)), 126.4, 125.5 (q, 3 C, *C*P(ar)), 53.7, 52.6 (q, 1 C, *C*=P), 43.4 (s, 1 C, *C*H_2NH), 26.9 (s, 1 C, *C*H_2S), 23.3 (p, 1 C, *C*H_3CO), 13.2, 13.1 (p, 1 C, *C*H_3CP); **HRMS (ESI)** *m/z* calculated for $C_{25}H_{26}NO_2PS$ $[M+H]^+$: 436.1500, found: 436.1501.

(1*R*,2*R*)-methyl 2-((*S*,7*E*,9*E*)-2,2,3,3,10-pentamethyl-11,16-dioxo-4-oxa-12-thia-15-aza-3-silaheptadeca-7,9-dien-5-yl)cyclopentanecarboxylate (9a)

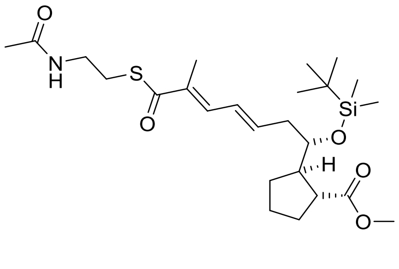

5 mg (14.2 µmol, 1 equiv) of olefin **12** were subjected to metathesis reaction as described for the synthesis of unsaturated aldehyde **11**. The product from this reaction was dissolved in 1 mL of CH_2Cl_2 and 7.2 mg (16.5 µmol, 1.2 equiv) of **24** were added. The solution was stirred at 50 °C overnight, and the solvent was removed under reduced pressure. The crude material was purified by flash chromatography on silica gel (petroleum ether/EtOAc 1:1). After drying in vacuo, 6.4 mg (12.5 µmol) of the colorless oil **9a** were obtained (88% yield over two steps).

R_f = 0.11 (PE/EtOAc 9:1); **$[\alpha]_D^{23}$** = +8.0 (*c* = 0.6, CH_2Cl_2); **^{1}H NMR** (500 MHz, Acetone-d_6) δ 7.17 (d, *J* = 11.1 Hz, 1 H, C=C*H*CH), 6.53 (dd, J_1 = 11.1 Hz, J_2 = 15.0 Hz, 1 H, C*H*=CHCH$_2$), 6.24-6.31 (m, 1 H,CH=C*H*CH$_2$), 3.86 (q, *J* = 5.7 Hz, 1 H, C*H*OTBS), 3.62 (s, 3 H, OMe), 3.32 (q, *J* = 6.5 Hz, 2 H, NHC*H$_2$*), 3.01-3.07 (m, 2 H, C*H$_2$*S), 2.75-2.85 (m, 1 H, C*H*COOMe), 2.43-2.53 (m, 3 H, CH, CH=CHC*H$_2$*), 1.96 (s, 3 H, C*H$_3$*CO), 1.86 (s, 3 H, CHC*H$_3$*), 1.82-1.86 (m, 2 H, C*H$_2$*CHCOOH), 1.68-1.82 (m, 1 H, 1x CH$_2$C*H$_2$*CH), 1.55-1.67 (m, 2 H, C*H$_2$*CH$_2$CH), 1.42-1.52 (m, 1H, 1x CH$_2$C*H$_2$*CH), 0.90 (s, 9 H, OSi(CH$_3$)$_2$C(C*H$_3$*)$_3$), 0.11 (2 s, 6 H, OSi(C*H$_3$*)$_2$C(CH$_3$)$_3$); **^{13}C NMR** (125 MHz, Acetone-d_6) δ 192.9 (1 C, q, S*C*O), 177.4 (1 C, q, *C*OOCH$_3$), 170.1 (1 C, q, CH$_3$*C*ONH), 141.4 (1 C, t, CH=*C*HCH$_2$), 138.0 (1 C, t, *C*HCH=CH), 134.0 (1 C, q, CH$_3$*C*) 128.9 (1 C, t, *C*H=CHCH$_2$), 75.2 (1 C, t, CH$_2$*C*HOTBS), 51.8 (1 C, p, COO*C*H$_3$), 48.7 (1 C, t, *C*H), 45.5 (1 C, t, *C*HCOOCH$_3$), 40.5 (1 C, s, *C*H$_2$CHOTBS), 39.7 (1 C, s, NH*C*H$_2$), 32.6 (1 C, s, *C*H$_2$CHCOOCH$_3$), 30.6 (1 C, s, *C*H$_2$S), 29.2 (1 C, s, CH$_2$*C*H$_2$CH), 26.7 (1 C, s, *C*H$_2$CH$_2$CH), 26.3 (3 C, p, OSi(CH$_3$)$_2$C(*C*H$_3$)$_3$), 22.8 (1 C, p, *C*H$_3$C), 18.6 (1 C, q, OSi(CH$_3$)$_2$*C*(CH$_3$)$_3$), 12.7 (1 C, p, *C*H$_3$CONH), -3.9 (1 C, p, OSi(*C*H$_3$)$_2$C(CH$_3$)$_3$), -4.5 (1 C, p, OSi(*C*H$_3$)$_2$C(CH$_3$)$_3$); **HRMS (ESI)** *m/z* calculated for $C_{25}H_{44}NO_5SSi$ $[M+H]^+$: 498.2709, found: 498.2709.

(1*R*,2*R*)-Methyl 2-((*S*,3*E*,5*E*)-7-((2-acetamidoethyl)thio)-1-hydroxy-6-methyl-7-oxohepta-3,5-dien-1-yl)cyclopentanecarboxylate

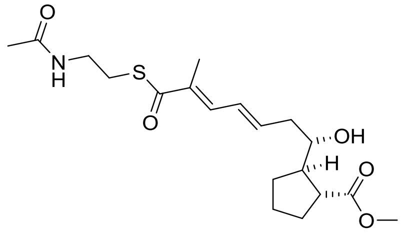

3.2 mg (6.4 µmol, 1 equiv) of **9a** were dissolved in 2 mL of THF/HCOOH/H_2O 6:3:1 and stirred at room temperature. After two days, the reaction was quenched by the addition of saturated $NaHCO_3$ solution. After extraction with EtOAc, the combined organic layers were dried over $MgSO_4$, and the solvent was removed under reduced pressure. The crude product, which was obtained as an colorless oil was analyzed by ^{1}H NMR spectroscopy and directly subjected to esterase-catalyzed deprotection.

The crude material from another entry was purified by HPLC for full spectroscopic characterisation (t_R = 30 min, C18-P) (H_2O/MeOH 90:10 {5 min}, gradient H_2O/MeOH 90:10 → 45:55 {45 min}, gradient H_2O/MeOH 45:55 → 0:100 {30 min}, H_2O/MeOH 0:100 {20 min}, 2.25 mL/min).

$[\alpha]_D^{23}$ = -1.0 (*c* = 0.1, CH_2Cl_2); **^{1}H NMR** (500 MHz, acetone-d_6) δ 7.26 (bs, 1 H, N*H*), 7.16 (d, *J* = 11.7 Hz, 1 H, C=C*H*CH), 6.54 (dd, J_1 = 13.7 Hz, J_2 = 11.66 Hz, 1 H, C*H*=CHCH$_2$), 6.29-6.37 (m, 1 H, CH=C*H*CH$_2$), 3.61 (s, 3 H, OMe), 3.57-3.63 (m, 1 H,C*H*OH), 3.29-3.36 (m, 2 H, C*H$_2$*NH), 3.02-3.07 (m, 2 H, C*H$_2$*S), 2.75-2.78 (m, 1 H,

C*H*$COOCH_3$), 2.42-2.52 (m, 1 H, 1x CH=CHC*H*$_2$), 2.29-2.39 (m, 2 H, 1x CH=CHC*H*$_2$, C*H*CHOH), 1.95 (s, 3 H, C*H*$_3$CO), 1.87-1.93 (m, 1 H, 1x C*H*$_2$$CHCOOCH_3$), 1.85 (s, 3 H, C*H*$_3$C=CH), 1.70-1.80 (m, 2 H, 1x C*H*$_2$$CHCOOCH_3$, 1x CH_2C*H*$_2$CH), 1.61-1.68 (m, 2 H, C*H*$_2$$CH_2$CH), 1.41-1.50 (m, 1 H, 1x CH_2C*H*$_2$CH); **^{13}C NMR** (125 MHz, acetone-d_6) 192.9 (1 C, q, S*C*O), 177.7 (1 C, q, *C*$OOCH_3$), 170.0 (1 C, q, *C*ONH), 142.7 (1 C, t, CH=*C*HCH_2), 138.3 (1 C, t, C*C*H=CH), 133.8 (1 C, q, *C*CH=CH), 128.4 (1 C, t, CH*C*H=CH), 74.5 (1 C, t, *C*HOH), 51.7 (1 C, p, COO*C*H_3), 50.2 (1 C, t, *C*HCHOH), 46.7 (1 C, t, *C*$HCOOCH_3$), 41.1 (1 C, s, CH=CH*C*H_2), 39.8 (1 C, s, *C*H_2NH), 32.2 (1 C, s, *C*$H_2$$CHCOOCH_3$), 30.5 (1 C, s, CH_2*C*H_2CH), 29.1 (1 C, s, *C*H_2S), 26.4 (1 C, s, *C*$H_2$$CH_2$CH), 22.9 (1 C, p, *C*$H_3$C), 12.7 (1 C, p, *C*$H_3$CO); **HRMS (ESI)** *m/z* calculated for $C_{19}H_{30}NO_5S$ $[M+H]^+$: 384.1845, found: 384.1846.

(1*R*,2*R*)-2-((*S*,3*E*,5*E*)-7-((2-Acetamidoethyl)thio)-1-hydroxy-6-methyl-7-oxohepta-3,5-dien-1-yl)cyclopentane-1-carboxylic acid (6a)

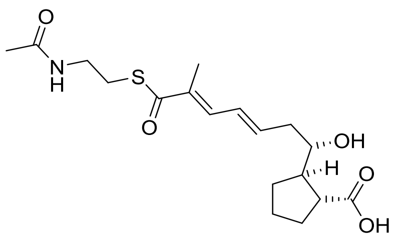

The crude material from the TBS deprotection was dissolved in 1 mL phosphate buffer (pH 8), and 100 units of porcine liver esterase were added. The mixture was stirred for 3 d at an ambient temperature during which the course of the reaction was monitored by LC-MS. After the conversion was complete, the crude material was purified by flash chromatography on C_{18}-reversed phase silica gel (H_2O then MeCN/H_2O 1:4). After drying in vacuo, 1.6 mg (4.3 µmol) of the colorless oil **6a** were obtained (67% yield over two steps).

^{1}H NMR (400 MHz, $CDCl_3$) δ 7.17 (d, J = 11.1 Hz, 1 H, C=C*H*CH), 6.48 (dd, J_1 = 11.1 Hz, J_2 = 15.0 Hz, 1 H, CHC*H*=CH), 6.17-6.27 (m, 1 H, CH=C*H*CH_2), 5.92-6.06 (m, 1 H, N*H*), 4.68 (s, 1 H, O*H*), 3.58-3.67 (m, 1 H,C*H*OH), 3.46 (dt, 2 H, J_1 = 6.4 Hz, J_2 = 5.9 Hz, C*H*$_2$NH), 3.09 (t, 2 H, J = 6.3 Hz, C*H*$_2$S), 2.77 (dt, 1 H, J_1 = 6.8 Hz, J_2 = 7.9 Hz, C*H*$COOCH_3$), 2.54-2.64 (m, 1 H, 1x CH=CHC*H*$_2$), 2.04-2.38 (m, 3 H, 1x CH=CHC*H*$_2$, C*H*CHOH, 1x C*H*$_2$$CHCOOCH_3$), 1.95-2.02 (2x s, 6 H, C*H*$_3$CO, CH=CC*H*$_3$), 1.80-1.94 (m, 2 H, 1x C*H*$_2$$CHCOOCH_3$, 1x CH_2C*H*$_2$CH), 1.61-1.73 (m, 2 H, C*H*$_2$$CH_2$CH), 1.27-1.33 (m, 1 H, 1x CH_2C*H*$_2$CH); **^{13}C NMR** (100 MHz, $CDCl_3$) 193.9 (1 C, q, S*C*O), 178.1 (1 C, q, *C*OOH), 170.7 (1 C, q, *C*ONH), 139.5 (1 C, t, CH=*C*HCH_2), 137.3 (1 C, t, C*C*H=CH), 133.9 (1 C, q, *C*CH=CH), 129.1 (1 C, t, CH*C*H=CH), 75.9 (1 C, t, *C*HOH), 49.2 (1 C, t, *C*HCHOH), 48.3 (1 C, t, *C*HCOOH), 40.5 (1 C, s, CH=CH*C*H_2), 40.0 (1 C, s, *C*H_2NH), 30.7 (1 C, s, CH_2*C*H_2CH), 29.9 (1 C, s, *C*H_2CHCOOH), 28.7 (1 C, s, *C*H_2S), 25.8 (1 C, s, *C*$H_2$$CH_2$CH), 23.4 (1 C, p,

CH_3C), 12.9 (1 C, p, *C*H_3CO); **HRMS (ESI)** *m/z* calculated for $C_{18}H_{26}NO_5S$ $[M-H]^-$: 368.1532, found: 368.1523.

Figures

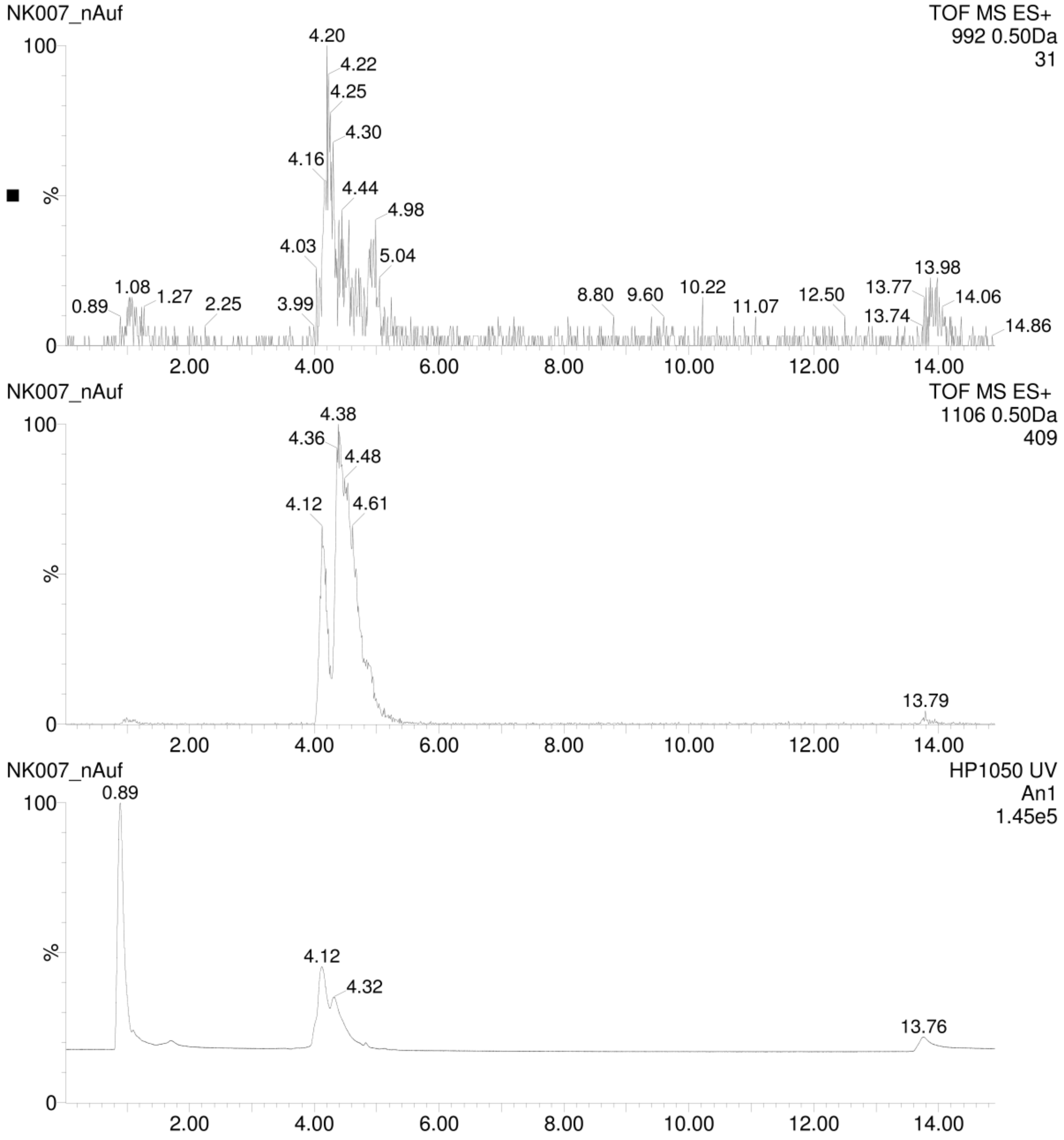

Figure S1: HPLC-MS analysis of the crude product **20** from the CoA thioesterification. The product shows an intense UV signal at 4.12 min; $[M+H]^+$ (**20**) = 1106.3, $[M+H]^+$ (**21**) = 992.2.

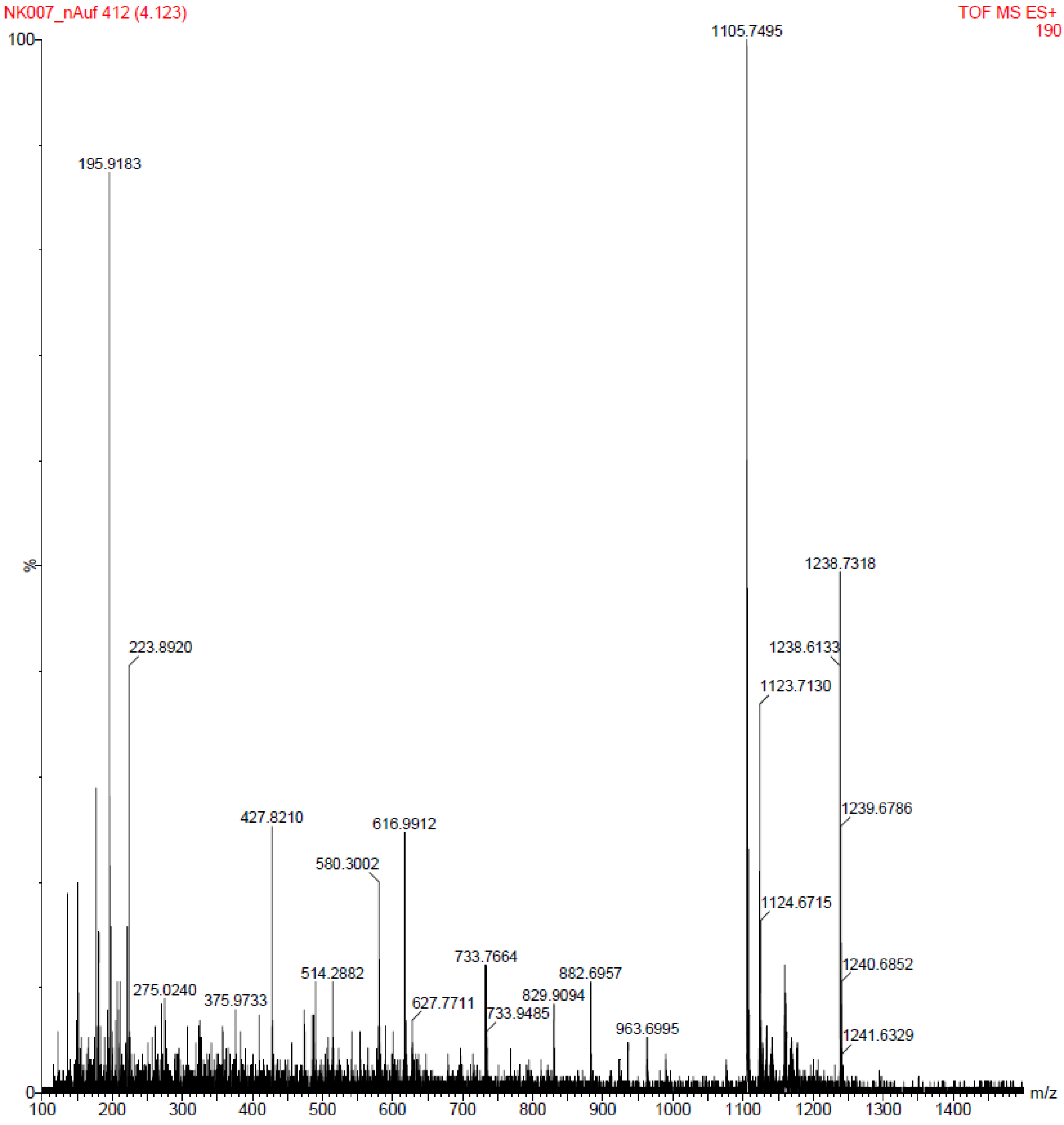

Figure S2: ESIMS analysis of **19** from the CoA thioesterification. $[M+H]^+$ (**20**) = 1106.3, $[M+H]^+$ (**21**) = 992.2, $[M+H]^+$ (CoASH) = 768.1, $[M+H]^+$ (**19**) = 454.2.

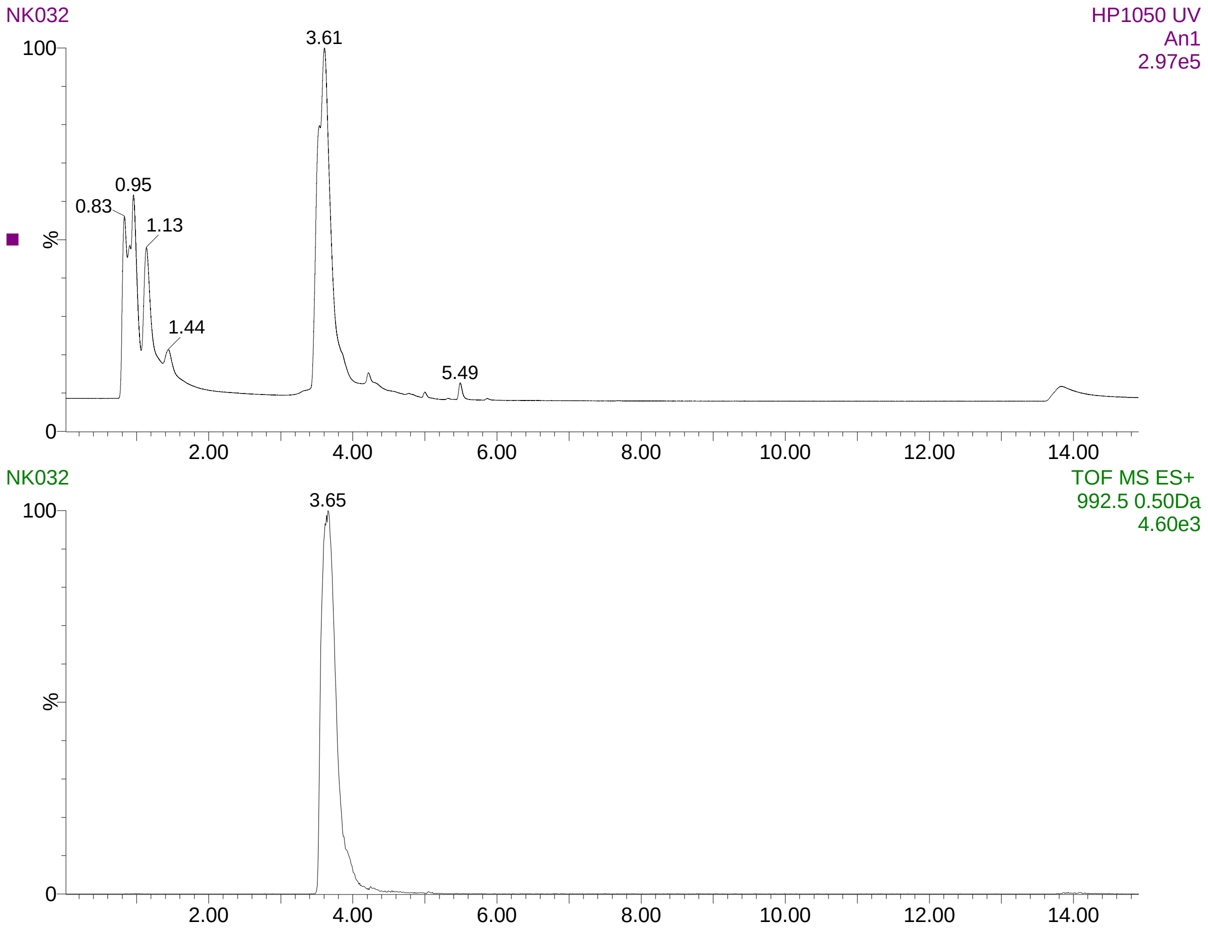

Figure S3: HPLC–MS analysis of the crude product from the acidic TBS-deprotection of **20**. The product shows an intense UV signal at 3.65 min, the starting material at 4.12 min is completely consumed; $[M+H]^+$ (**21**) = 992.2.

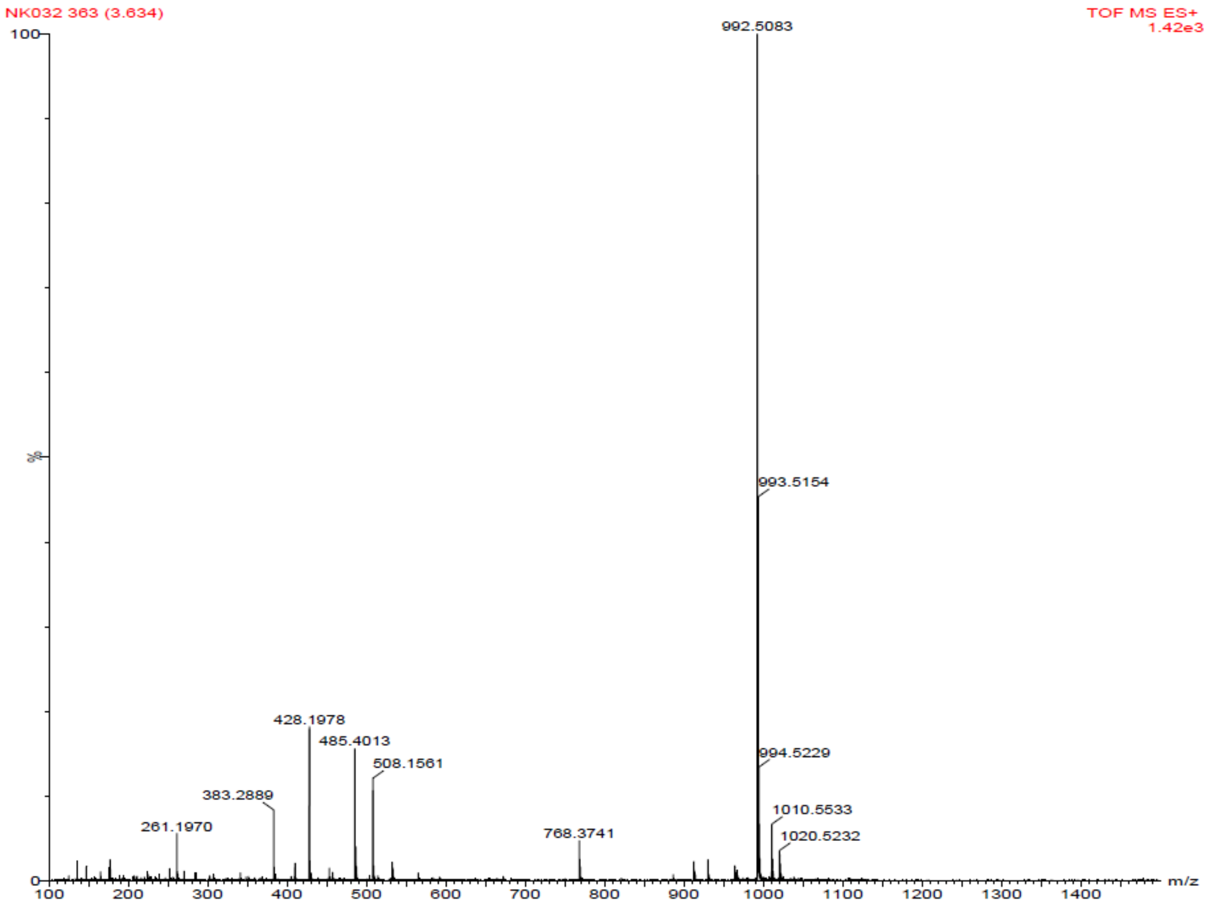

Figure S4: ESIMS analysis of the acidic TBS-deprotection of **20**. $[M+H]^+$ (**20**) = 1106.3, $[M+H]^+$ (**21**) = 992.2, $[M+H]^+$ (CoASH) = 768.1, $[M+H]^+$ (**19**) = 454.2.

References supporting information

1. H. E. Gottlieb, V. Kotlyar, A. Nudelman, *J. Org. Chem.* **1997**, *62*, 7512-7515.
2. Nagamitsu, T.; Takano, D.; Marumoto, K.; Fukuda, T.; Furuya, K.; Otoguro, K.; Takeda, K.; Kuwajima, I.; Harigaya, Y.; Omura, S. *J. Org. Chem.,* **2007,** *72*, 2744-2756.
