# Supplementary material for: Synthesis of complex intermediates for the study of a dehydratase from borrelidin biosynthesis
Source: Beilstein J Org Chem. 2014 Mar 11;10:634–40. doi: 10.3762/bjoc.10.55 (PMC3999764; doi:10.3762/bjoc.10.55)

Supporting information

for

Synthesis of complex intermediates for the study of a dehydratase from borrelidin biosynthesis

Frank Hahn*[,§1,2], Nadine Kandziora[§1], Steffen Friedrich[1] and Peter F. Leadlay[2]

Address: [1]Institut für Organische Chemie und Biomolekulares Wirkstoffzentrum, Leibniz Universität Hannover, Schneiderberg 1B, 30167 Hannover, Germany and [2]Department of Biochemistry, University of Cambridge, 80 Tennis Court Road, Cambridge CB2 1GA, United Kingdom

Email: Frank Hahn - frank.hahn@oci.uni-hannover.de

*Corresponding author

[§]These authors contributed equally

NMR spectra

CDCl$_3$, 200 MHz

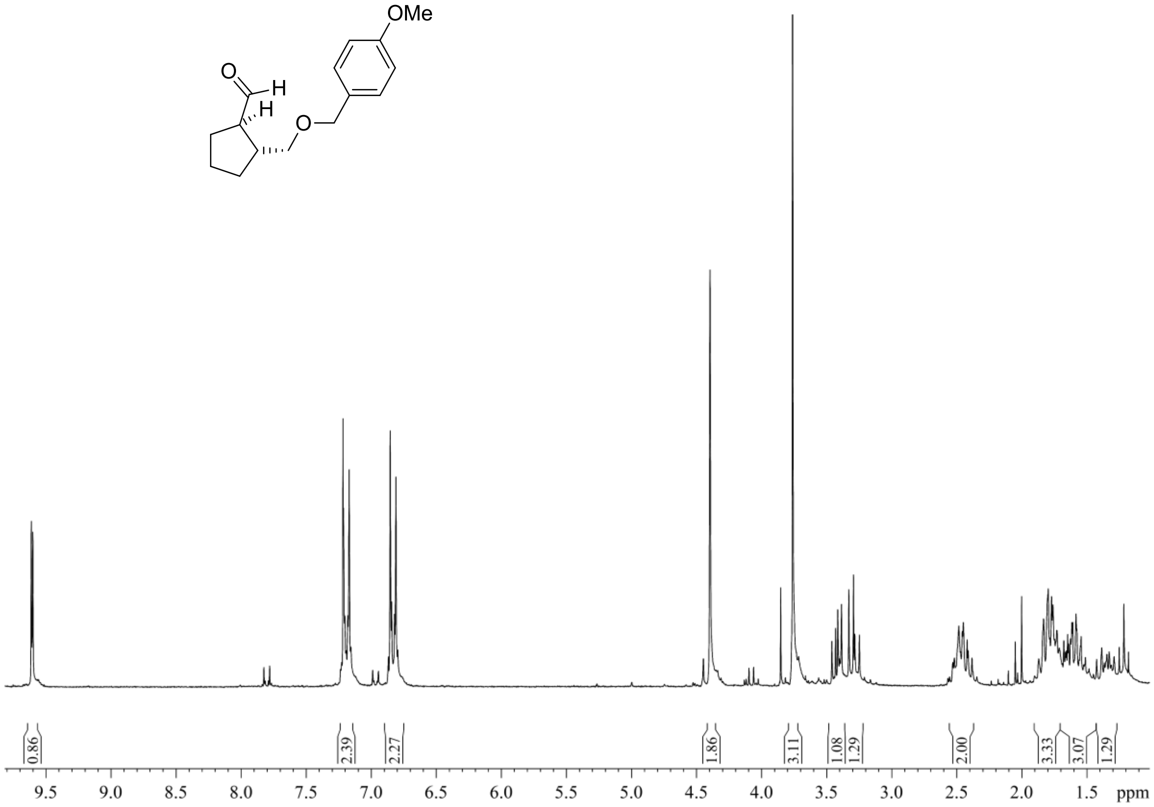

CDCl$_3$, 200 MHz

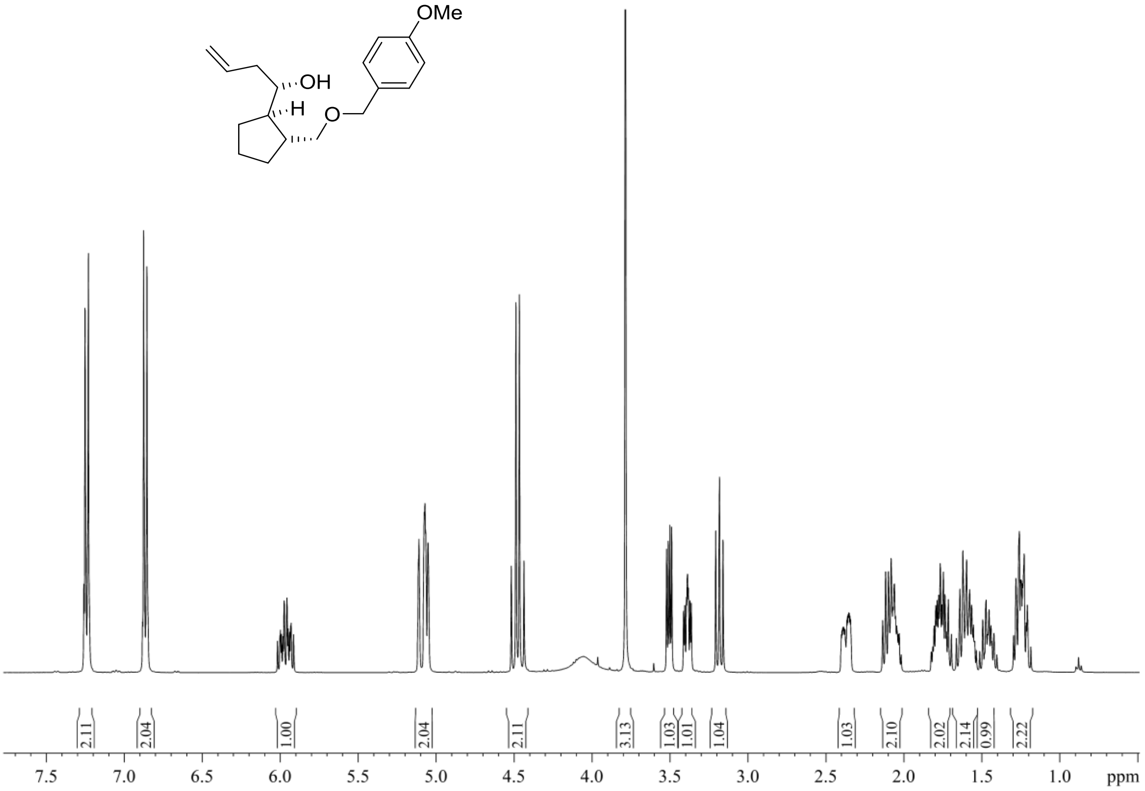

CDCl$_3$, 400 MHz

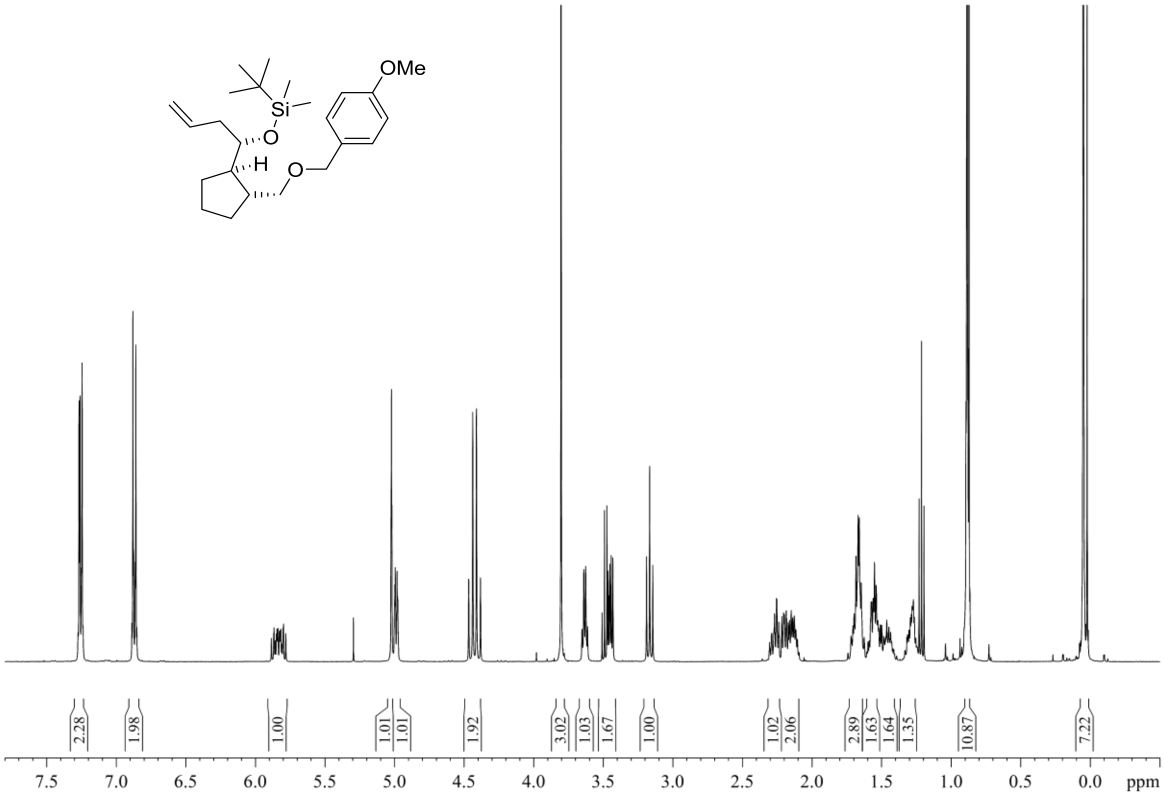

CDCl$_3$, 200 MHz

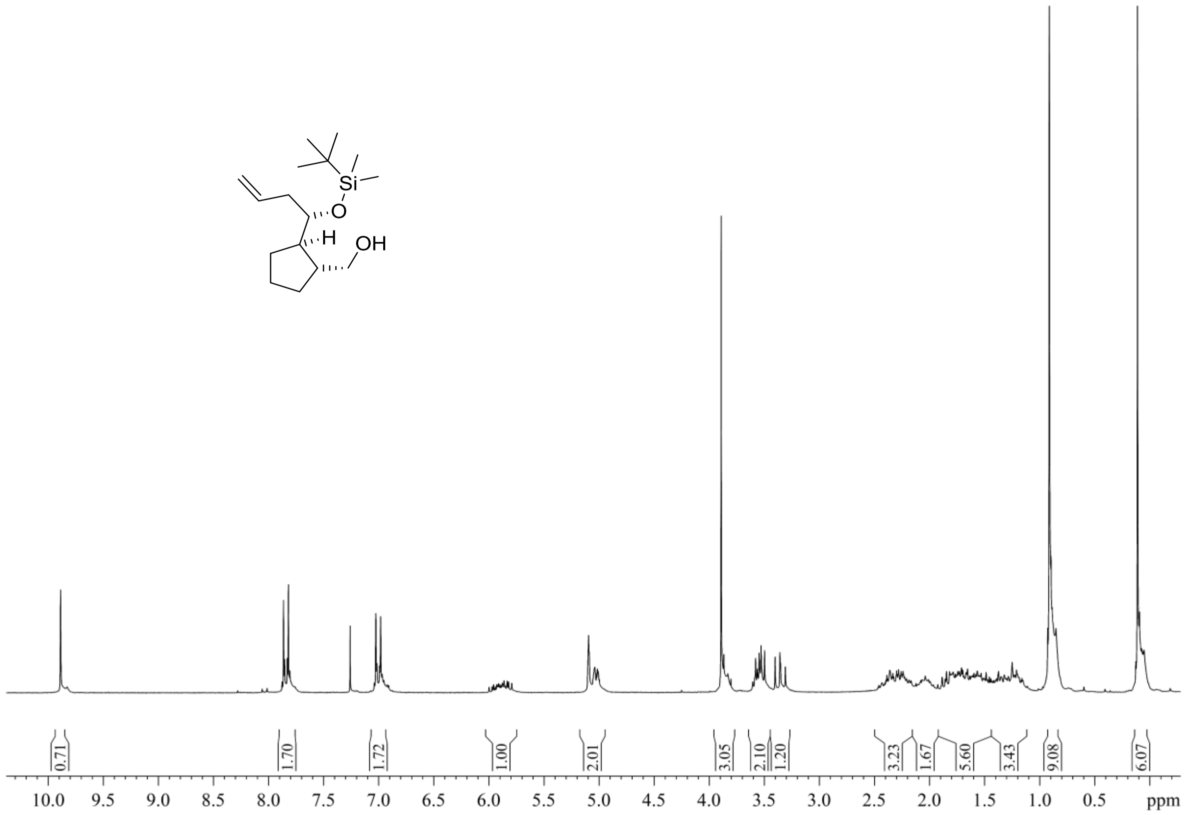

CDCl$_3$, 200 MHz

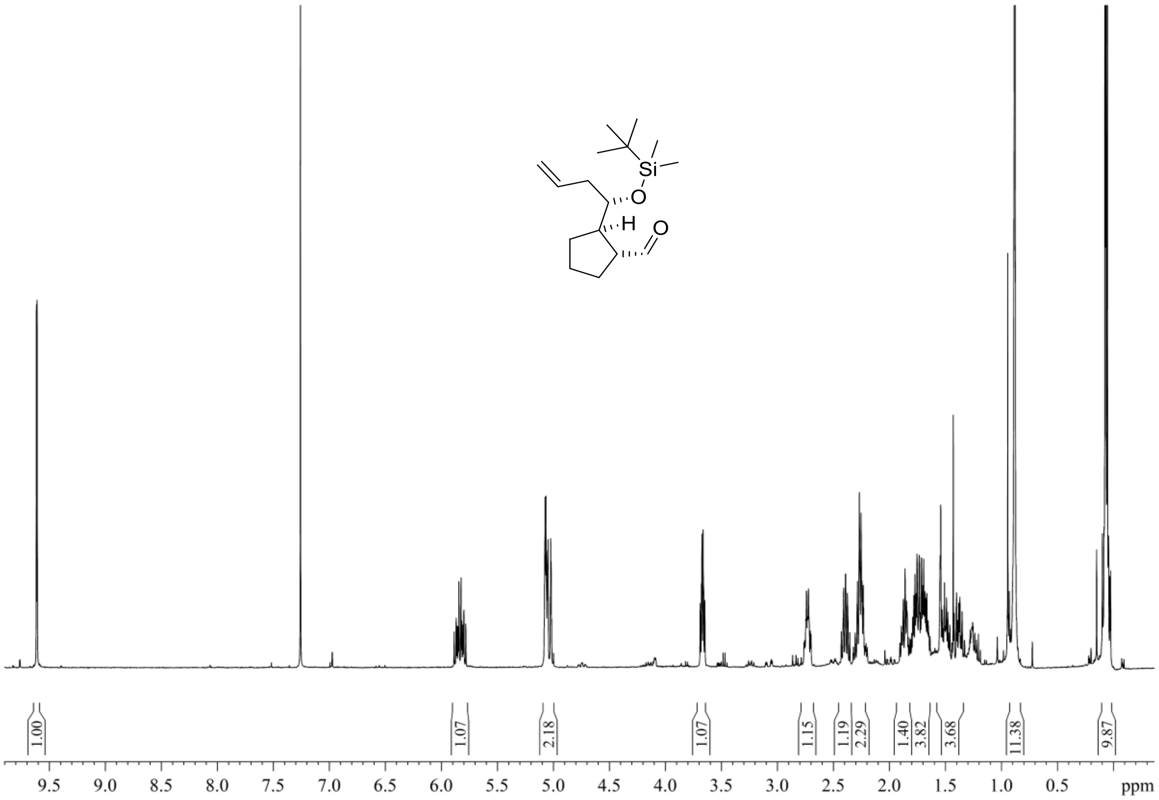

CDCl$_3$, 100 MHz

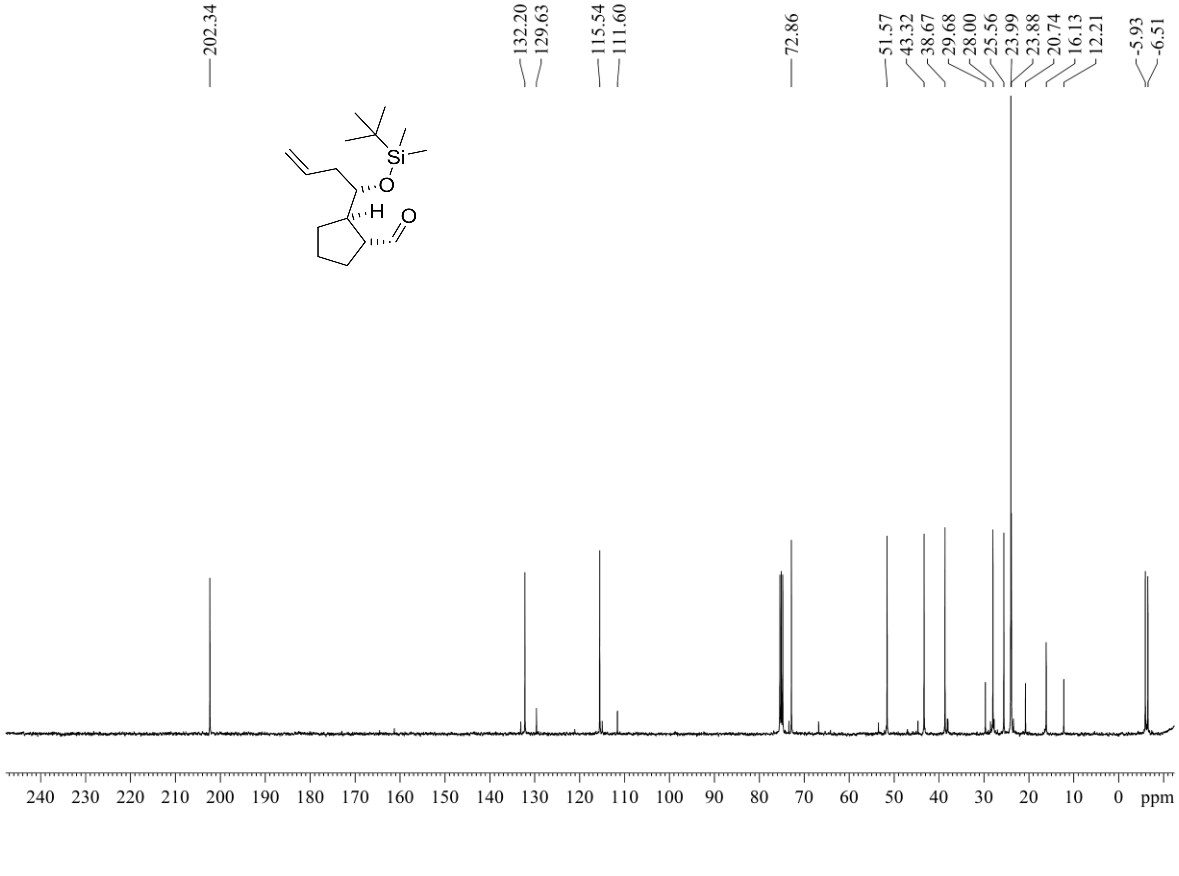

MeOD, 400 MHz

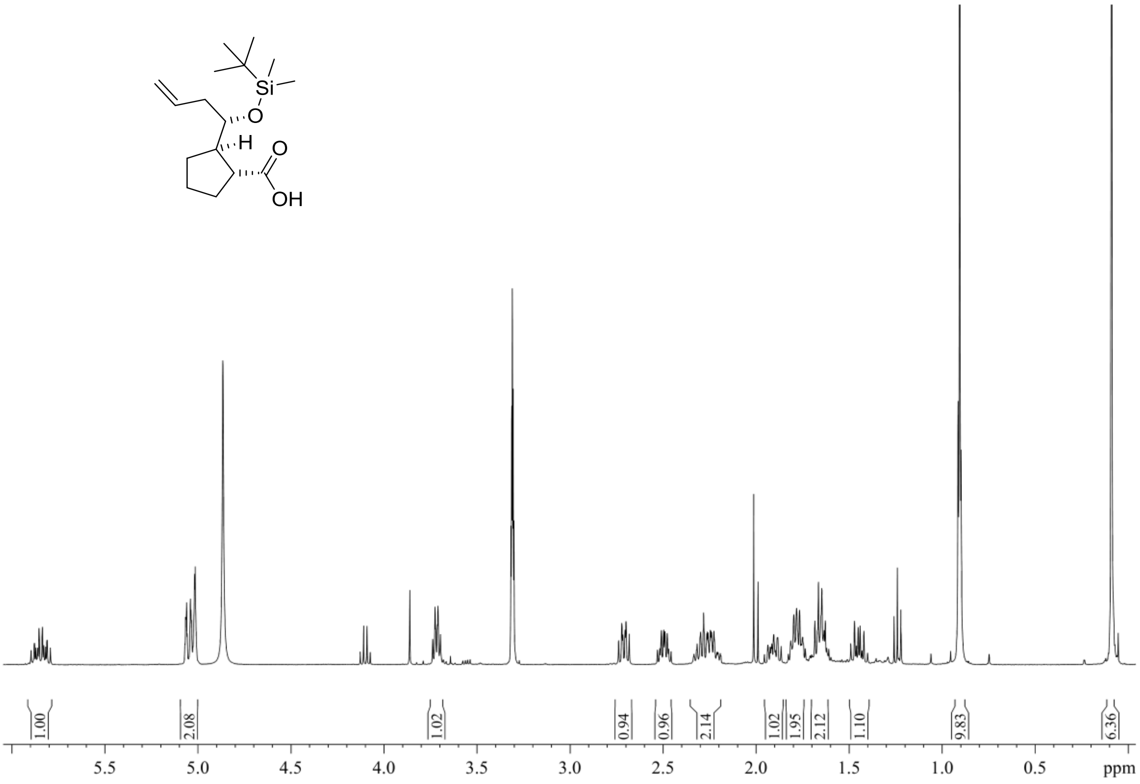

MeOD, 100 MHz

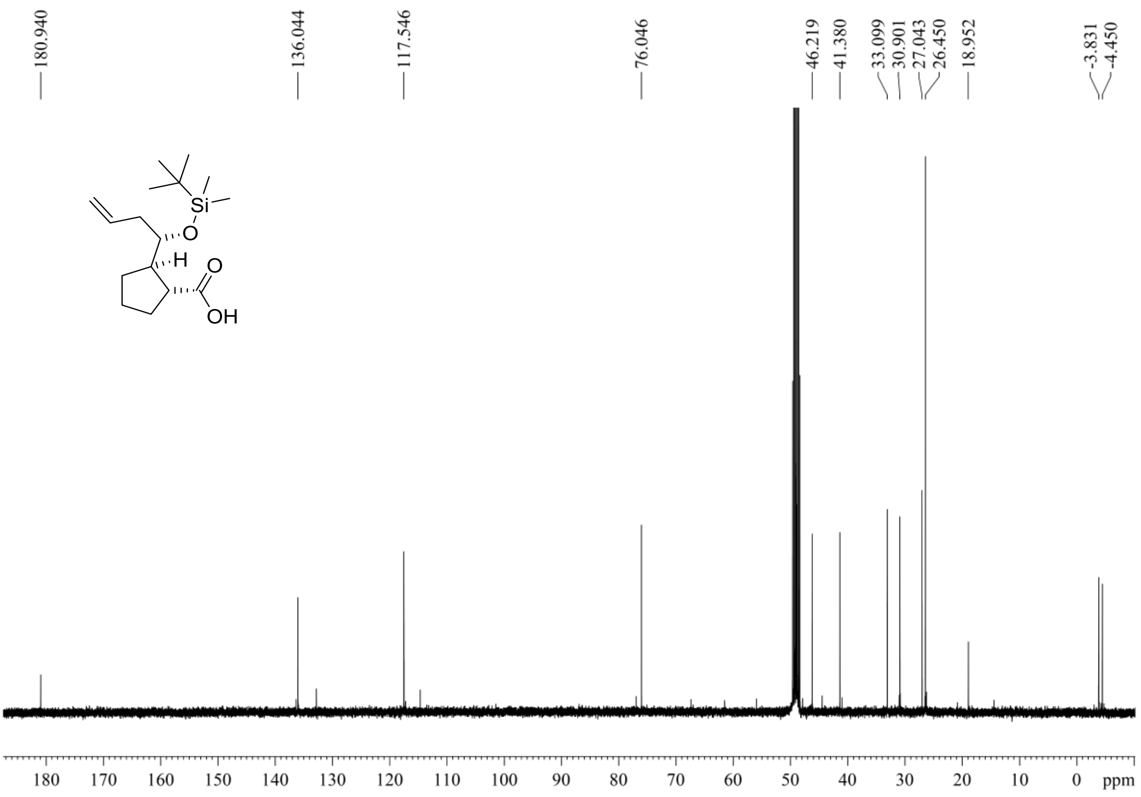

CDCl$_3$, 400 MHz

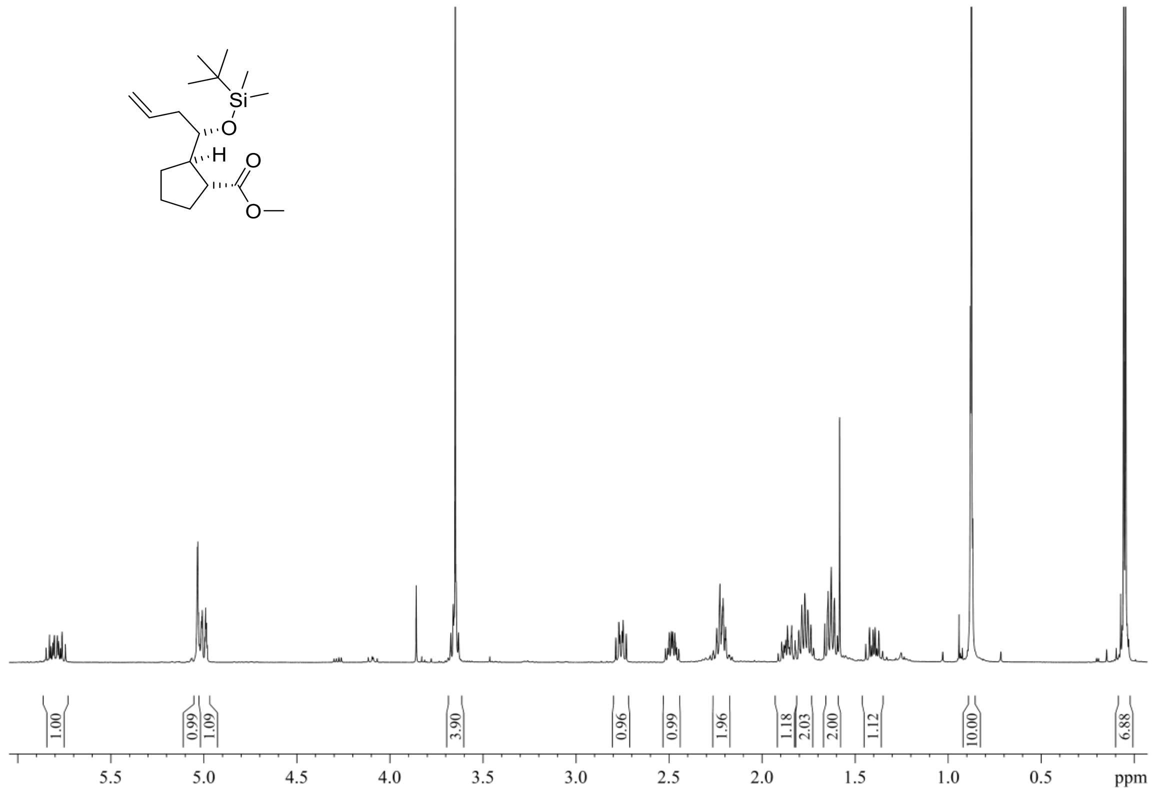

CDCl$_3$, 100 MHz

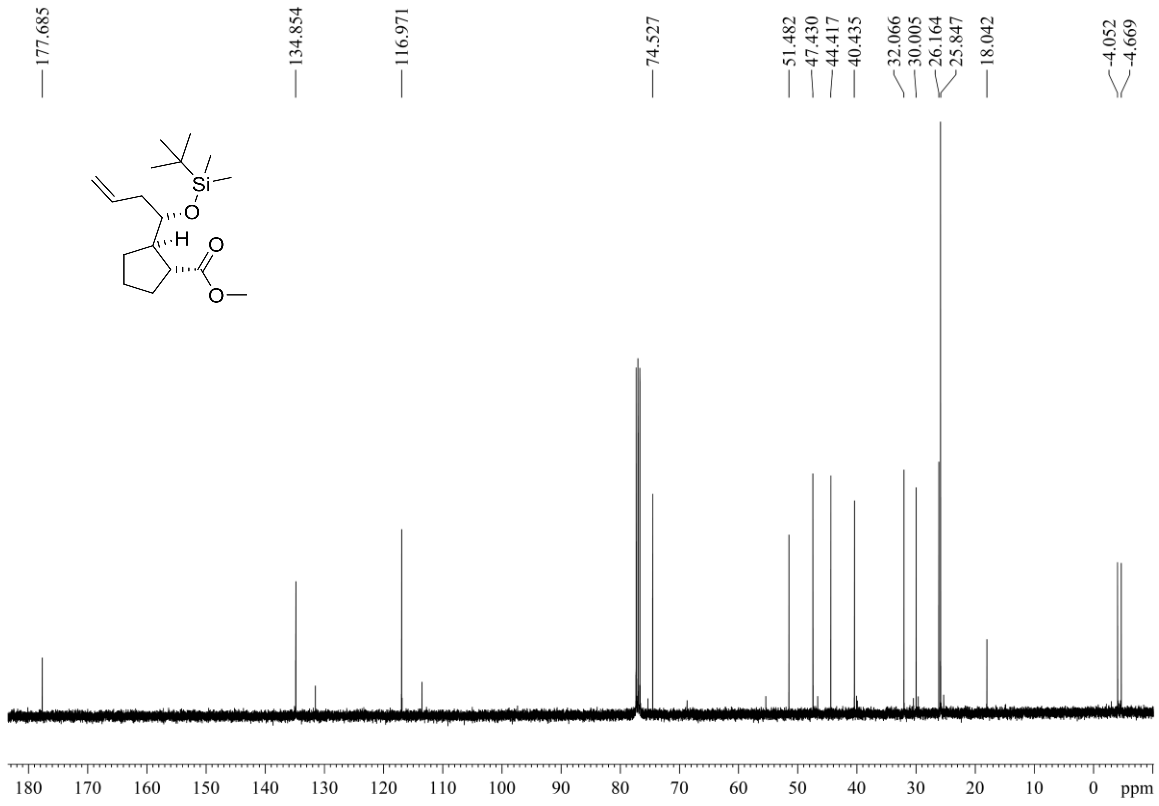

CDCl$_3$, 200 MHz

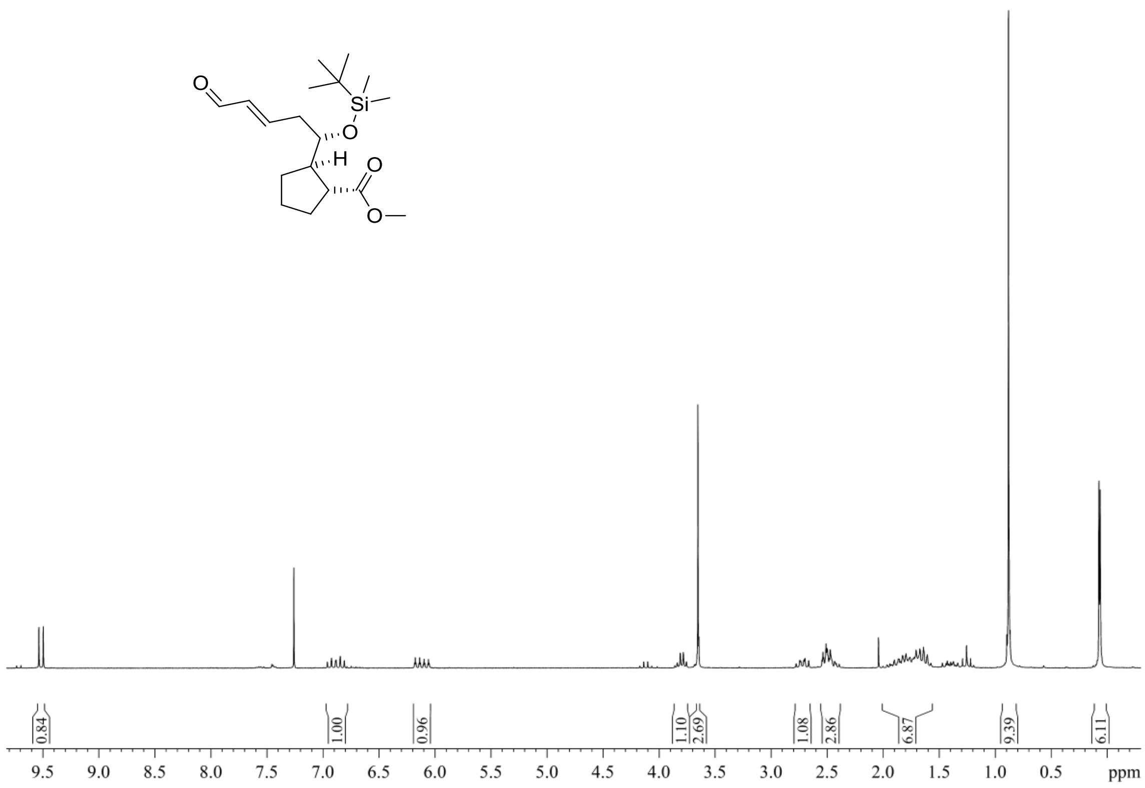

d$_6$-benzene, 400 MHz

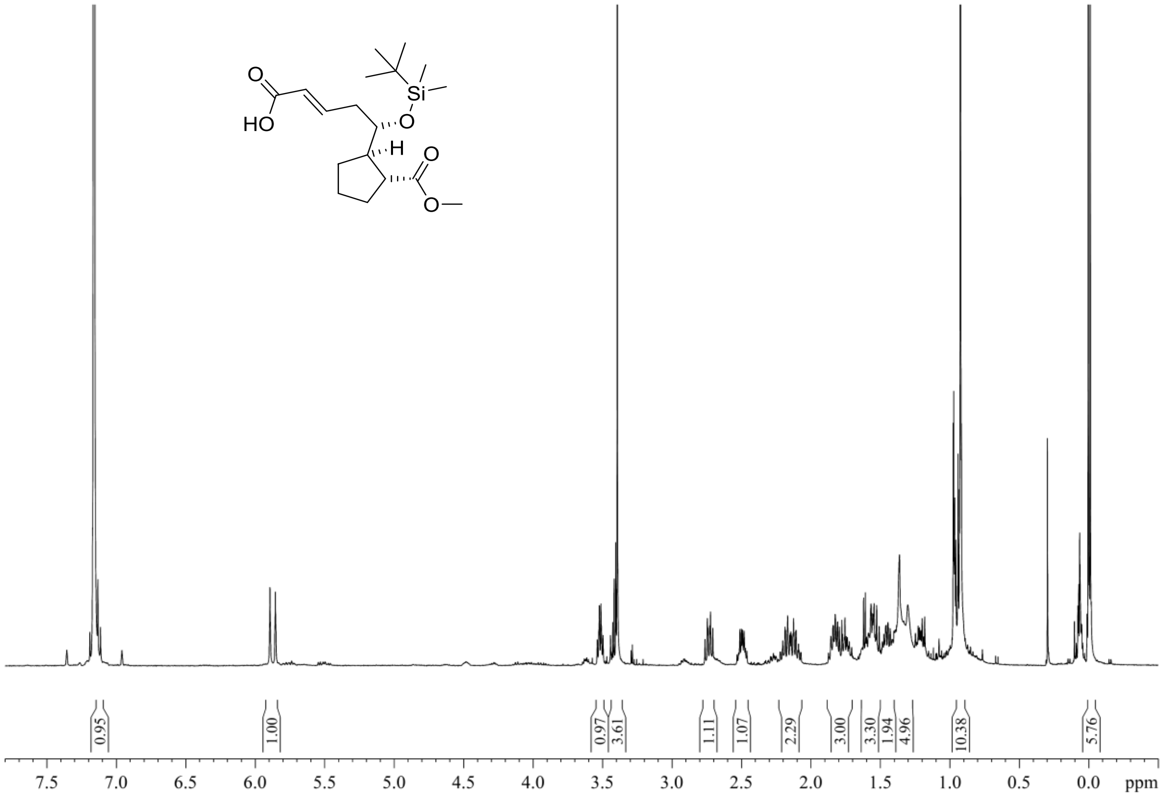

d_6-benzene, 100 MHz

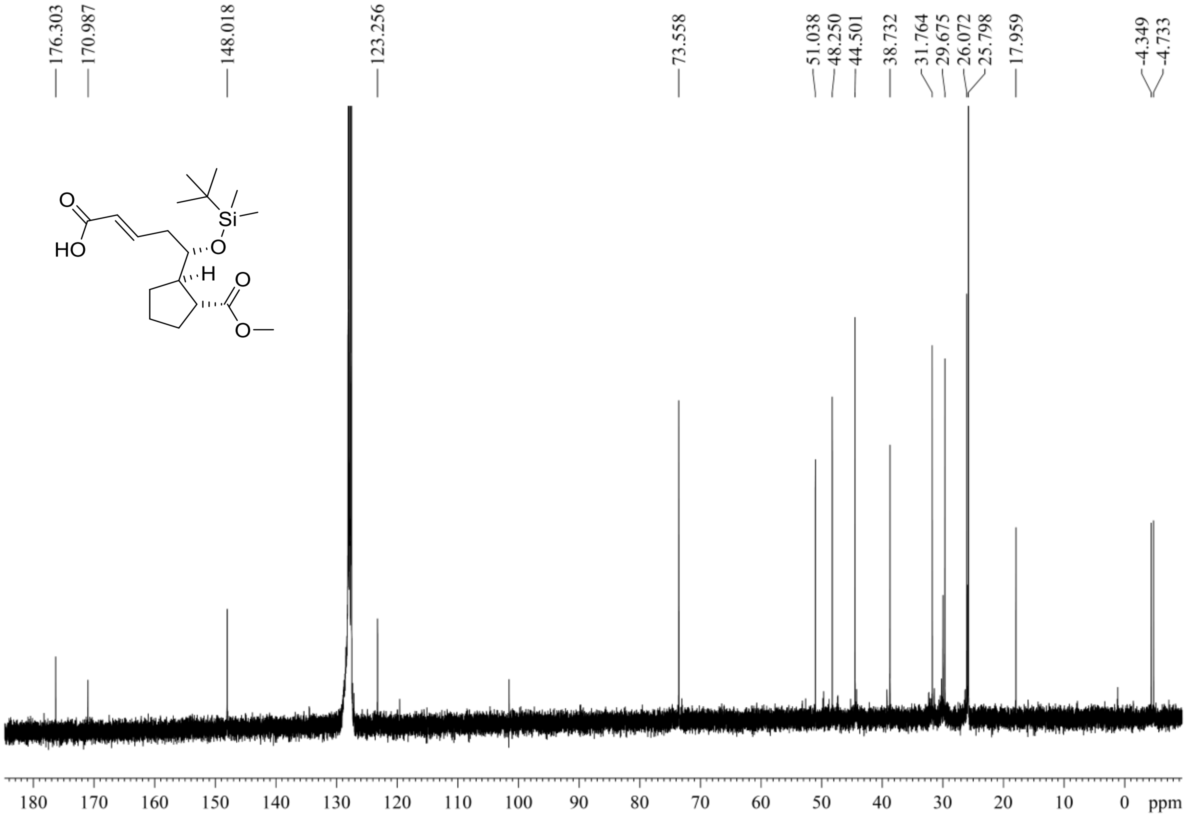

$CDCl_3$, 400 MHz

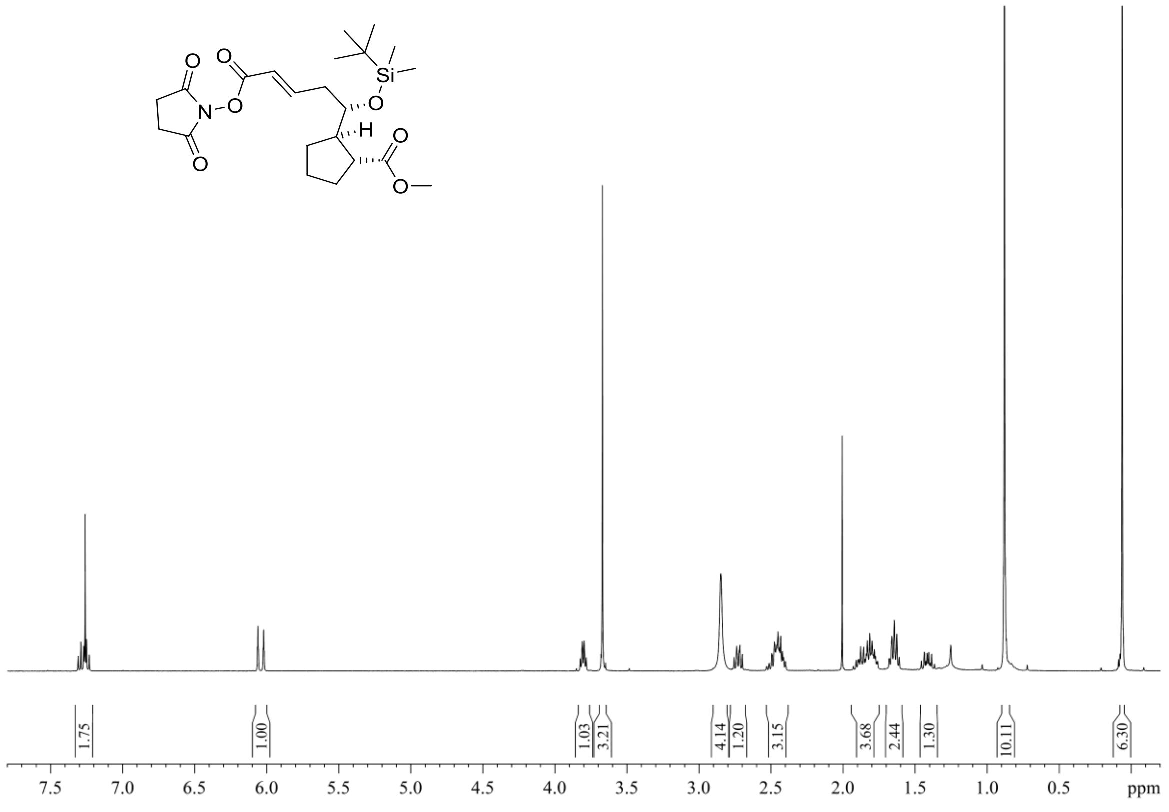

$CDCl_3$, 100 MHz

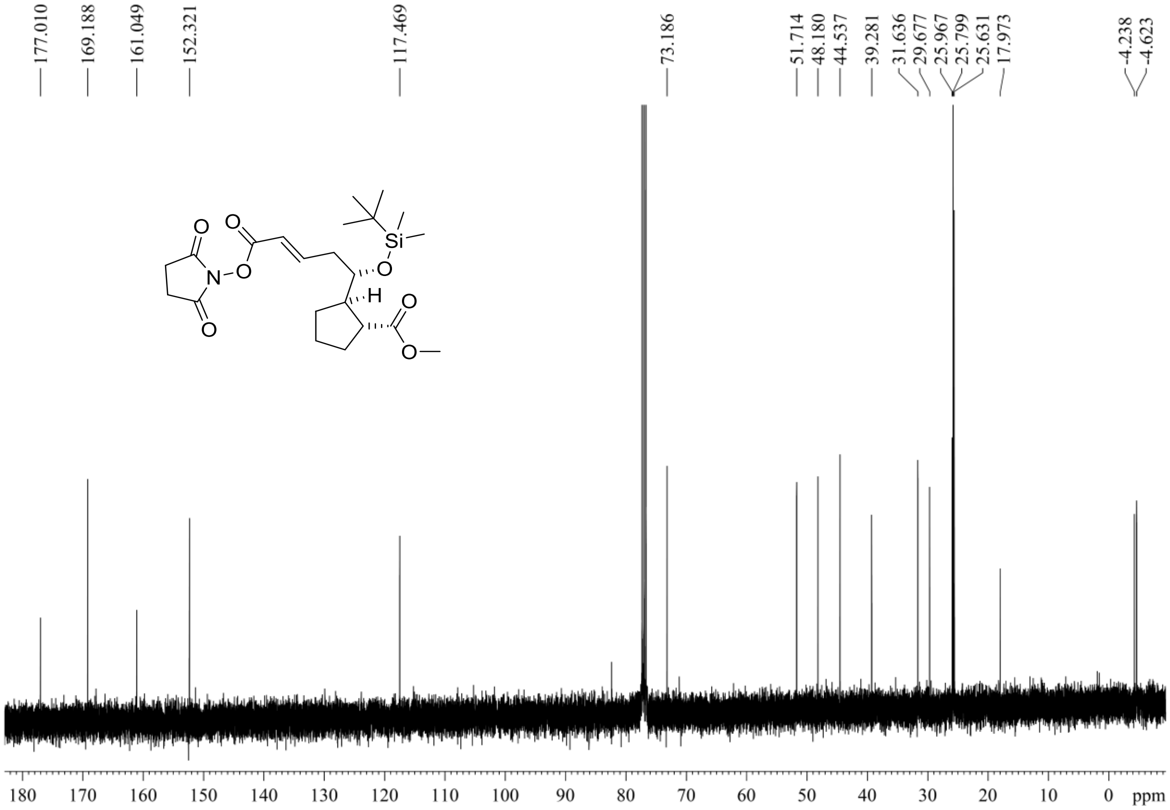

D_2O, 400 MHz

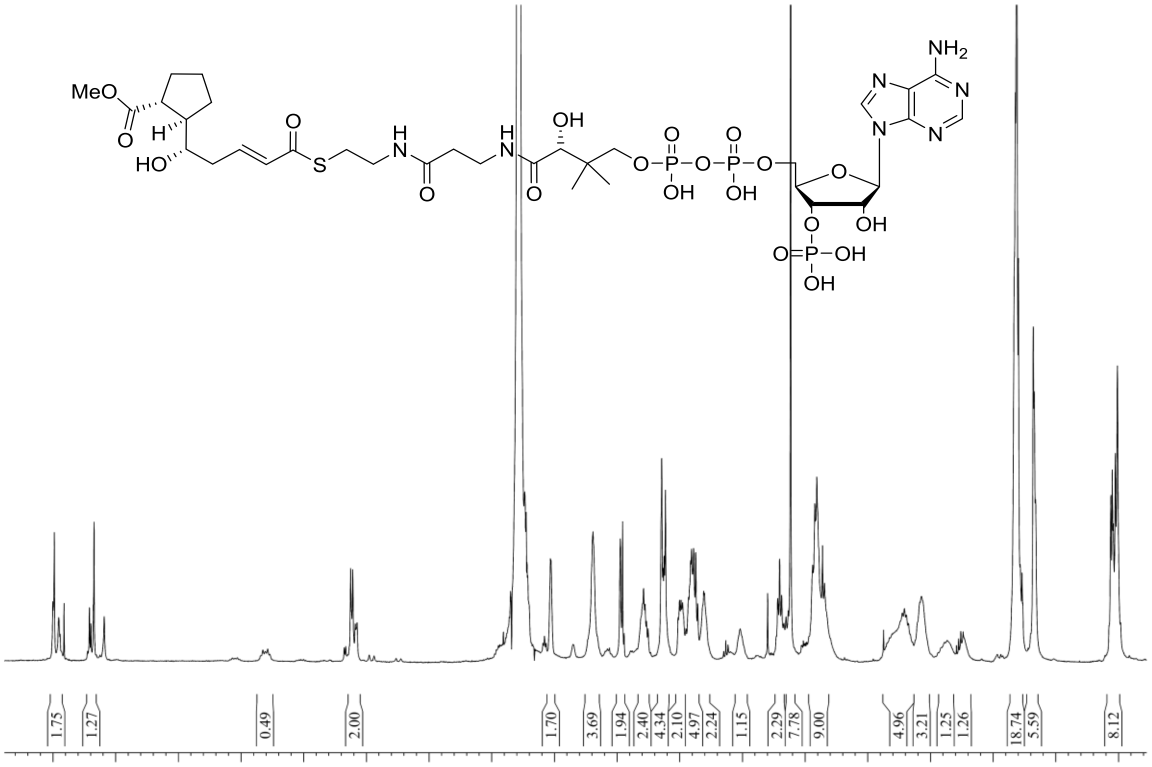

$CDCl_3$, 400 MHz

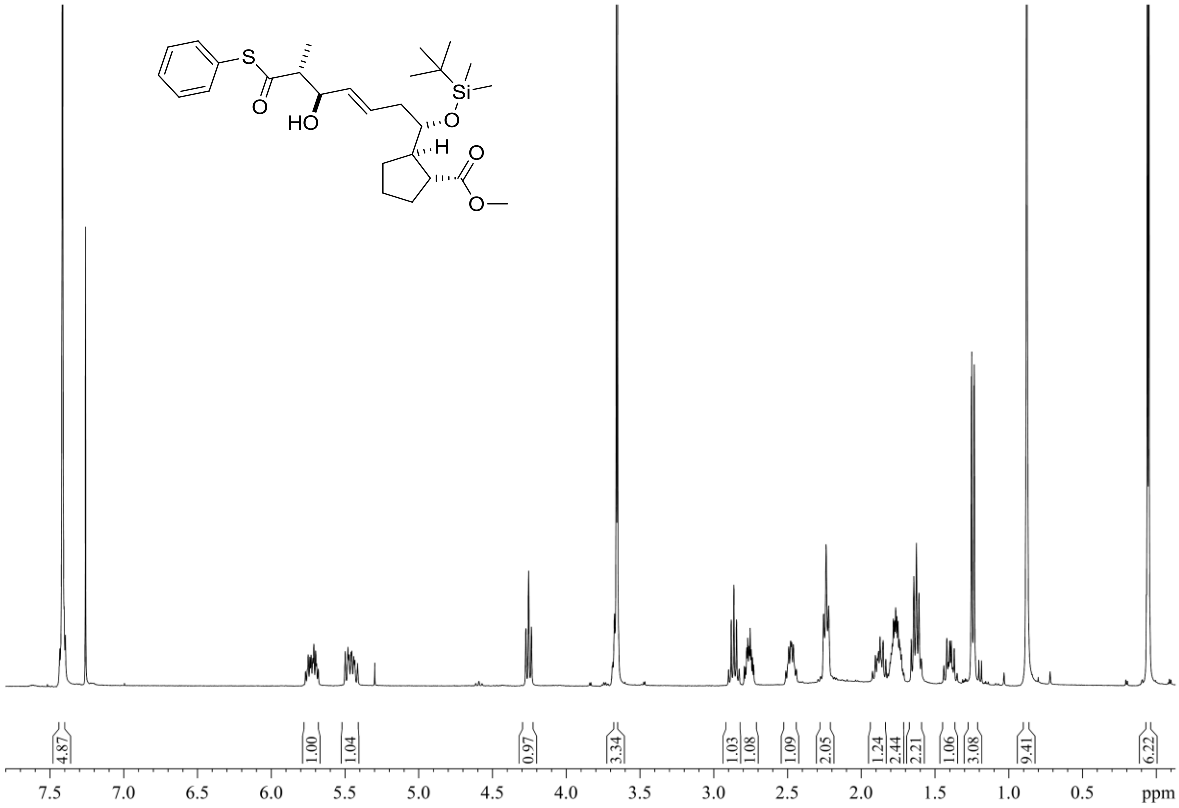

$CDCl_3$, 100 MHz

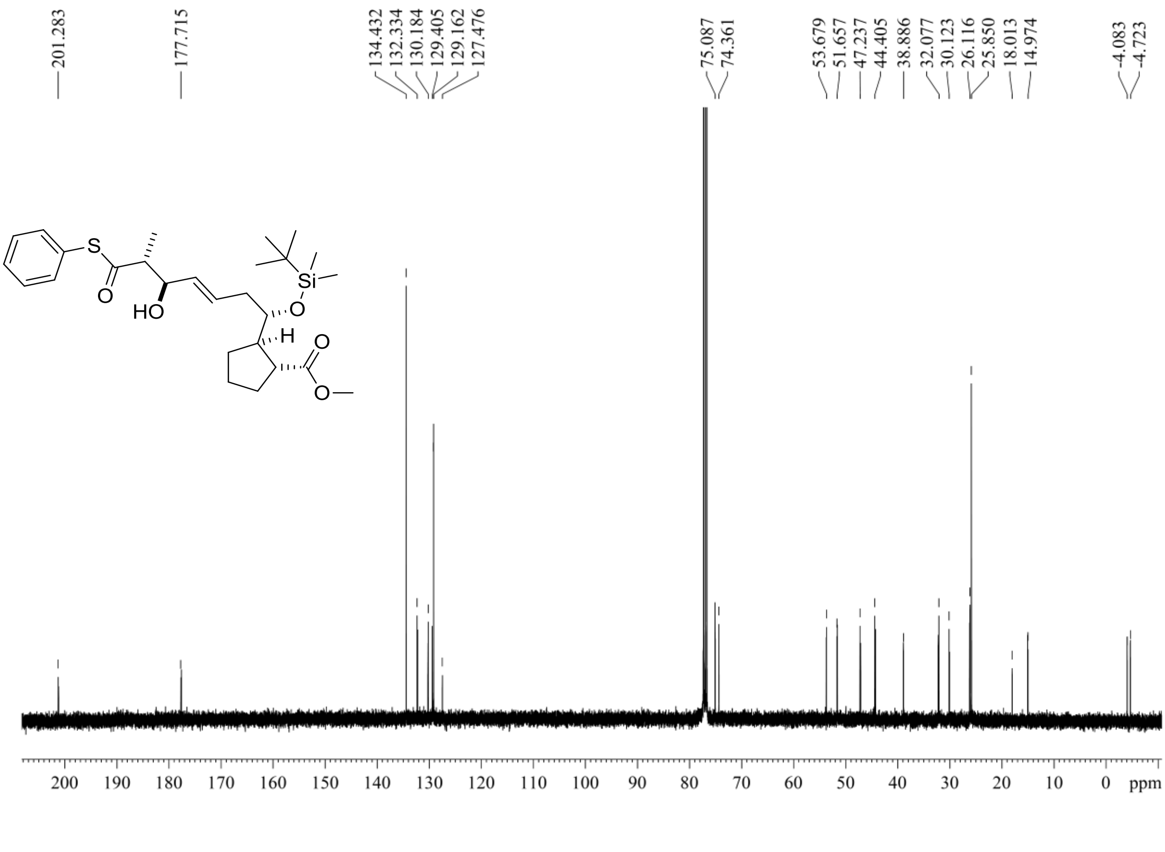

CDCl$_3$, 500 MHz

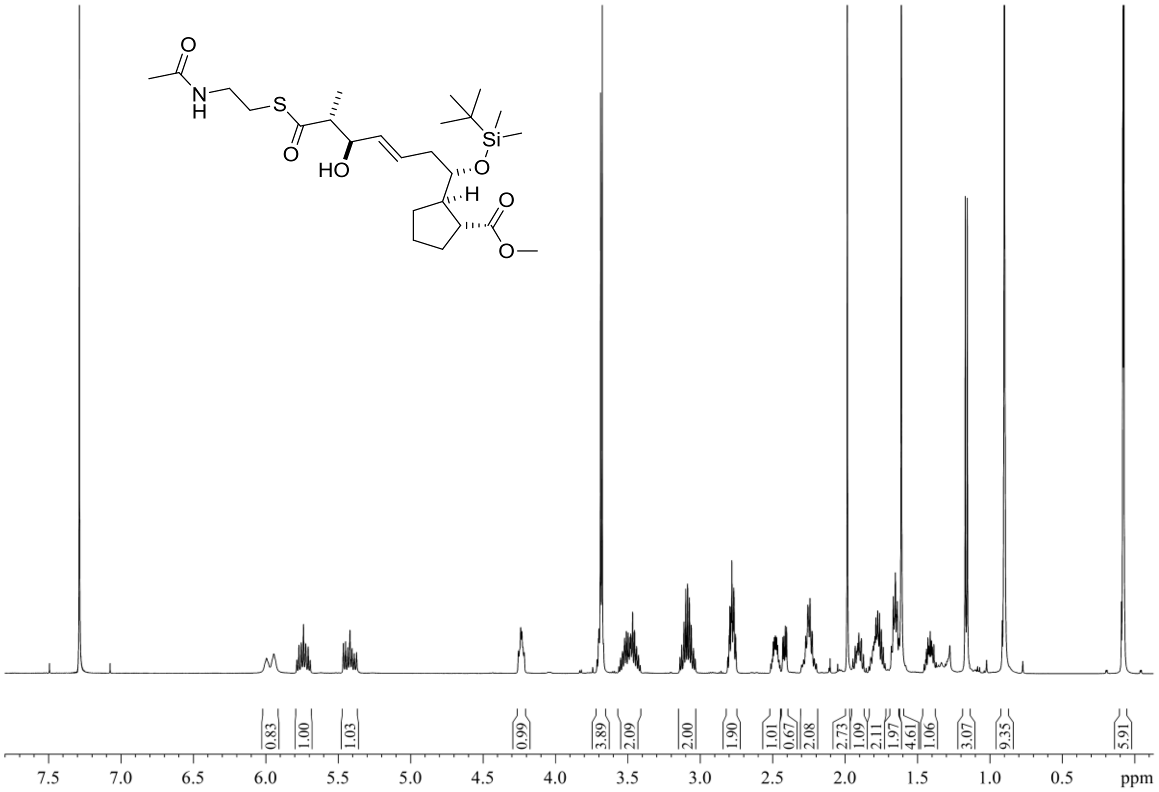

CDCl$_3$, 125 MHz

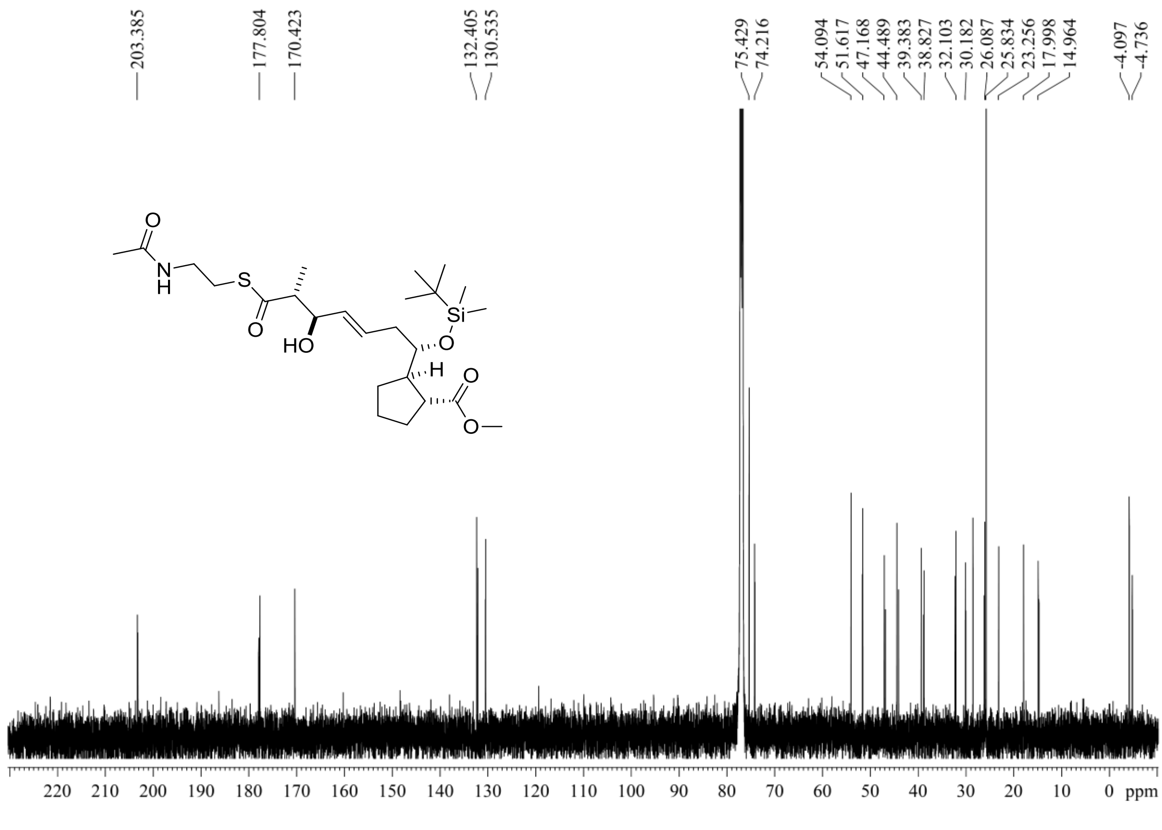

CD_2Cl_2, 400 MHz

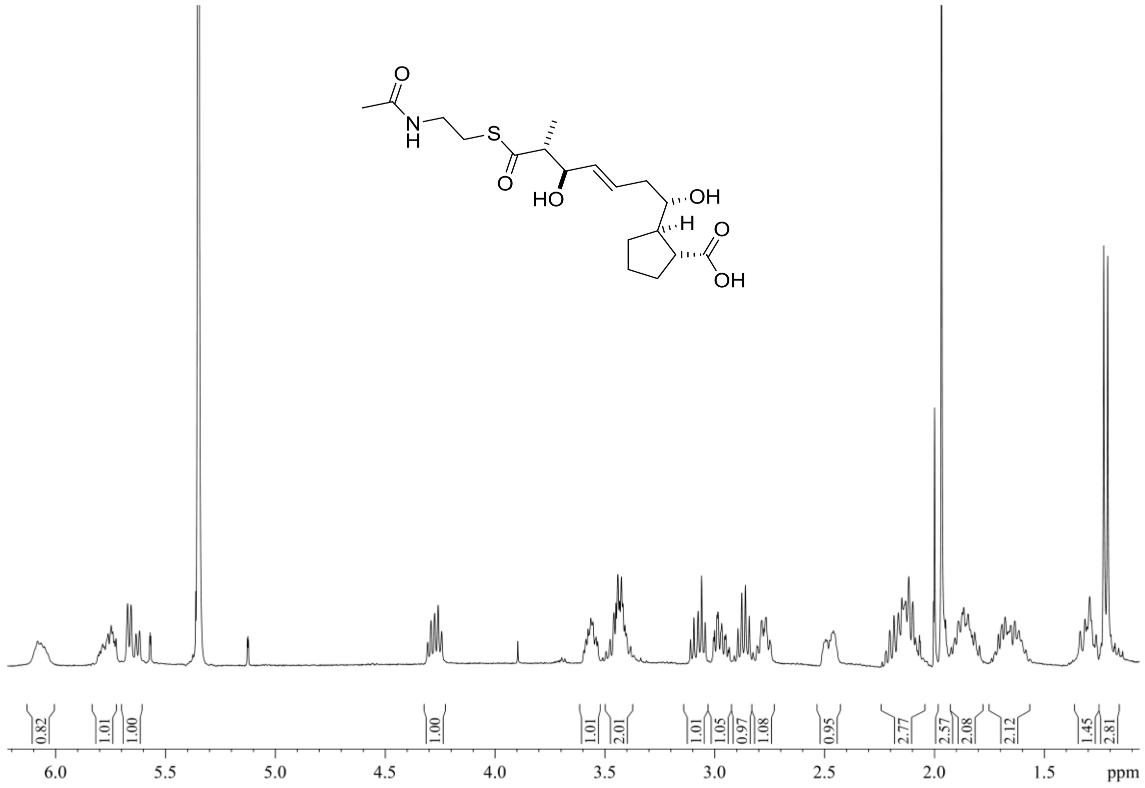

CD_2Cl_2, 100 MHz

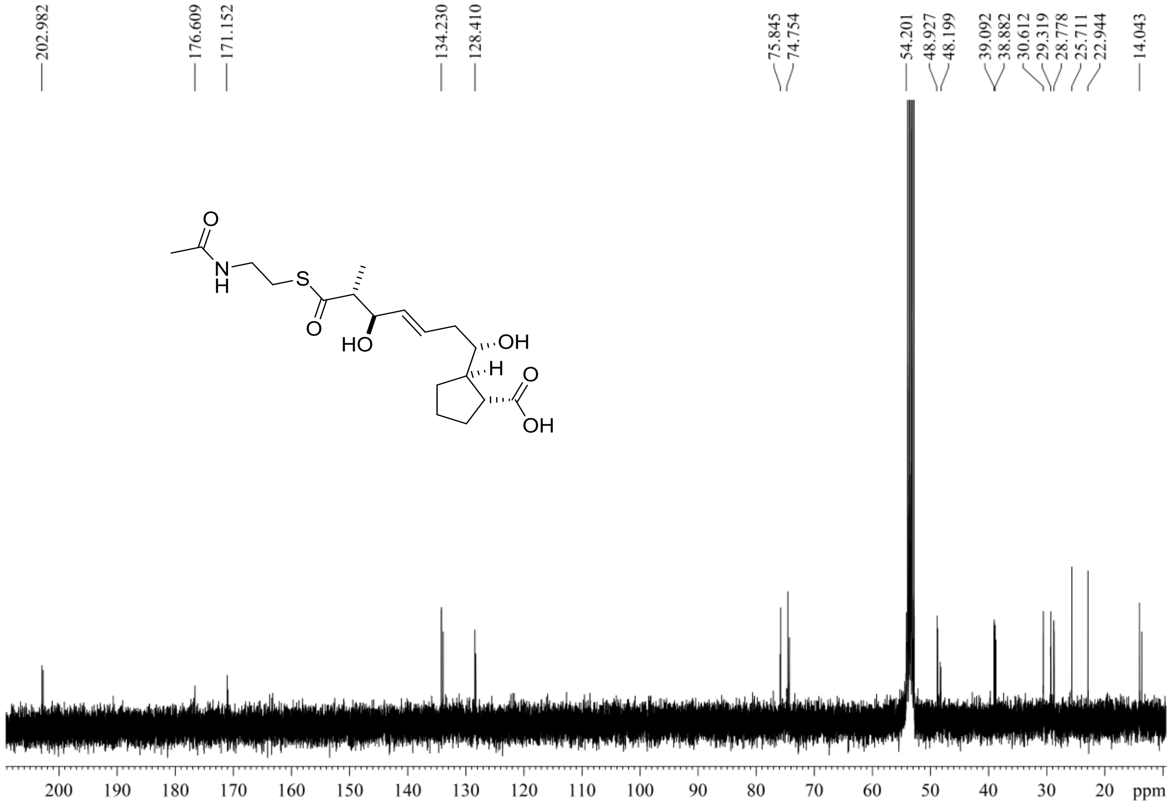

d$_6$-benzene, 500 MHz

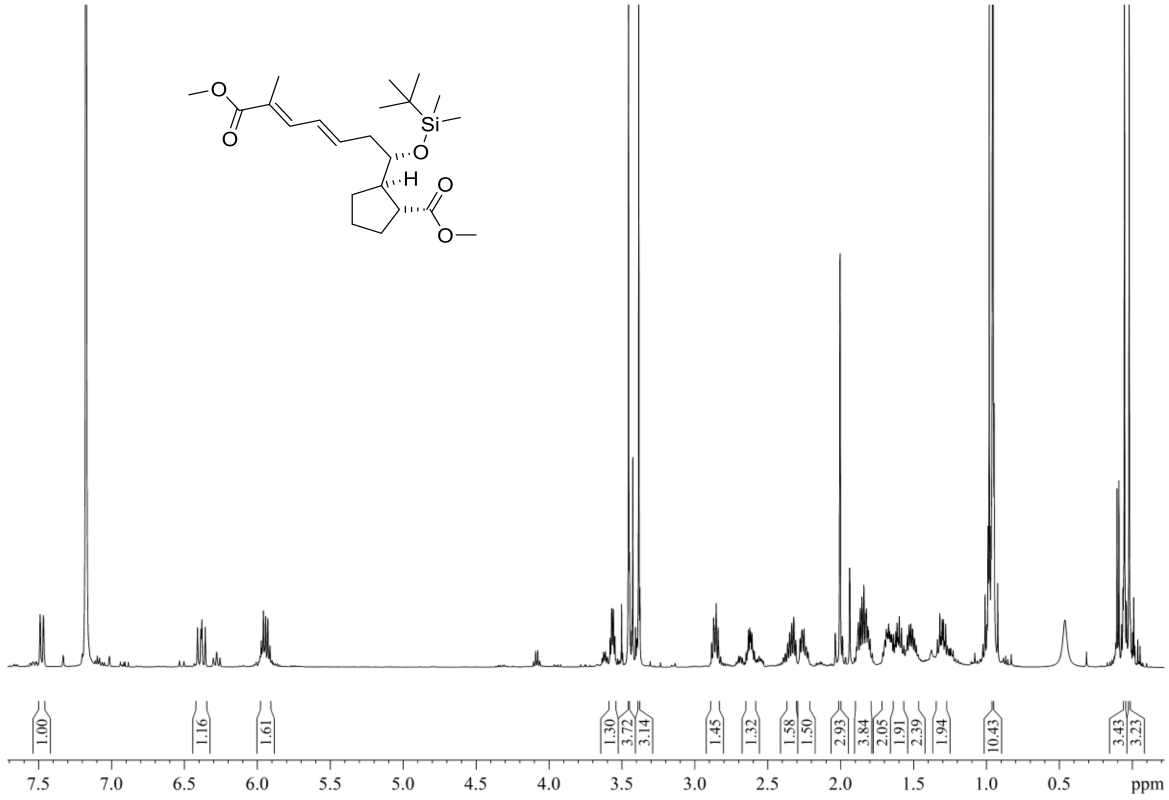

d$_6$-benzene, 125 MHz

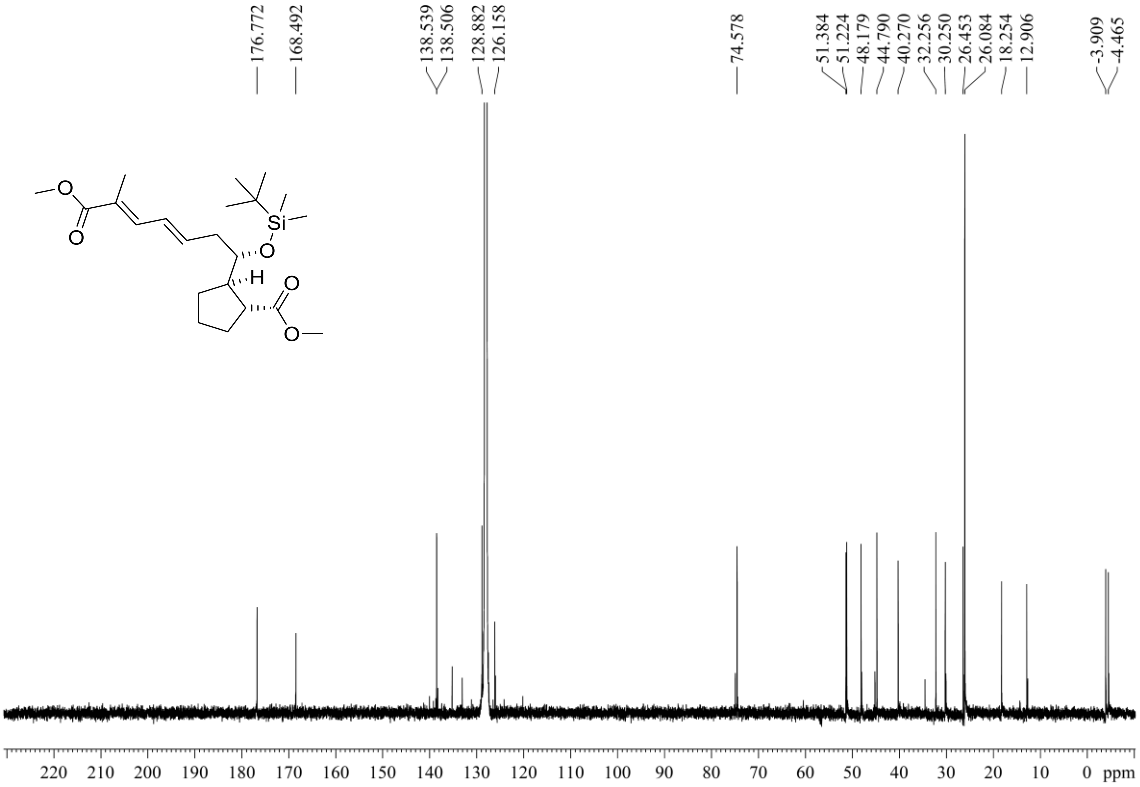

CDCl$_3$, 400 MHz

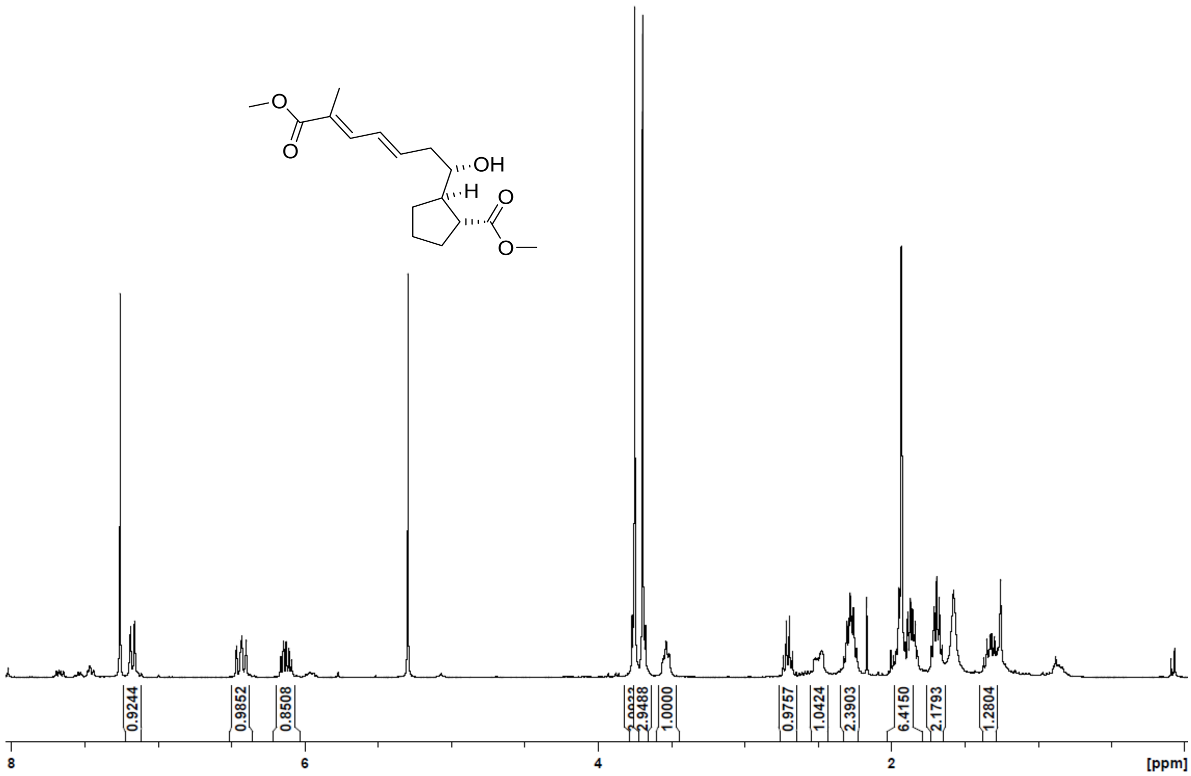

CDCl$_3$, 100 MHz

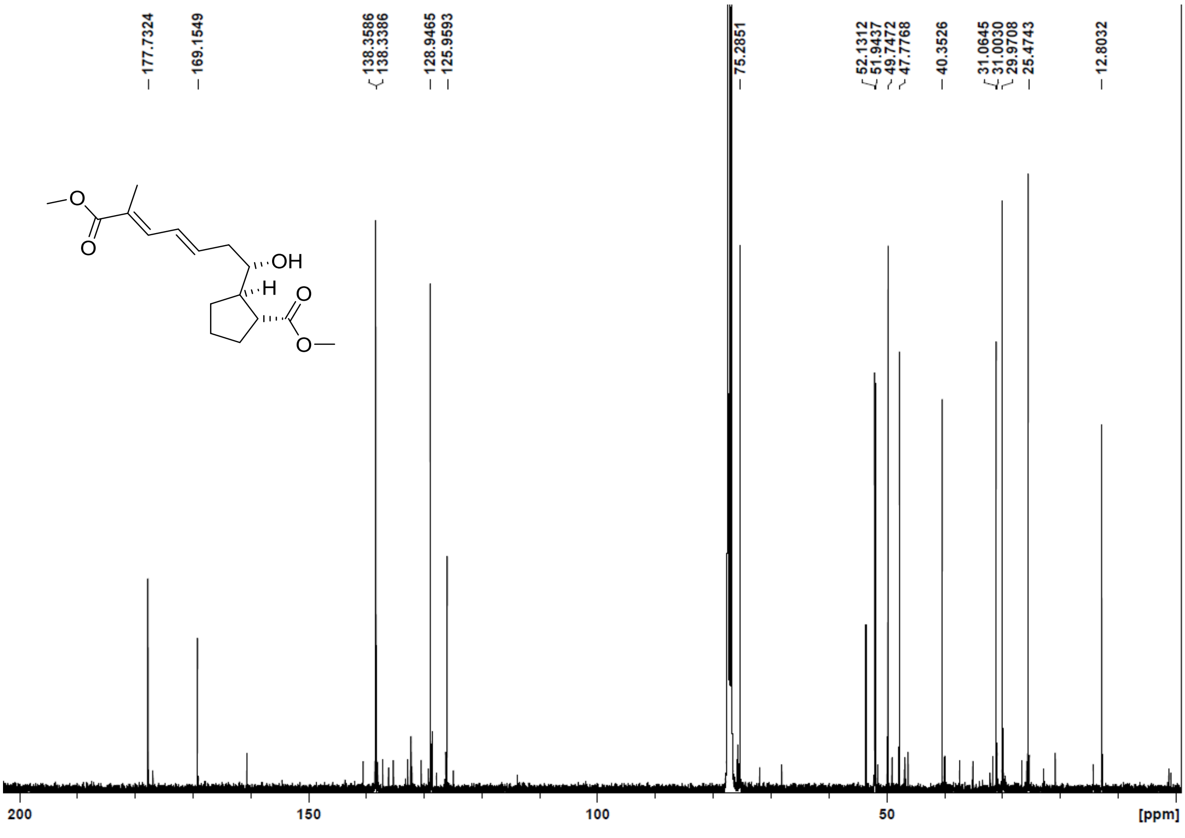

d_6-benzene, 500 MHz

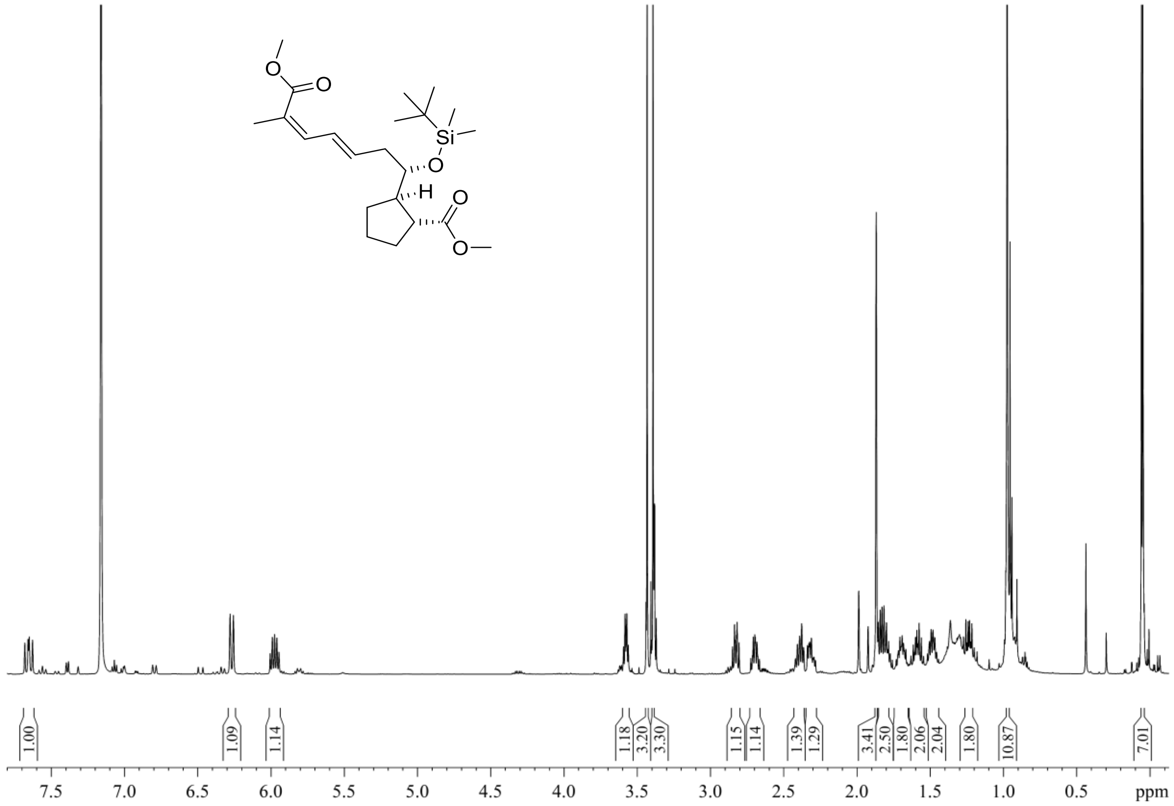

d_6-benzene, 125 MHz

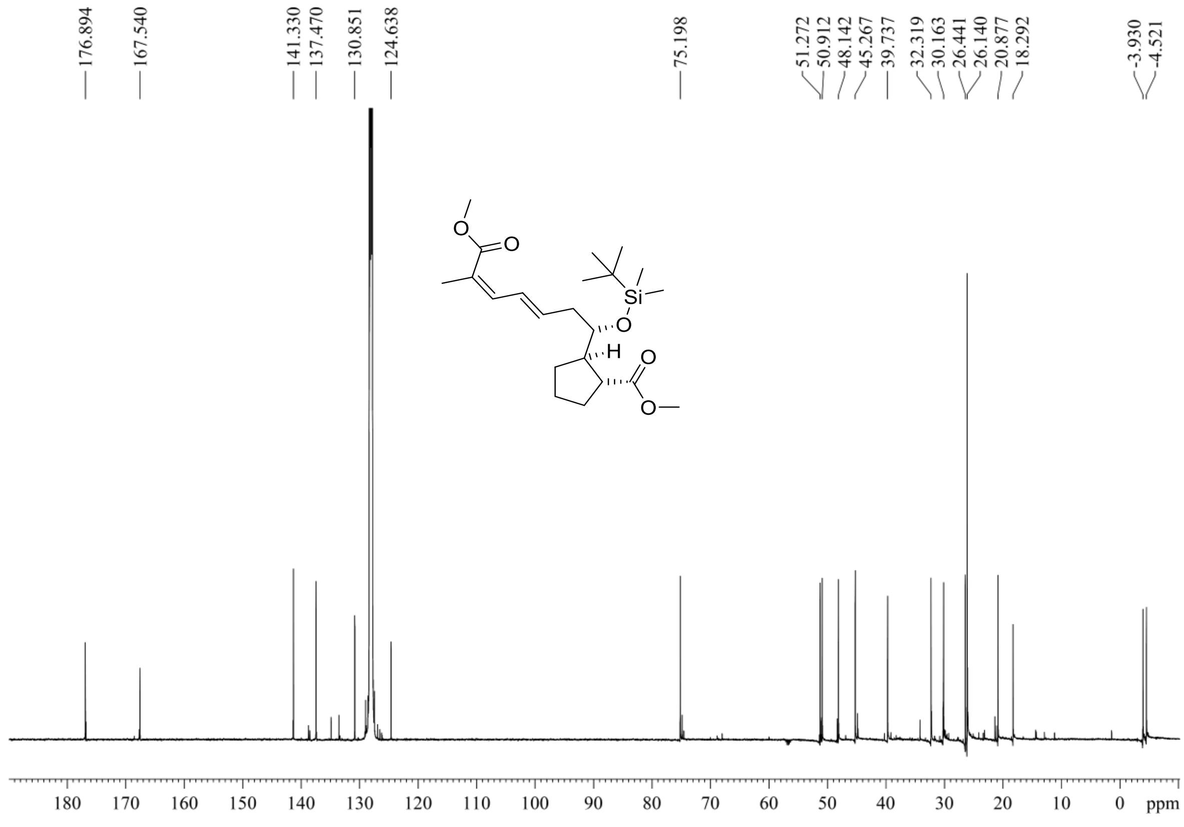

d_6-benzene, 500 MHz

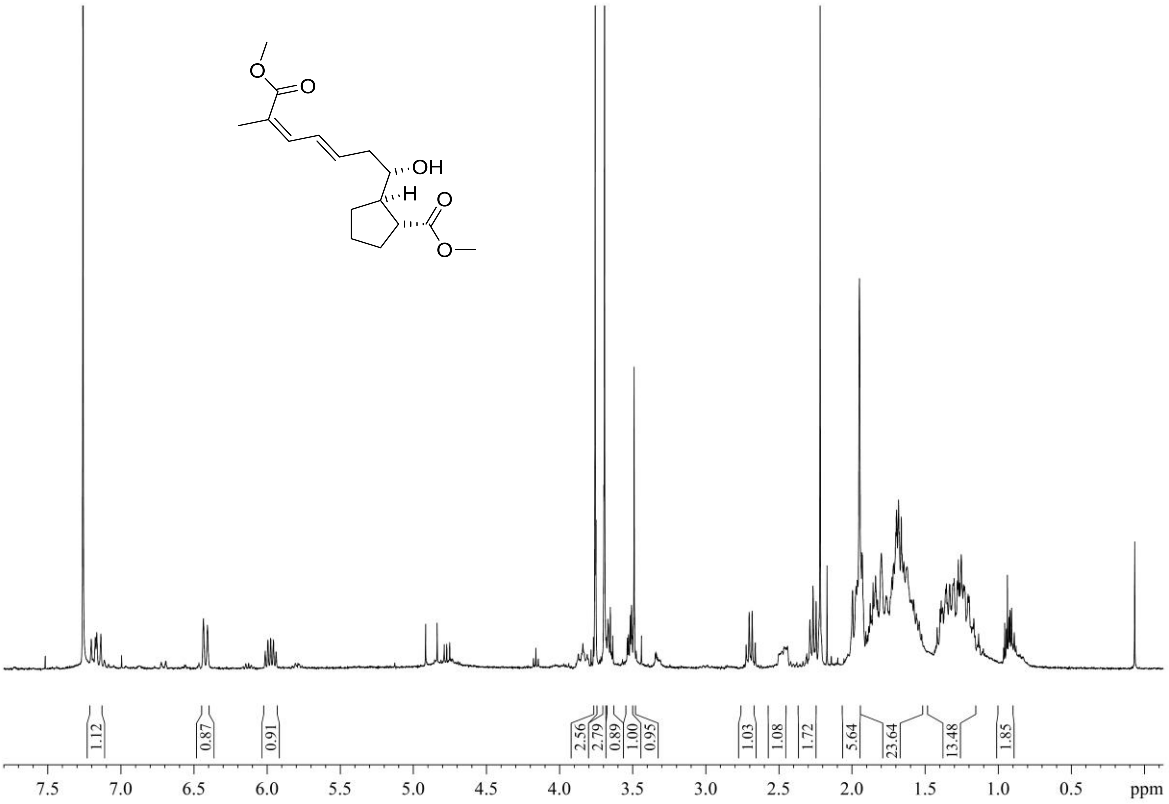

$CDCl_3$, 400 MHz

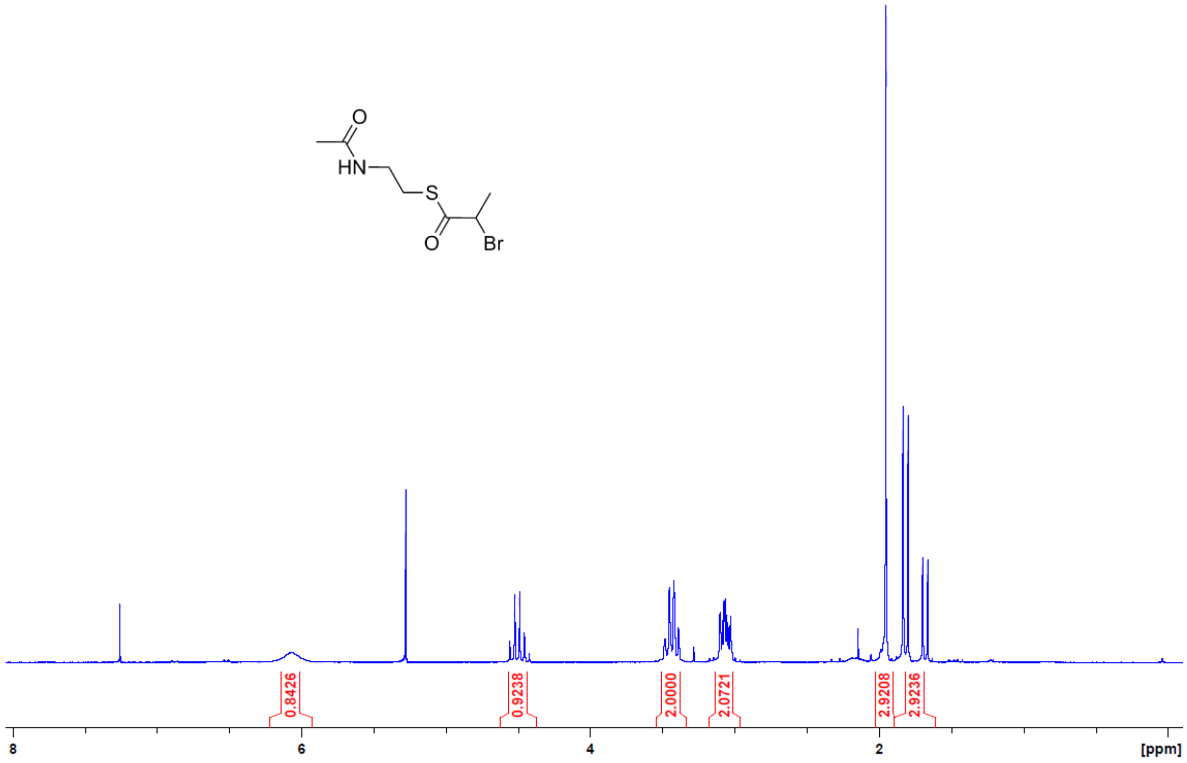

CDCl$_3$, 100 MHz

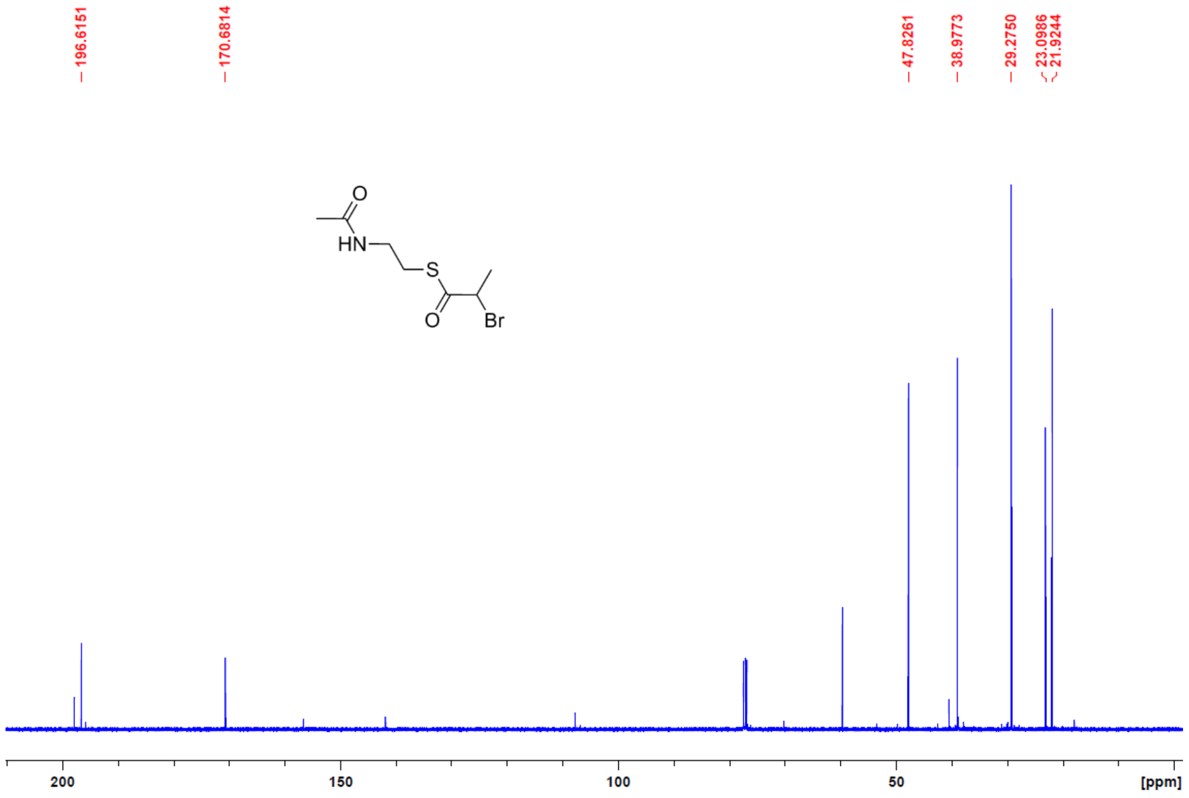

$CDCl_3$, 400 MHz

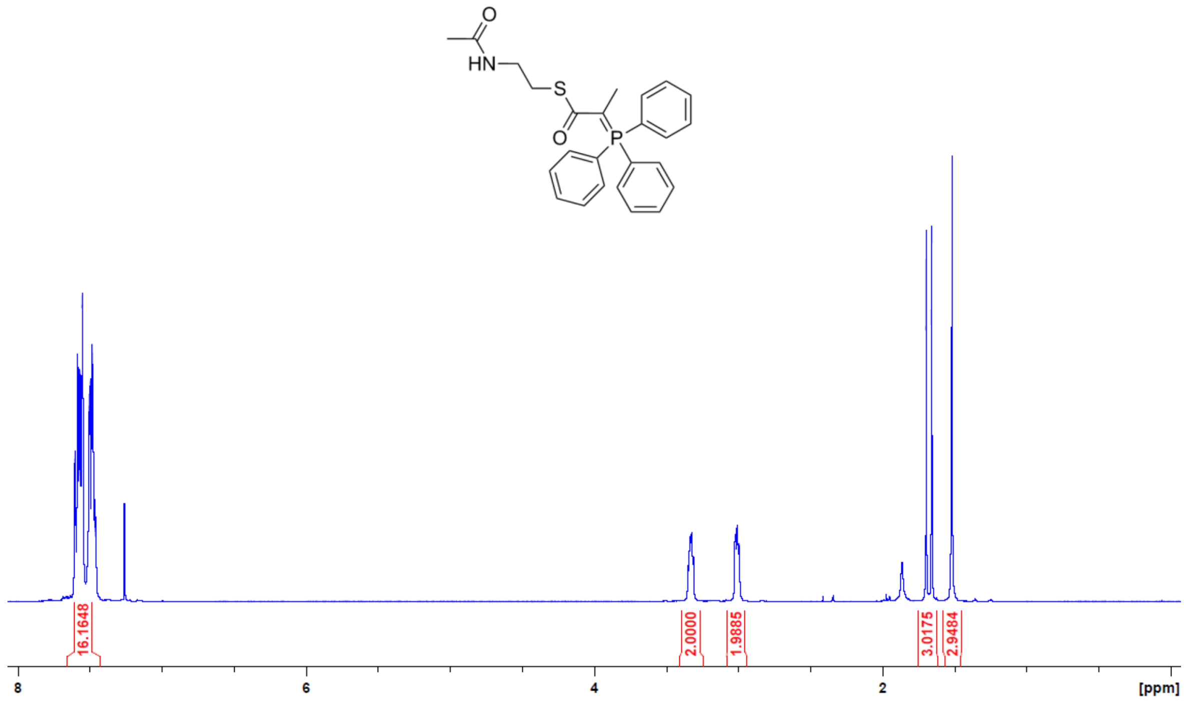

$CDCl_3$, 100 MHz

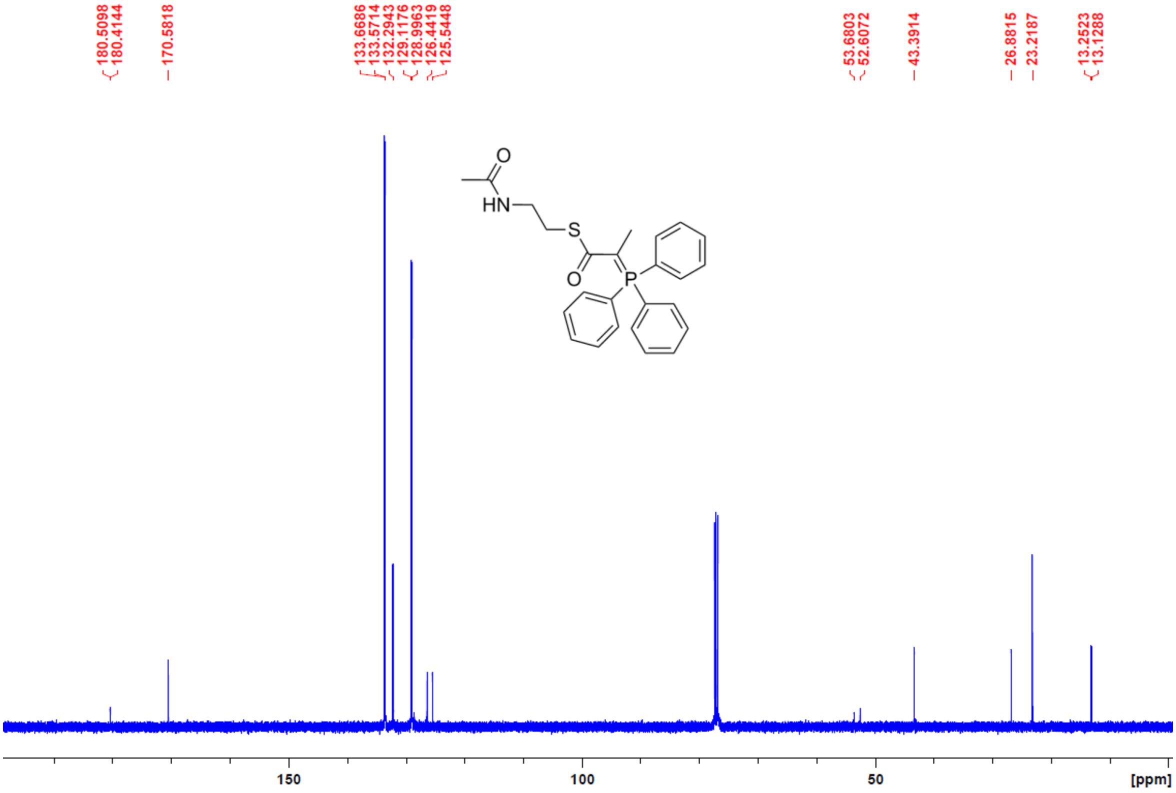

d$_6$-acetone, 500 MHz

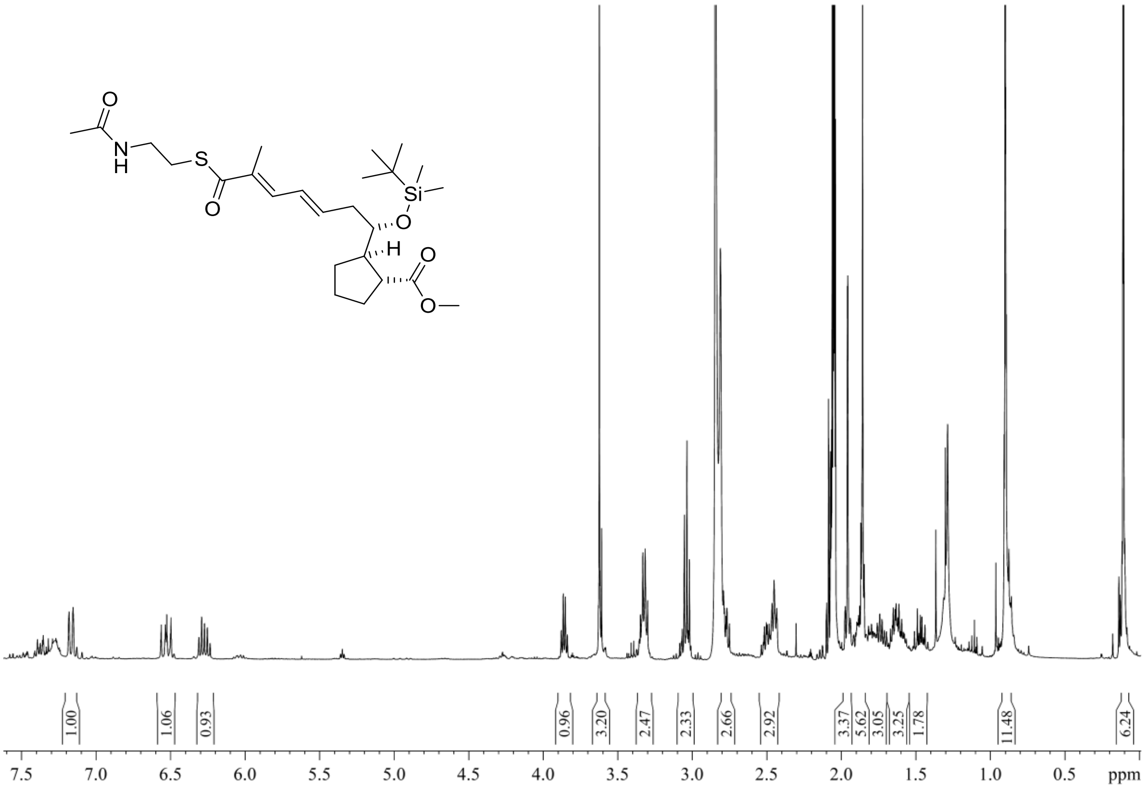

d$_6$-acetone, 125 MHz

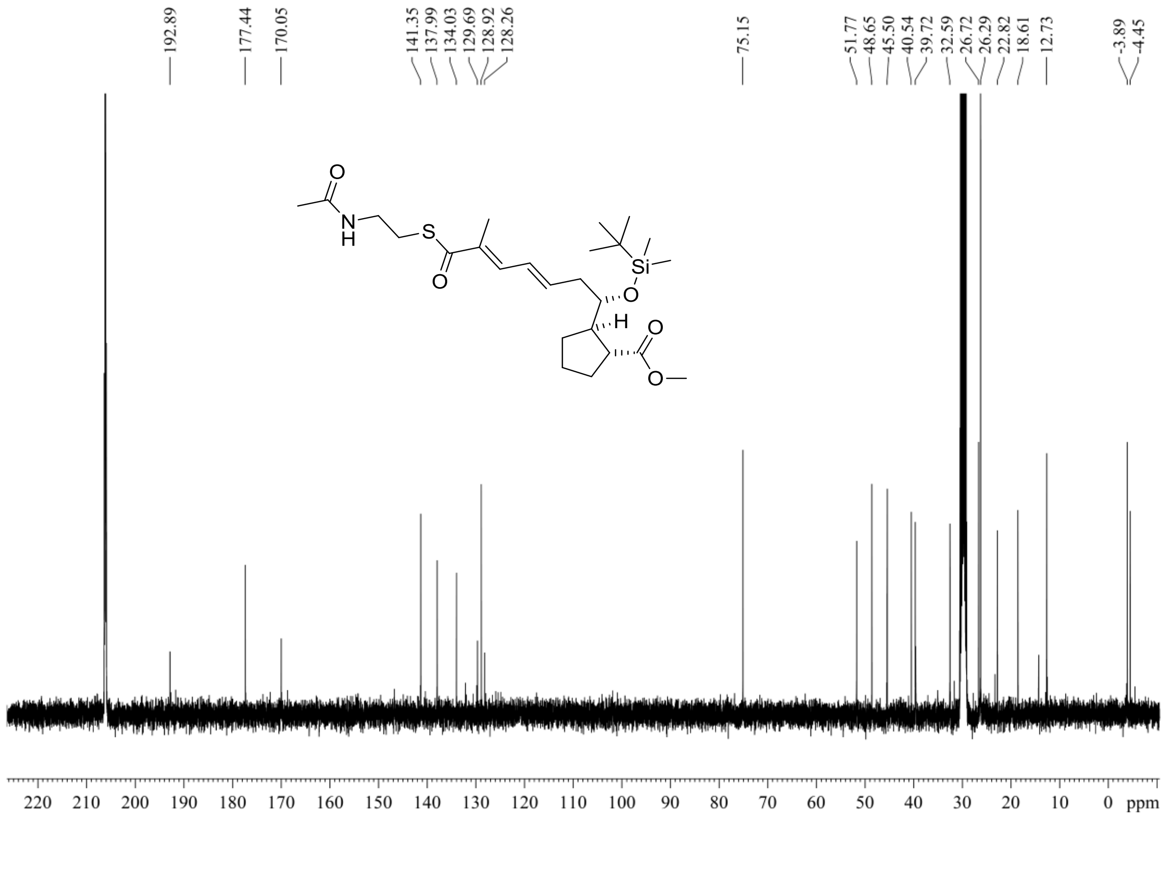

d_6-acetone, 500 MHz

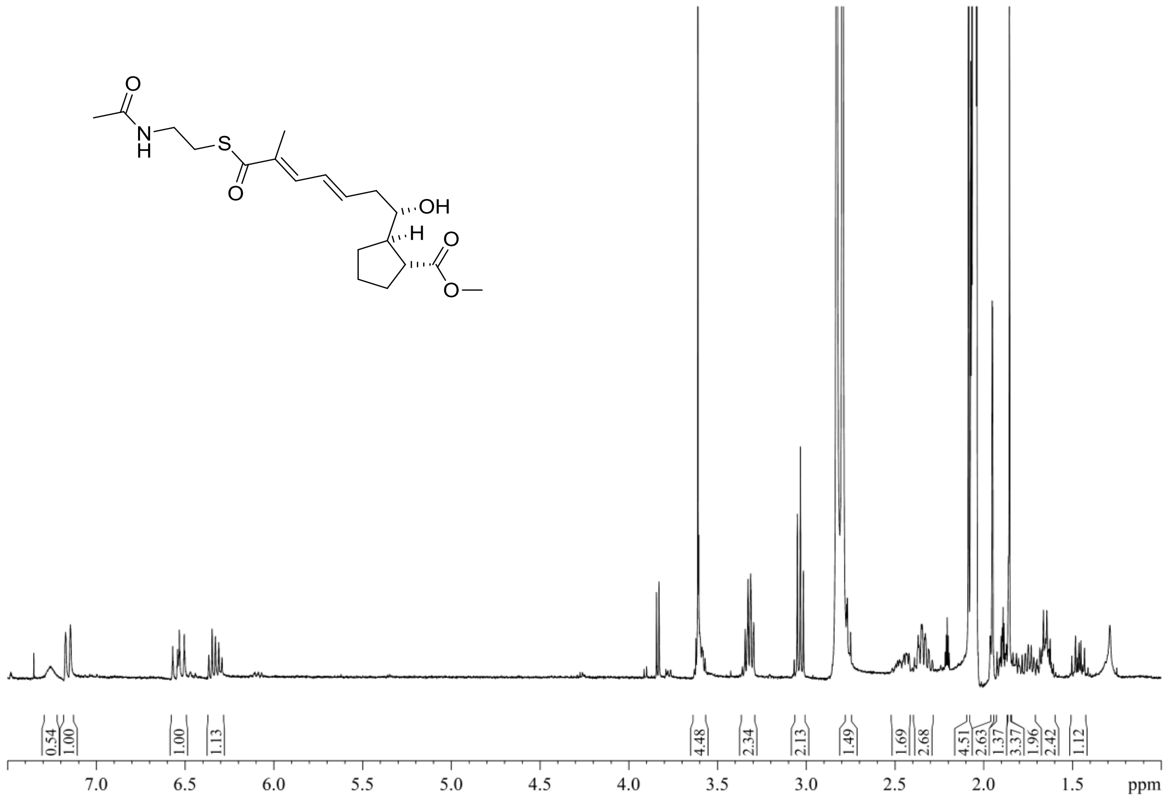

d_6-acetone, 125 MHz

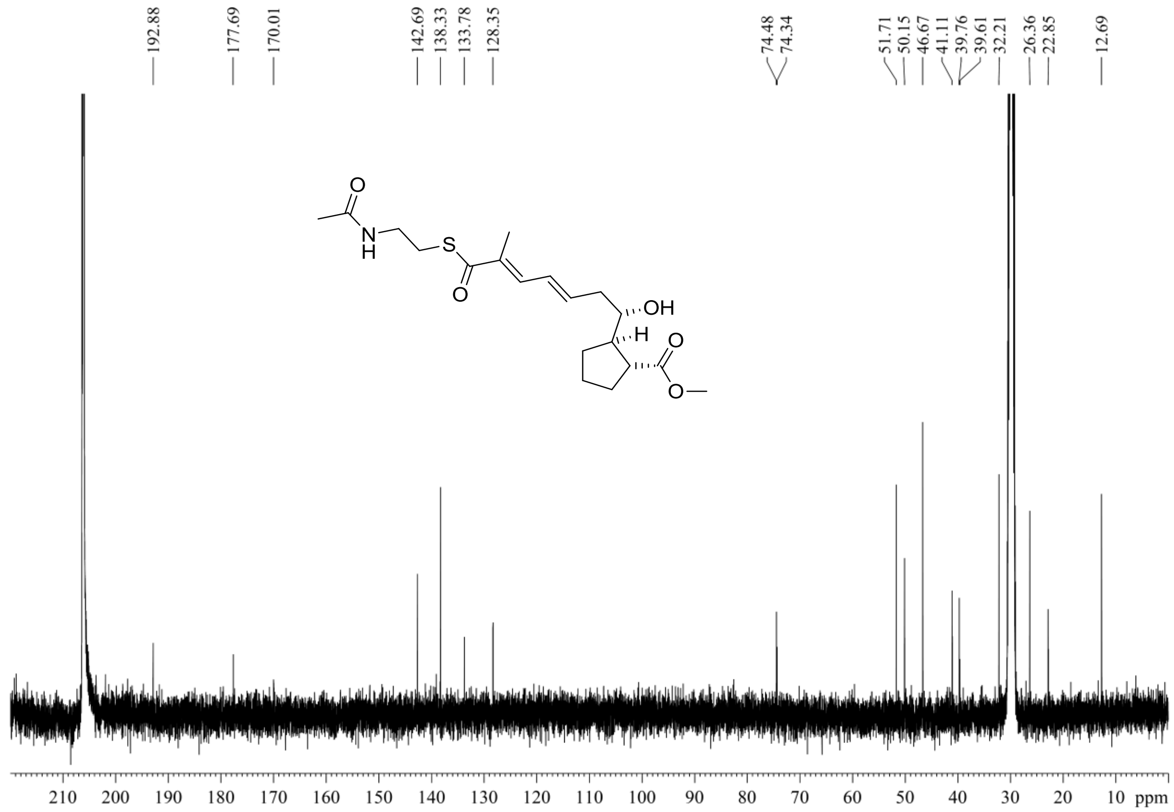

CDCl$_3$, 400 MHz

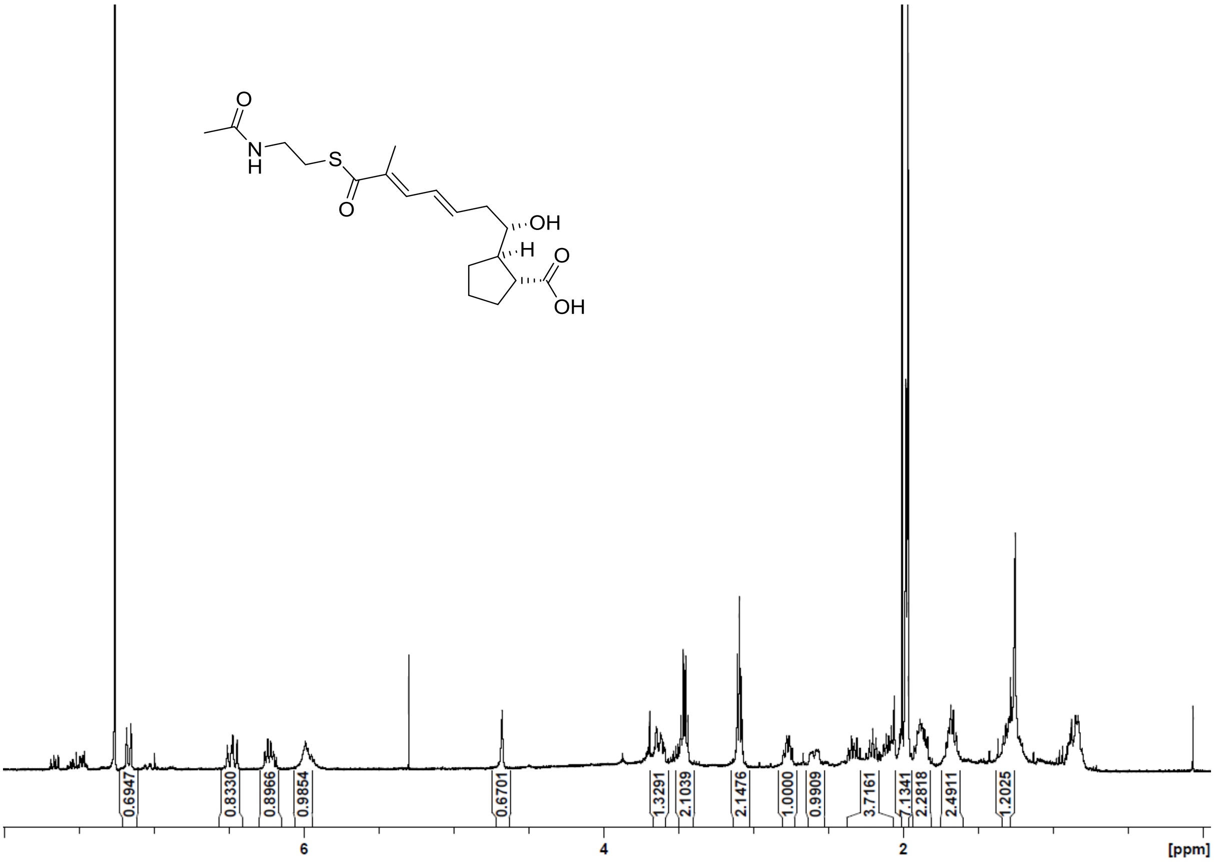

CDCl$_3$, 100 MHz

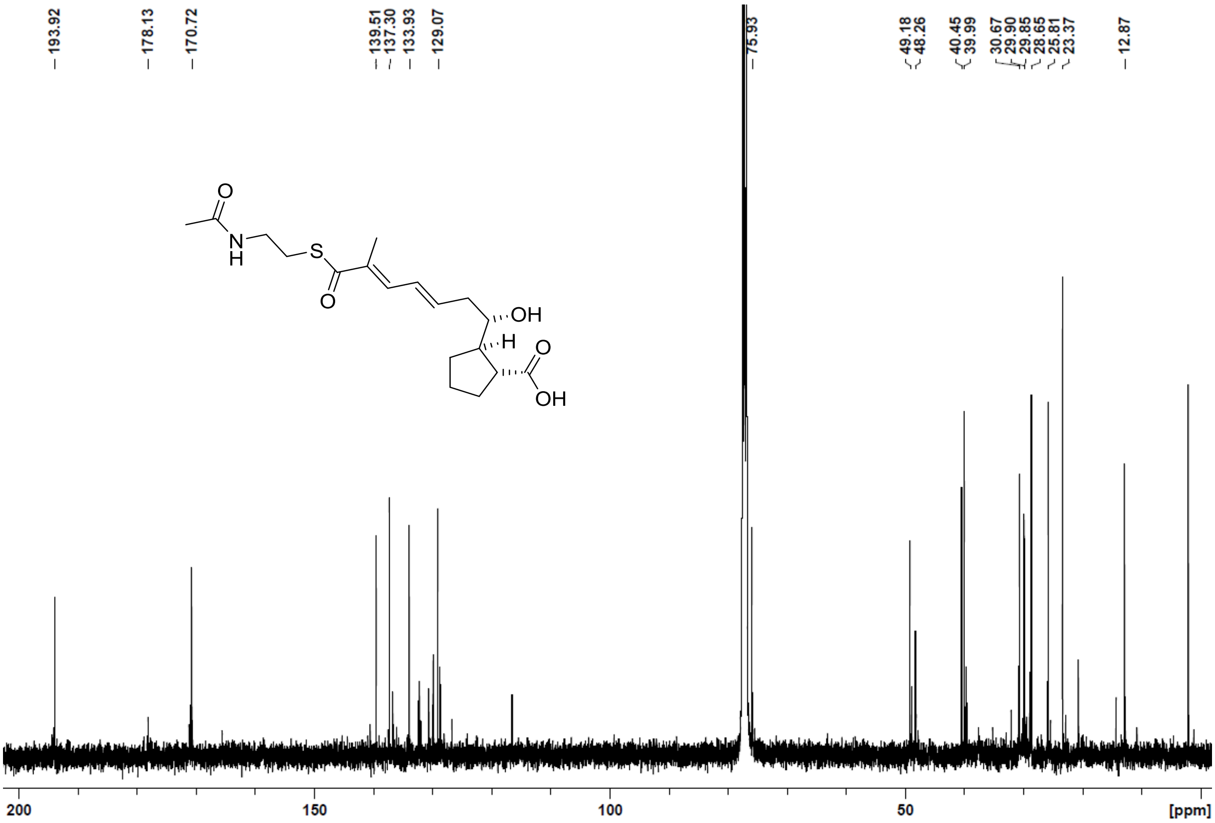

Supplement: File 2 — NMR spectra. [file Beilstein_J_Org_Chem-10-634-s002.pdf]
